# Supplementary material for: Insulin and leptin oscillations license food-entrained browning and metabolic flexibility
Source: Cell Rep. Author manuscript; Available in PMC 2024 Nov 14. (PMC11562929; doi:10.1016/j.celrep.2024.114390)
Supplement: 1 [file NIHMS2011856-supplement-1.pdf]

Cell Reports, Volume *43*

Supplemental information

Insulin and leptin oscillations license

food-entrained browning and metabolic flexibility

Pamela Mattar, Andressa Reginato, Christian Lavados, Debajyoti Das, Manu Kalyani, Nuria Martinez-Lopez, Mridul Sharma, Grethe Skovbjerg, Jacob Lercke Skytte, Urmas Roostalu, Rajasekaran Subbarayan, Elodie Picarda, Xingxing Zang, Jinghang Zhang, Chandan Guha, Gary Schwartz, Prashant Rajbhandari, and Rajat Singh

Supplemental Data Title and Legends

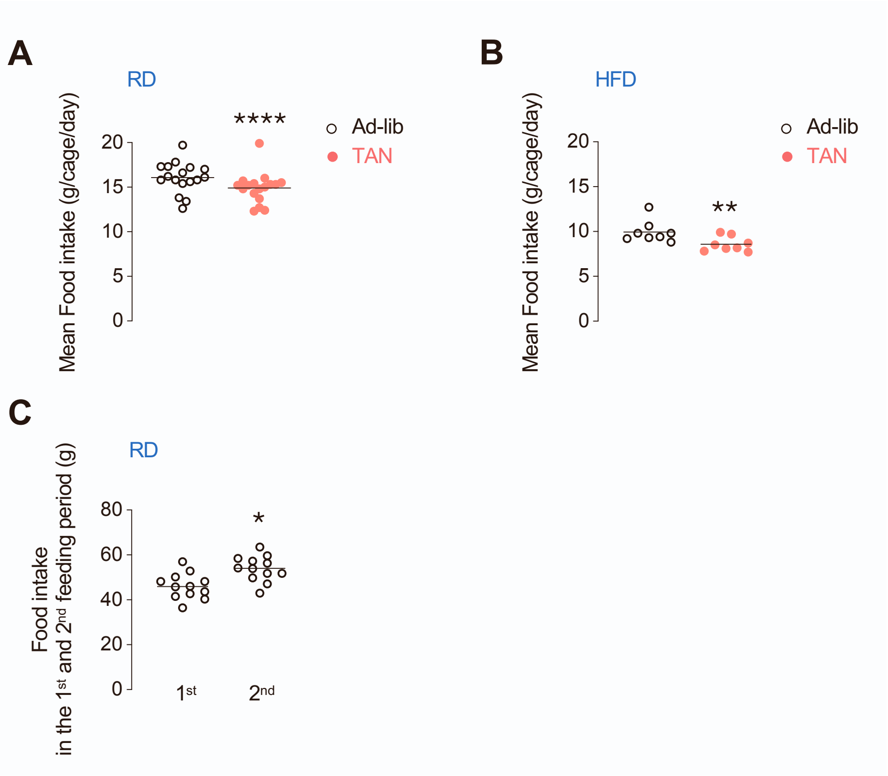

Fig S1 (relates to Fig 1). Food intake assessments in TAN-fed mice.
(**A-B**) Mean food intake (g/cage/day) across each experiment in C57BL6/J male mice fed ad-lib or TAN on RD (n=17) or HFD (n=8).
(**C**) Mean food intake (g) after acclimation in the 1st and 2nd feeding windows in RD-fed C57BL6/J male mice fed ad-lib or TAN (n=12) on RD.
Values are Mean ± SEM. *P<0.05, **P<0.001 ****P<0.0001. Wilcoxon matched-pairs signed rank test (A-C).

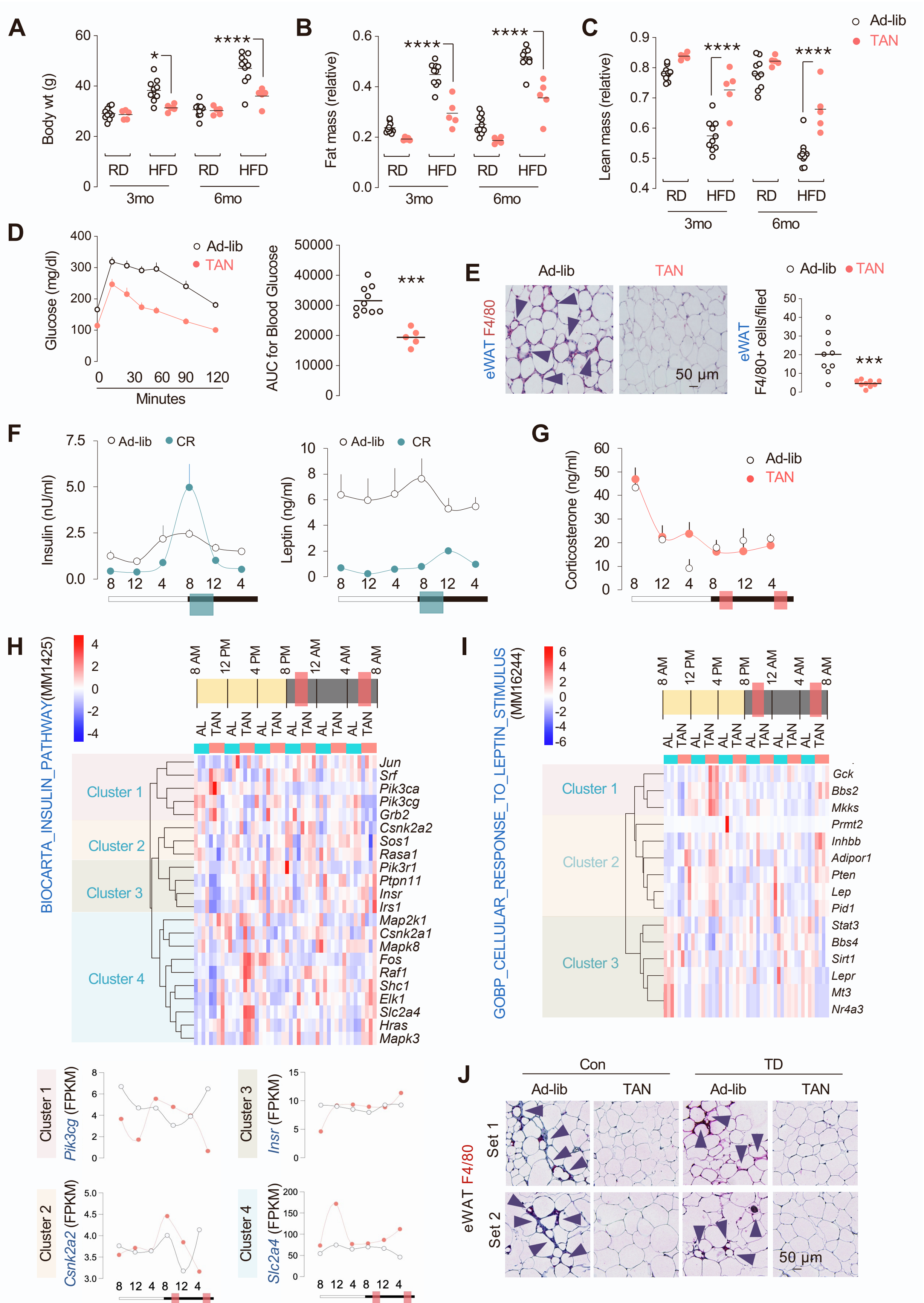
A
Body wt (g)
RD HFD RD HFD
3mo 6mo
B
Fat mass (relative)
C
Lean mass (relative)
Ad-lib
TAN
D
Glucose (mg/dl)
Minutes
AUC for Blood Glucose
E
eWAT F4/80
50 μm
eWAT F4/80+ cells/filed
F
Insulin (nU/ml)
Ad-lib CR
Leptin (ng/ml)
G
Corticosterone (ng/ml)
H
BIOCARTA_INSULIN_PATHWAY(MM1425)
8 AM 12 PM 4 PM 8 PM 12 AM 4 AM 8 AM
AL TAN
Cluster 1
Cluster 2
Cluster 3
Cluster 4
Jun
Srf
Pik3ca
Pik3cg
Grb2
Csnk2a2
Sos1
Rasa1
Pik3r1
Ptpn11
Insr
Irs1
Map2k1
Csnk2a1
Mapk8
Fos
Raf1
Shc1
Elk1
Slc2a4
Hras
Mapk3
I
GOBP_CELLULAR_RESPONSE_TO_LEPTIN_STIMULUS (MM16244)
Gck
Bbs2
Mkks
Prmt2
Inhbb
Adipor1
Pten
Lep
Pid1
Stat3
Bbs4
Sirt1
Lepr
Mt3
Nr4a3
Pik3cg (FPKM)
Insr (FPKM)
Csnk2a2 (FPKM)
Slc2a4 (FPKM)
J
Con
TD
Ad-lib TAN Ad-lib TAN
Set 1
Set 2
eWAT F4/80
50 μm

Figure S2

Fig S2 (relates to Fig 1). Characterizing the protective effect of TAN feeding against obesity, glucose intolerance and inflammation.

(**A-C**) Body weight (g), fat and lean mass (relative to body weight) from C57BL6/J male mice fed ad-lib (n=10) or TAN (n=5) on RD or HFD for 3 or 6 mo.

(**D**) Glucose tolerance test (GTT) and AUC (area under curve) in C57BL6/J male mice fed ad-lib (n=10) or TAN (n=5) on HFD for 6 mo.

(**E**) Representative F4/80 (red) staining in eWAT from C57BL6/J male mice fed ad-lib or TAN on HFD for 6 mo (n=9 each group). Quantification for number of F4/80+ cells/field (1 section/mouse observed with 5X magnification).

(**F**) Serum insulin and leptin levels across 6 time-points each day (24 h) in C57BL6/J male mice in 12 h/12 h light/dark cycle fed ad-lib or caloric restricted (CR, 40% restriction) on RD for 5 mo. Feeding window is label in Y-axis (blue boxes) (n=5 each group).

(**G**) Serum corticosterone levels at 6 time-points in C57BL6/J male mice in 12 h/12 h light/dark cycle fed ad-lib or TAN for 6 mo (n=5 each group). Feeding windows are indicated by *salmon-colored* boxes.

(**H, I**) Bulk RNA-seq analysis of sWAT of ad-lib and TAN-fed mice at 6 Zeitgeber (ZT) time points. Period-wide cluster map of genes associated with insulin pathway (BIOCARTA) **(H)** or response to leptin (GO_BP) **(I)** for both ad-lib and TAN group (n=4 each group). Z-score normalized values were plotted and implemented for hierarchical clustering. Red denotes upregulation, blue denotes downregulation.

(J) Representative F4/80 (red) staining in eWAT of C57BL6/J male mice fed ad-lib or TAN on HFD for 4 mo and housed in 12-12 h light/dark cycle (Con) or 24 h total darkness (TD) (n=5 each group). Quantification for number of F4/80+ cells/field (1 section/mouse observed with 5X magnification).

Dot plots show individual values (dots) and mean (line). Every plot across 24 h shows the mean value (dots) per time-point ± SEM. *P<0.05, ***P<0.001 ****P<0.0001. Two-way ANOVA and Tukey-corrected (A, B, C), and two-tailed unpaired Student’s t-test (D, E).

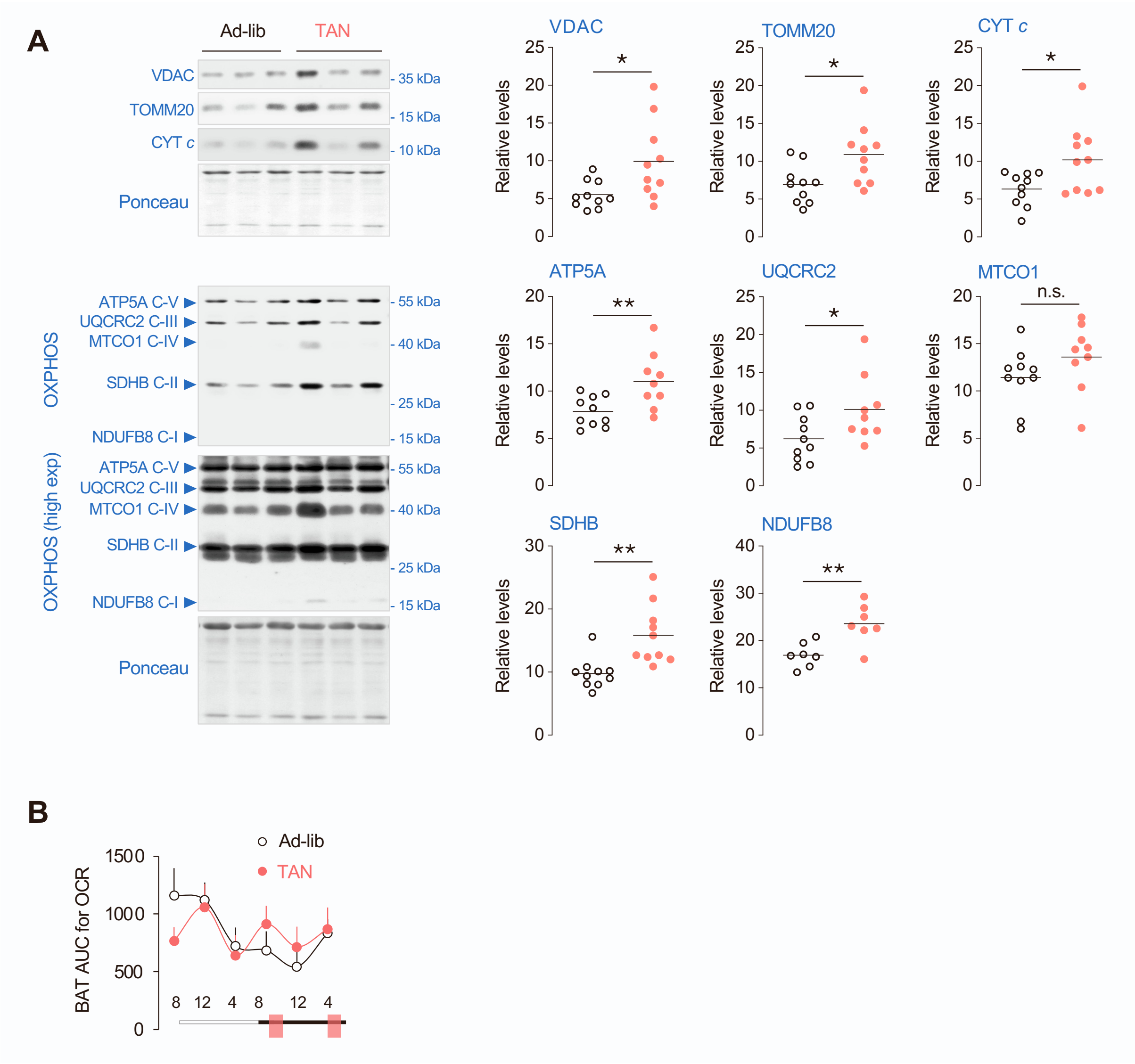

Fig S3 (relates to Fig 3). The impact of TAN feeding on mitochondrial mass and OXPHOS in sWAT and OCR in BAT.

(A) Immunoblots (IB) for indicated mitochondrial markers and OXPHOS components, and their corresponding quantifications in sWAT from C57BL6/J male mice fed ad-lib (n=10) or TAN (n=10) on RD for 5 mo. Ponceau is loading control. Dot plots show individual values (dots) and mean (line). *P < 0.05 and **P<0.01 for two-tailed unpaired Student's t-test.

(B) AUC for OCR in BAT at 6 time-points across 24 h from C57BL6/J male mice fed ad-lib (n=5) or TAN-fed (n=5) on RD for 6 mo. Feeding windows are indicated by *salmon-colored* boxes. The plot across 24h show the mean values (dots) per timepoint and ±SEM.

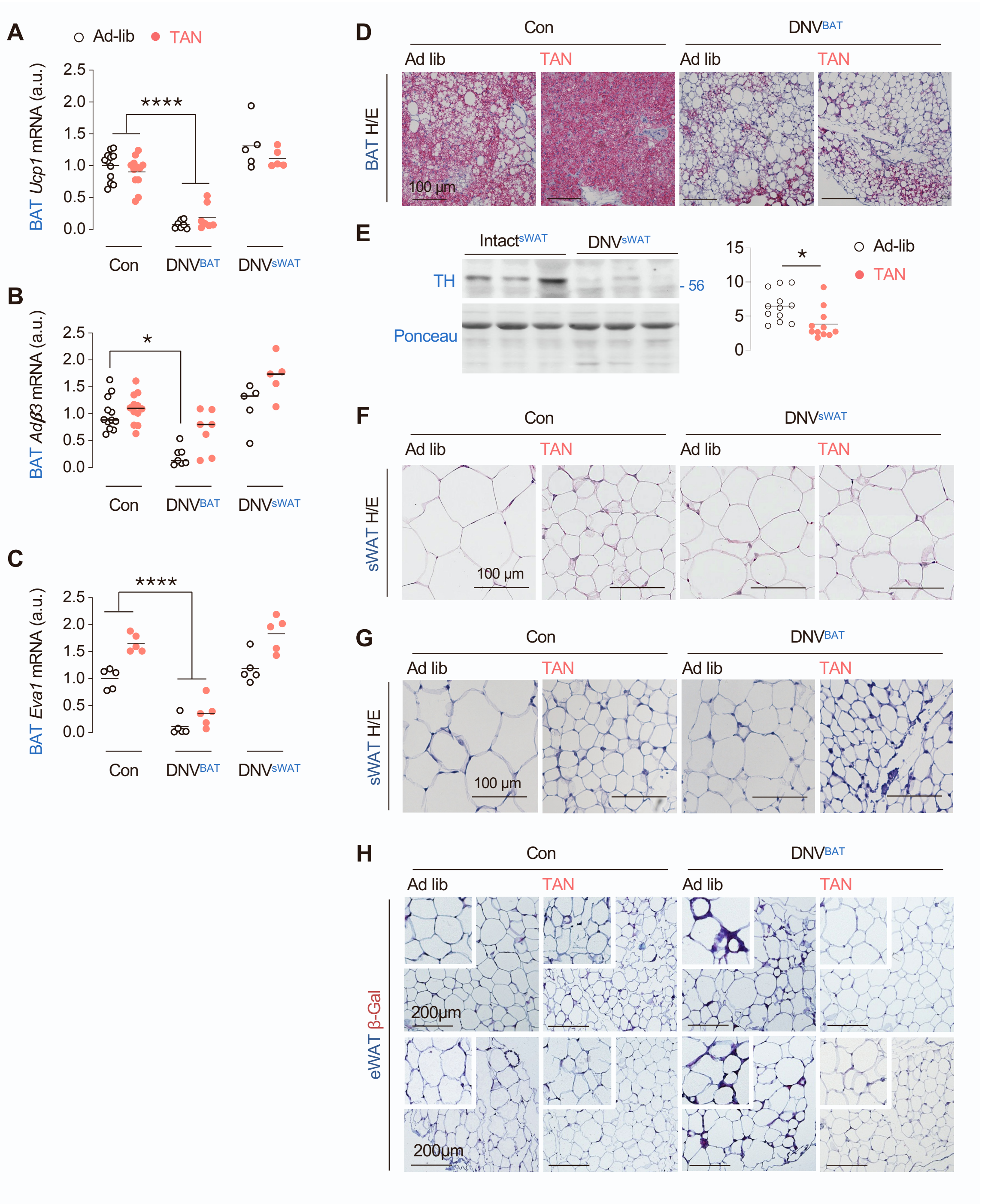

Fig S4 (relates to Fig 4)**. Confirmation of denervations of BAT and sWAT.**

(A-C) qPCR for indicated genes in BAT and their quantifications are shown in innervated control (Con) or denervated sWAT (DNV^{sWAT}) or BAT (DNV^{BAT}) from C57BL6/J male mice fed ad-lib (n=5-12 Con, n=5-7 DNV^{BAT}, n=5 DNV^{sWAT}) or TAN (n=5-12 Con, n=5-7 DNV^{BAT}, n=5 DNV^{sWAT}) on HFD for 3 mo.

(D) Representative H&E staining of BAT from Con and DNV^{BAT} C57BL6/J male mice fed ad-lib or TAN on HFD for 3 mo.

(E) Immunoblots (IB) for Tyrosine hydroxylase (TH) (and corresponding quantifications) in sWAT from Con (n=11) or DNV^{sWAT} (n=11) C57BL6/J male mice fed ad-lib or TAN on HFD for 3 mo.

(F, G) Representative H&E staining of sWAT from Con and DNV^{sWAT} **(F)**, and Con and DNV^{BAT} **(G)** C57BL6/J male mice fed ad-lib or TAN on HFD for 3 mo.

(H) Representative b-galactosidase staining in eWAT from Con or DNV^{BAT} C57BL6/J male mice fed ad-lib or TAN on HFD for 3 mo.

Dot plots show individual values (dots) and mean (line). *P<0.05, ****P<0.0001. Two-way ANOVA and Tukey-corrected.

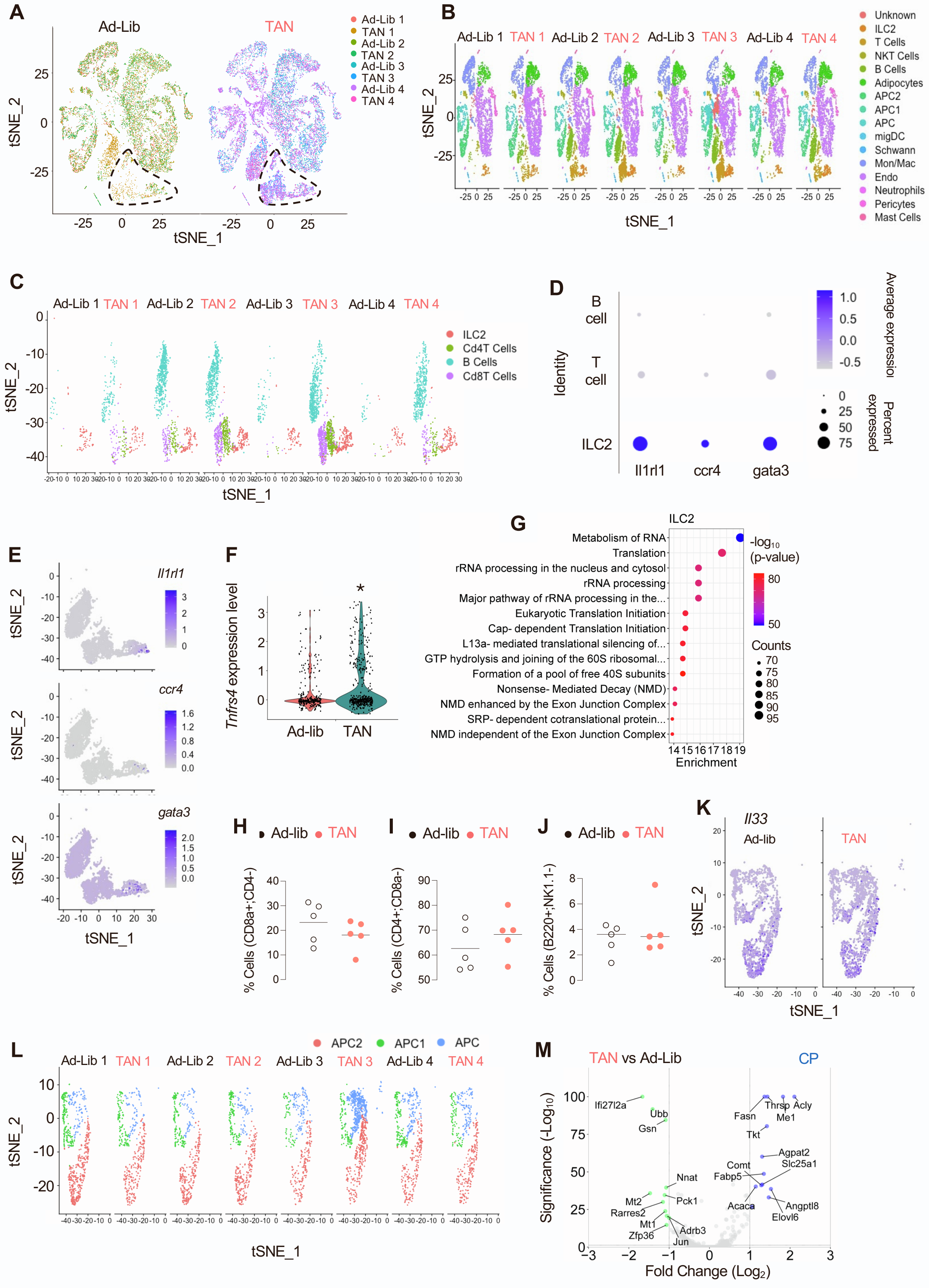
A
Ad-Lib
TAN
Ad-Lib 1
TAN 1
Ad-Lib 2
TAN 2
Ad-Lib 3
TAN 3
Ad-Lib 4
TAN 4
tSNE_1
tSNE_2
B
Unknown
ILC2
T Cells
NKT Cells
B Cells
Adipocytes
APC2
APC1
APC
migDC
Schwann
Mon/Mac
Endo
Neutrophils
Pericytes
Mast Cells
C
ILC2
Cd4T Cells
B Cells
Cd8T Cells
D
B cell
T cell
ILC2
Identity
Il1rl1
ccr4
gata3
Average expression
Percent expressed
E
Il1rl1
ccr4
gata3
F
Tnfrs4 expression level
Ad-lib
TAN
G
ILC2
Metabolism of RNA
Translation
rRNA processing in the nucleus and cytosol
rRNA processing
Major pathway of rRNA processing in the...
Eukaryotic Translation Initiation
Cap- dependent Translation Initiation
L13a- mediated translational silencing of...
GTP hydrolysis and joining of the 60S ribosomal...
Formation of a pool of free 40S subunits
Nonsense- Mediated Decay (NMD)
NMD enhanced by the Exon Junction Complex
SRP- dependent cotranslational protein...
NMD independent of the Exon Junction Complex
Enrichment
-log10 (p-value)
Counts
H
Ad-lib
TAN
% Cells (CD8a+;CD4-)
I
% Cells (CD4+;CD8a-)
J
% Cells (B220+;NK1.1-)
K
Il33
Ad-lib
TAN
L
APC2
APC1
APC
M
TAN vs Ad-Lib
CP
Significance (-Log10)
Fold Change (Log2)
Ifi27l2a
Ubb
Gsn
Nnat
Pck1
Mt2
Rarres2
Mt1
Zfp36
Adrb3
Jun
Fasn
Thrsp
Acly
Me1
Tkt
Agpat2
Slc25a1
Comt
Fabp5
Acaca
Angptl8
Elovl6

Figure S5

Fig. S5 (relates to Fig 5). scRNAseq analyses of sWAT stromal vascular fractions from ad-lib and TAN-fed mice.

(A-B) t-SNE plots showing clustering of cells from each sWAT SVF replicate of C57BL6/J male mice fed ad-lib or TAN for 5 mo on RD, n=4 each group.

(C) t-SNE sub-clustering of immune cells from each sWAT SVF replicate of C57BL6/J male mice fed ad-lib or TAN for 5 mo on RD, n=4 each group.

(D-E) Identity analysis **(D)** and t-SNE graphs **(E)** for expression of indicated ILC2 cell markers. Interleukin 1 receptor-like 1 (*Il1ri1*), C-C Motif Chemokine Receptor 4 (*ccr*4) and Trans-acting T-cell-specific transcription factor GATA-3 (*gata3*). n=4 each group.

(F) Expression of TNF receptor superfamily member 4 (*Tnfrsf4*) in ILC2 population from C57BL6/J male mice fed ad-lib or TAN for 5 mo on RD, n=4 each group.

(G) Bubble plot based on Reactome biological pathways showing the top 14 up-regulated pathways in ILC2 population in Fig 5C. Bubble size represents number of genes/pathway, Y-axis represents the percentage of enrichment and bubble color represents the -$\log_{10}$ P-value (n=4 each group).

(H-J) Quantification for indicated immune cells in sWAT SVF from C57BL6/J male fed ad-lib or TAN on RD for 5 mo (n=5 each group).

(K) t-SNE graphs for APC sub-clustering showing expression of *Il33* in C57BL6/J male mice fed ad-lib or TAN on RD for 5 mo (n=4 each group).

(L) t-SNE graphs for APC sub-clustering for each replicate from C57BL6/J male mice fed ad-lib or TAN on RD for 5 mo (n=4 each group).

(M) Volcano plot showing up- and down-regulated genes in sWAT Committed Preadipocytes (CP) in TAN-fed mice compare to ad-lib mice (n=4).

Dot plots show individual values (dots) and mean (line). n.s.=not significant for two-tailed unpaired Student's *t*-test (H, I, J).

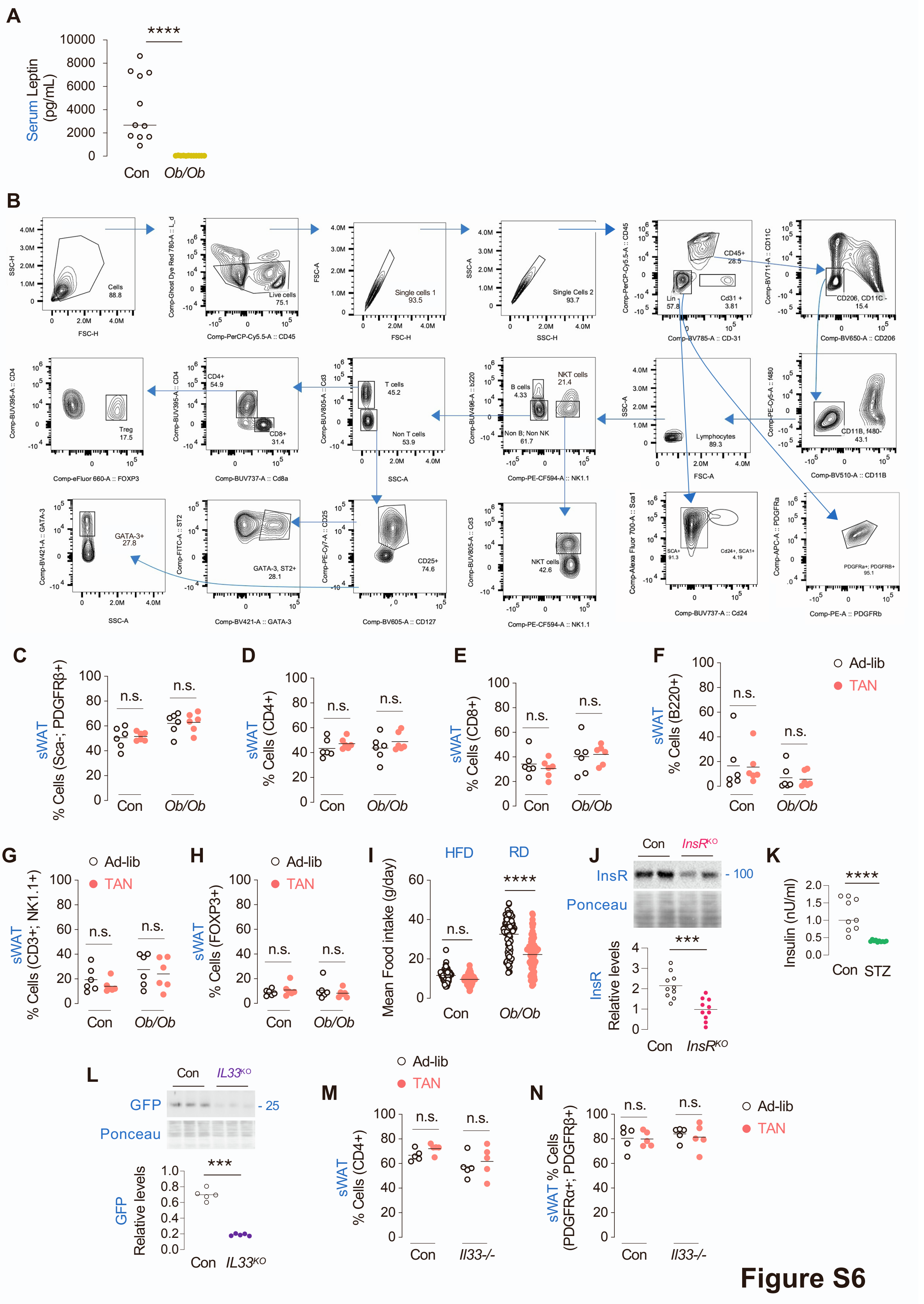
A
Serum Leptin (pg/mL)
****
Con
Ob/Ob
B
Cells 88.8
Live cells 75.1
Single cells 1 93.5
Single Cells 2 93.7
CD45+ 28.5
Lin - 57.8
Cd31 + 3.81
CD206, CD11C - 15.4
Treg 17.5
CD4+ 54.9
CD8+ 31.4
T cells 45.2
Non T cells 53.9
B cells 4.33
NKT cells 21.4
Non B; Non NK 61.7
Lymphocytes 89.3
CD11B, f480- 43.1
GATA-3+ 27.8
GATA-3, ST2+ 28.1
CD25+ 74.6
NKT cells 42.6
SCA+ 91.3
Cd24+, SCA1+ 4.19
PDGFRa+; PDGFRB+ 95.1
C
sWAT % Cells (Sca-; PDGFRβ+)
D
sWAT % Cells (CD4+)
E
sWAT % Cells (CD8+)
F
sWAT % Cells (B220+)
Ad-lib
TAN
n.s.
G
sWAT % Cells (CD3+; NK1.1+)
H
sWAT % Cells (FOXP3+)
I
Mean Food intake (g/day)
HFD
RD
J
Con
InsR^KO
InsR
Ponceau
- 100
InsR Relative levels
***
K
Insulin (nU/ml)
Con
STZ
L
Con
IL33^KO
GFP
Ponceau
- 25
GFP Relative levels
M
sWAT % Cells (CD4+)
Il33-/-
N
sWAT % Cells (PDGFRα+; PDGFRβ+)
Figure S6

Fig S6 (relates to Fig 6). Validations of KO mice models, and characterizing the effect of TAN feeding on immune cells from sWAT SVF of *Ob/Ob* mice.
(A) Serum leptin levels in Con (n=11) and *Ob/Ob* mice (n=12).
(B) Gating strategy to identify different immune cell populations in sWAT SVF.
(C-H) Quantification for progenitors (**C**), $CD4^+$ (**D**), $CD8^+$ (**E**), B220+ cells (**F**), NK1.1+ (**G**) and FOXP3+ Treg (**H**) cells in sWAT SVF from C57BL6/J control fed HFD or *Ob/Ob* fed on RD and subjected to ad-lib or TAN for 3 mo (n=6 each group).
(I) Mean food intake (g/cage/day) in Con (n=11) and *Ob/Ob* mice (n=12) for the duration of the study. Each dot represents food consumed in grams (g) per cage per day.
(J) IB and densitometric quantification for Insulin receptor (InsR) in liver from C57BL6/J male Con and whole-body $InsR^{KO}$ mice (n=10 each group).
(K) Serum insulin levels in C57BL6/J male Con (n=9) and STZ-injected (n=10) mice fed RD for 5 mo.
(L) IB for Green Fluorescence Protein (GFP) and densitometric quantification in liver from C57BL6/J male control or whole-body $Il33^{KO}$ mice fed HFD for 3 mo (n=5 each group).
(M-N) Quantification for CD4+ cells **(M)** and progenitor cells (**N**) in sWAT SVF from C57BL6/J male control or whole-body $Il33^{KO}$ mice fed ad-lib or TAN (n=5 each group).
Dot plots show individual values (dots) and mean (line). n.s.=not significant, ***P<0.001, ****P<0.0001. Two-way ANOVA and Tukey-corrected (C, D, E, F, G, H, I, M, N), and two-tailed unpaired Student's *t*-test (A, J, K, L).

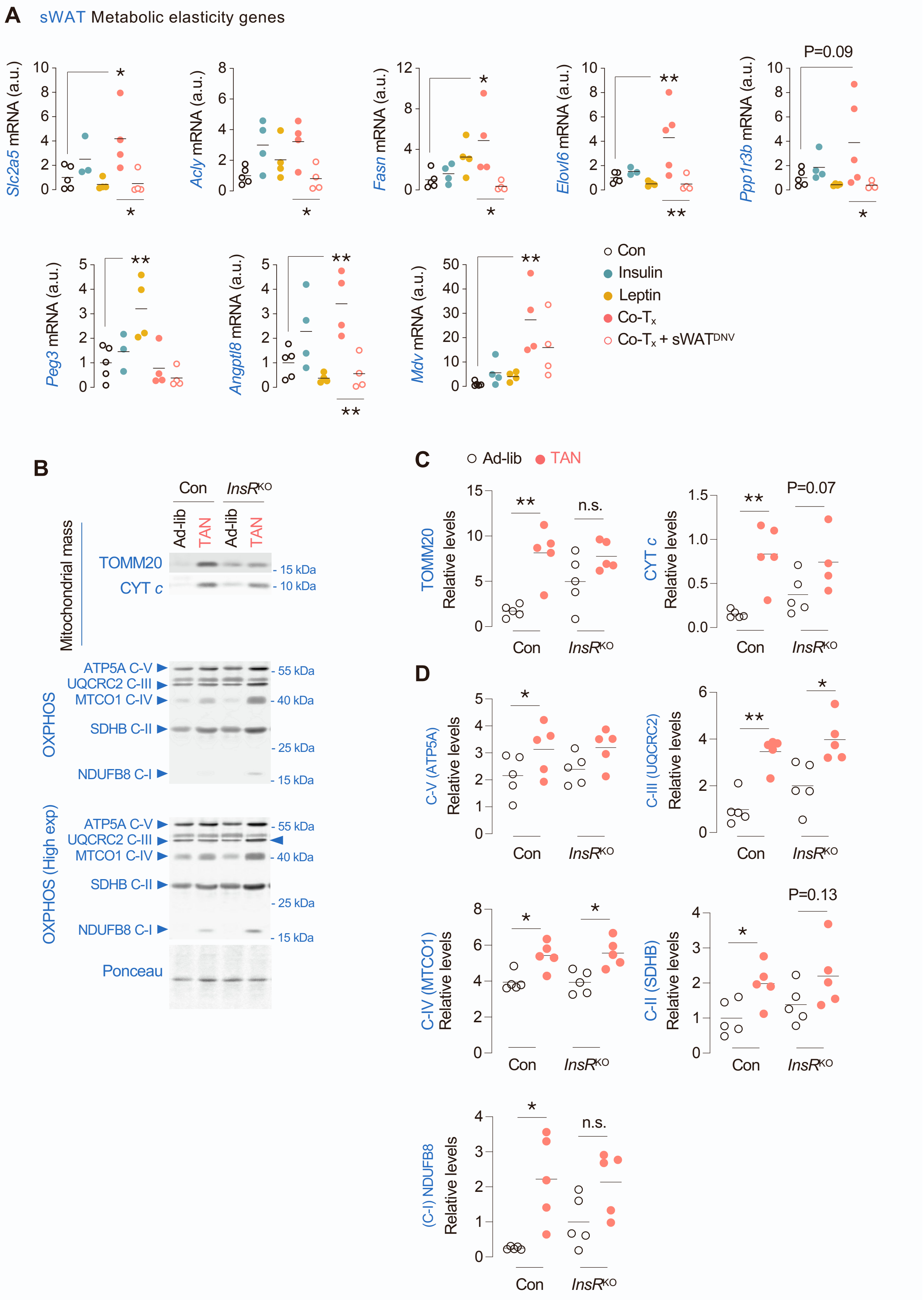
A sWAT Metabolic elasticity genes
Slc2a5 mRNA (a.u.)
Acly mRNA (a.u.)
Fasn mRNA (a.u.)
Elovl6 mRNA (a.u.)
Ppp1r3b mRNA (a.u.)
P=0.09
Peg3 mRNA (a.u.)
Angptl8 mRNA (a.u.)
Mdv mRNA (a.u.)
Con
Insulin
Leptin
Co-Tx
Co-Tx + sWATDNV
B
Con
InsRKO
Ad-lib
TAN
Ad-lib
TAN
Mitochondrial mass
TOMM20
CYT c
15 kDa
10 kDa
OXPHOS
ATP5A C-V
UQCRC2 C-III
MTCO1 C-IV
SDHB C-II
NDUFB8 C-I
55 kDa
40 kDa
25 kDa
15 kDa
OXPHOS (High exp)
Ponceau
C
Ad-lib
TAN
TOMM20 Relative levels
CYT c Relative levels
n.s.
P=0.07
D
C-V (ATP5A) Relative levels
C-III (UQCRC2) Relative levels
C-IV (MTCO1) Relative levels
C-II (SDHB) Relative levels
P=0.13
(C-I) NDUFB8 Relative levels

Figure S7

Fig. S7 (relates to Fig 7). Impact of injections of insulin and leptin on metabolic flexibility genes in sWAT and the effect of loss of insulin receptor signaling on benefits of TAN feeding.
(A) qPCR for indicated metabolic elasticity genes in sWAT from C57BL6/J male mice fed ad-lib on RD and injected with the indicated hormones for 1.5 mo. Con (vehicle-injected, n=5), insulin (n=4), leptin (n=4), Co-T_x (insulin and leptin-injected, n=4-5) and DNV^{sWAT} co-T_x (n=4).
(B-D) Representative IB and quantifications for indicated mitochondrial markers and OXPHOS components in sWAT from Con or $InsR^{KO}$ male mice fed ad-lib (n=5 Con, n=5 $InsR^{KO}$) or TAN (n=5 Con, n=5 $InsR^{KO}$) for 5 mo. Ponceau is loading control.
Dot plots show individual values (dots) and mean (line). n.s.=not significant, *P<0.05, **P<0.01. Two-way ANOVA and Tukey-corrected.

Supplemental Video Title and Legends

Supplemental Video 1. 3D visualization of sWAT from a representative ad-lib-fed mouse. Adipocyte volume (0-100,000 μm3) is color-coded. Tissue autofluorescence is grey scale.
Supplemental Video 2. 3D visualization of sWAT from a representative, TAN-fed mouse. Adipocyte volume (0-100,000 μm3) is color-coded. Tissue autofluorescence is grey scale.
Supplemental Video 3. 3D visualization of CD31 staining (red) in sWAT from a representative ad-lib-fed.
Supplemental Video 4. 3D visualization of CD31 staining (red) in sWAT from a representative TAN-fed.

Supplemental Table Title and Legends

Supplemental Table 1: Pathway enrichment analysis using BIOCARTA for top-100 upregulated genes in sWAT of TAN versus ad-lib-fed mice on RD for 6 mo.
Supplemental Table 2: Limma DEG analysis for all 6 time points in sWAT of TAN versus ad-lib-fed mice on RD for 6 mo.
Supplemental Table 3: Gene_FPKM and Candidate_gene_FPKM for all 6 time points in sWAT of TAN versus ad-lib-fed mice on RD for 6 mo.
Supplemental Table 4: Metabolic Plasticity FPKM Correlation Enrichment network for all 6 time points in sWAT of TAN versus ad-lib-fed mice on RD for 6 mo.

Supplemental Table 5: Primers for real-time PCR analysis related to STAR Methods

Primers for real-time PCR analysis related to STAR Methods

GENE	PROTEIN	PRIMERS
Adrb3	Adrenergic receptor, beta 3	(f) 5'- ggcaacctgctggtaatcat-3'<br>(r) 5'- tccactgacgtccacagttc-3'
Eva1a	Eva-1 homolog A, regulator of programmed cell death	(f) 5'- ccacttctcctgagtttacagc-3'<br>(r) 5'- gcattttaaccgaacatctgtcc-3'
Tbp	TATA box binding protein	(f) 5'- gaagctgcggtacaattccag-3'<br>(r) 5'- ccccttgtacccttcaccaat-3'
Slc2a5	Solute carrier family 2 (facilitated glucose transporter), member 5	(f) 5'- cgaaaaacctacgaggggct-3'<br>(r) 5'- ctggccagccatcctcattt-3'
Acly	ATP citrate lyase	(f) 5'- cgtcgaggaaagcttgg-3'<br>(r) 5'- tgagaaagttcttgaggaagc-3'
Fasn	Fatty acid synthase	(f) 5'- ctgactcggctactgacacg-3'<br>(r) 5'- tgagctgggttagggtagga-3'
Elovl6	ELOVL fatty acid elongase 6	(f) 5'- cgtagcgactccgaagatcagcc-3'<br>(r) 5'- agcgtacagcgcagaaaacagga -3'
Ppp1r3b	Protein phosphatase 1, regulatory subunit 3B	(f) 5'- actgtgtgctgaaggataagg-3'<br>(r) 5'- cacatactgacaagggaaatct-3'
Peg3	Paternally Expressed 3	(f) 5'- agtccagcttgccgaagat-3'<br>(r) 5'- ctcaggcatgggtttgagac-3'
Lep	Leptin	(f) 5'- tctccgagacctcctccatct-3'<br>(r) 5'- ttccaggacgccatccag-3'
Angptl8	Angiopoietin-like 8	(f) 5'- ctcaatggcgtgtacagagc-3'<br>(r) 5'- tcgaaggtgtaaagcgtcct -3'
Mvd	Mevalonate (diphospho) decarboxylase	(f) 5'- agtccagcttgccgaagat-3'<br>(r) 5'- ctcaggcatgggtttgagac-3'
