# Supplementary material for: Insulin and leptin oscillations license food-entrained browning and metabolic flexibility
Source: Cell Rep. Author manuscript; Available in PMC 2024 Nov 14. (PMC11562929; doi:10.1016/j.celrep.2024.114390)
Supplement: 2 [file NIHMS2011856-supplement-2.pdf]

Cell Reports

Article

Insulin and leptin oscillations license food-entrained browning and metabolic flexibility

Graphical abstract

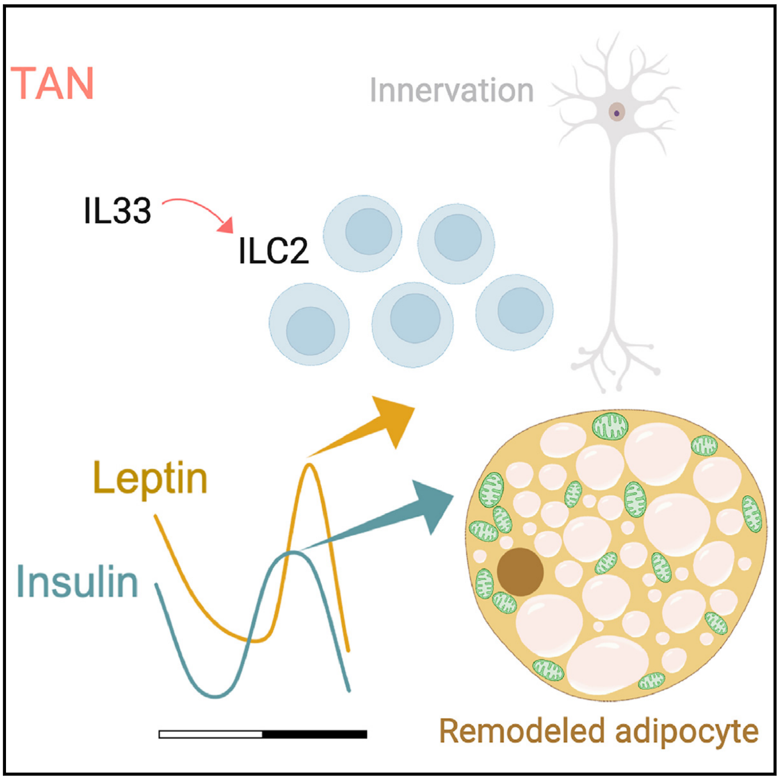

Highlights

- Two meals per period entrains insulin and leptin oscillations
- Insulin and leptin oscillations drive the cellular, immune, and metabolic remodeling of sWAT
- sWAT remodeling leads to metabolic flexibility and inflammation resolution in eWAT
- Recreating insulin and leptin oscillations pharmacologically recapitulates sWAT remodeling

Authors

Pamela Mattar, Andressa Reginato, Christian Lavados, ..., Gary Schwartz, Prashant Rajbhandari, Rajat Singh

Correspondence

rajatsingh@mednet.ucla.edu

In brief

Mattar et al. show that two meals per period entrains insulin and leptin oscillations to drive subcutaneous fat browning and metabolic flexibility. Targeting insulin or leptin signaling or ILC2 cell recruitment or tissue denervation each blocks subcutaneous fat browning in food-entrained mice, while reconstituting endocrine oscillations facilitates adipose browning.

Mattar et al., 2024, Cell Reports *43*, 114390
July 23, 2024 © 2024 The Author(s). Published by Elsevier Inc.
https://doi.org/10.1016/j.celrep.2024.114390

CellPress

Cell Reports

CellPress OPEN ACCESS

Article

Insulin and leptin oscillations license food-entrained browning and metabolic flexibility

Pamela Mattar,[1,9] Andressa Reginato,[2,9] Christian Lavados,[2] Debajyoti Das,[1] Manu Kalyani,[2] Nuria Martinez-Lopez,[1,8] Mridul Sharma,[1] Grethe Skovbjerg,[3] Jacob Lercke Skytte,[3] Urmas Roostalu,[3] Rajasekaran Subbarayan,[4] Elodie Picarda,[5] Xingxing Zang,[5] Jinghang Zhang,[5] Chandan Guha,[4] Gary Schwartz,[6] Prashant Rajbhandari,[7] and Rajat Singh[1,2,6,8,10,*]

[1]Department of Medicine, Division of Digestive Diseases, University of Los Angeles, Los Angeles, CA, USA
[2]Department of Developmental and Molecular Biology, Albert Einstein College of Medicine, Bronx, NY, USA
[3]Gubra ApS, Hørsholm, Denmark
[4]Radiation Oncology, Albert Einstein College of Medicine, Bronx, NY, USA
[5]Department of Microbiology and Immunology, Albert Einstein College of Medicine, Bronx, NY, USA
[6]Department of Medicine, Albert Einstein College of Medicine, Bronx, NY, USA
[7]Department of Medicine, Diabetes, Obesity, and Metabolism Institute, Icahn School of Medicine at Mount Sinai, New York, NY, USA
[8]Comprehensive Liver Research Center at UCLA, University of Los Angeles, Los Angeles, CA, USA
[9]These authors contributed equally
[10]Lead contact
*Correspondence: rajatsingh@mednet.ucla.edu

https://doi.org/10.1016/j.celrep.2024.114390

SUMMARY

Timed feeding drives adipose browning, although the integrative mechanisms for the same remain unclear. Here, we show that twice-a-night (TAN) feeding generates biphasic oscillations of circulating insulin and leptin, representing their entrainment by timed feeding. Insulin and leptin surges lead to marked cellular, functional, and metabolic remodeling of subcutaneous white adipose tissue (sWAT), resulting in increased energy expenditure. Single-cell RNA-sequencing (scRNA-seq) analyses and flow cytometry demonstrate a role for insulin and leptin surges in innate lymphoid type 2 (ILC2) cell recruitment and sWAT browning, since sWAT depot denervation or loss of leptin or insulin receptor signaling or ILC2 recruitment each dampens TAN feeding-induced sWAT remodeling and energy expenditure. Consistently, recreating insulin and leptin oscillations via once-a-day timed co-injections is sufficient to favorably remodel innervated sWAT. Innervation is necessary for sWAT remodeling, since denervation of sWAT, but not brown adipose tissue (BAT), blocks TAN-induced sWAT remodeling and resolution of inflammation. In sum, reorganization of nutrient-sensitive pathways remodels sWAT and drives the metabolic benefits of timed feeding.

INTRODUCTION

Restricting calories[1] or feeding times[2,3] remodels the subcutaneous white adipose tissue (sWAT). White fat plasticity drives energy expenditure,[4] with multiple studies documenting associations of thermogenic human subcutaneous fat with healthspan extension.[5,6] However, how dietary interventions facilitate white fat plasticity remains unclear. In mice, predominant thermogenic adipocytes include the classical interscapular brown adipocytes and inducible beige/brite cells interspersed within sWAT. Although intrascapular brown adipose tissue (BAT) displays greater thermogenic activity by weight, it is sWAT that acutely and visibly remodels with cold or fasting. The relative contribution of thermogenic BAT versus remodeled sWAT to energy expenditure in response to dietary interventions remains understudied.

Here, we show that feeding within two windows in the nocturnal cycle (twice a night [TAN]) drives sWAT remodeling and metabolic flexibility and lowers visceral fat inflammation. Depot-specific denervations of BAT or sWAT reveal that benefits of TAN feeding are driven by sWAT alone. Interestingly, sustained TAN feeding entrains insulin and leptin oscillations, reflected by feeding-driven surges in their levels, which remain unaffected in mice housed in darkness. We propose a mechanistic framework wherein insulin and leptin oscillations couple innate lymphoid type 2 (ILC2) cells to sWAT browning. ILC2 cell recruitment and sWAT browning each requires neuroendocrine input, since inactivating insulin or leptin signaling or denervating sWAT dampens ILC2 recruitment and depot energy expenditure in TAN-fed mice. Consistently, recreating leptin and insulin oscillations in *ad libitum*-fed (ad-lib) mice via timed once-daily co-injections is sufficient to induce sWAT browning—supporting the idea that food-entrained endocrine oscillations drive sWAT remodeling and metabolic flexibility.

RESULTS

TAN feeding rewires metabolic programs in sWAT

To determine how intermittent fasting impacts sWAT function, we established a TAN feeding intervention as an extension of our previously described "twice-a-day" (TAD) feeding, which

© 2024 The Author(s). Published by Elsevier Inc.
This is an open access article under the CC BY license (http://creativecommons.org/licenses/by/4.0/).

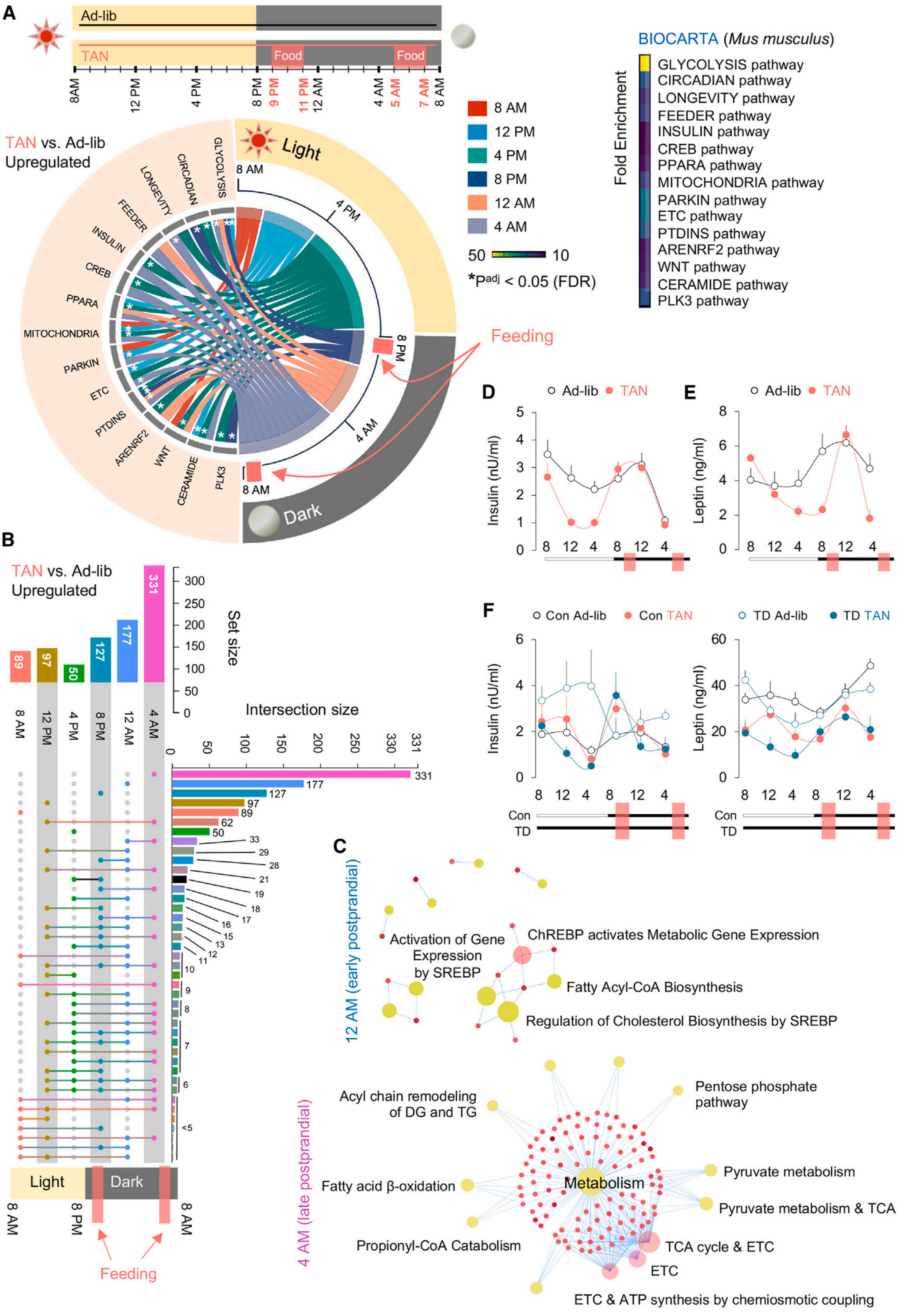

(legend on next page)

provides diverse systemic benefits despite diurnal feeding.[3] To this purpose, C57BL/6J male mice were fed twice in their nocturnal cycle (free access to water) and compared to age-matched ad-lib controls (Cons). TAN mice were acclimatized to feeding between 9:00 and 11:00 p.m. (first feeding window) and 5:00 and 7:00 a.m. (second feeding window) in an 8:00 p.m. to 8:00 a.m. dark cycle (Figure 1A). These nocturnal time frames were chosen because these mirrored the diurnal feeding times of our TAD feeding studies[3] and because TAN feeding in mice broadly reflects morning and evening meals in humans. As with TAD feeding, a 10–14 day acclimatization period allows adaptation to feeding in these two 2 h windows, although TAN-fed mice on regular chow diet (RD) or high-fat diet (HFD) each displayed modestly reduced food intake compared to corresponding ad-lib Cons (Figures S1A and S1B). Hence, the beneficial outcomes of TAN feeding are likely due to partitioned feeding and mildly reduced caloric intake. We also observed that TAN-fed mice displayed slightly increased food intake in the second feeding window (5:00–7:00 a.m.) compared with the first feeding window (9:00–11:00 p.m.) (Figures S1C).

Since caloric restriction (CR)[1] stimulates sWAT browning, we asked if and how TAN feeding would lead to sWAT browning. To test this, we performed transcriptomics in sWAT from ad-lib and TAN mice at six circadian time points. Analyses of differentially expressed genes (DEGs), and filtering for low expression (<10 counts), revealed 5,733 DEGs in the sWAT of TAN mice compared to ad-lib Con. We used two-way exclusion to improve the yield of statistically significant genes without accumulating type I errors and sorted for DEGs by $\log_{FC}$ (>1) ($p < 0.05$) (Table S1). Employing BIOCARTA to identify period-wide alterations of pathways by TAN, we observed marked enrichment of "circadian" genes between 4:00 and 8:00 p.m., i.e., the time point preceding the first nocturnal feeding and the first feeding period per se (Figure 1A). By contrast, the nutrient-driven "glycolysis" genes were enriched during and after each feeding interval (Figure 1A), while the "feeder" pathway genes were enriched postprandially from 12:00 p.m. to 8:00 a.m. (Figure 1A). Interestingly, TAN feeding enriched longevity-related genes at time points preceding each feeding interval (4:00–8:00 p.m.) and extending into the second feeding period (4:00–8:00 a.m.) (Figure 1A). Strikingly, the greatest associations with diverse candidate pathways were observed at intervals preceding the two feeding time points, indicated by the number of connecting threads (Figure 1A). Since TAN feeding causes enrichment of nutrient-regulated insulin/phosphoinositide signaling (PTDINS pathway)[7] in sWAT, we suspected that nutrient-related signaling is involved in the sWAT remodeling and metabolic benefits of TAN feeding. Indeed, TAN feeding blunted body weight gain and fat accrual, improved glucose clearance, and notably reduced epididymal fat (eWAT) inflammation (decreased F4/80 positivity) in HFD-fed mice (Figures S2A–S2E).

An UpSet plot for period-wide sWAT transcriptomes revealed time points when specific gene sets are perturbed by TAN feeding (Figure 1B). The greatest enrichment of distinct DEGs occurred after the first feeding period, with 177 and 331 DEGs ($\log_{FC} > 1$, $p < 0.05$) enriched at 12:00 a.m. or 4:00 a.m., respectively. By contrast, the second feeding period only modestly affected the sWAT transcriptome, with 89 and 50 DEGs (n = 50; $\log_{FC} > 1$, $p < 0.05$) enriched at 8:00 a.m. and 4:00 p.m., respectively. In contrast to both feeding periods, fasting phases (e.g., 4:00 a.m. and 12:00 p.m.) were characterized by similar/overlapping DEGs (n = 62) compared to only 10 overlapping genes between two postfeed time points, e.g., 8:00 a.m. and 12:00 a.m. Thus, the first nocturnal feeding appears to exert the greatest impact on the sWAT transcriptome (Figure 1B). This does not appear to be due to increased first-interval feeding, since we observed slightly higher caloric intake in the second feeding period (Figures S1C), but may possibly be due to circadian factors determining response to meals, in particular since rodents are intrinsically hardwired to consume the bulk of their calories within the first few hours of the dark cycle.

TAN feeding segregates anabolic and catabolic networks in sWAT

To determine if TAN feeding leads to metabolic adaptations in sWAT, we performed gene-set enrichment analyses (GSEAs) for significantly upregulated DEGs ($\log_{FC} > 1$, $p < 0.05$) using the Reactome database to identify genetic signatures of metabolic adaptations in sWAT. This was followed by functional enrichment network analyses at two distinct time points after the first feeding window, i.e., 12:00 a.m. (early postprandial) and 4:00 a.m. (late postprandial), which revealed the segregation of anabolic versus catabolic pathways in response to feeding and fasting, respectively (Table S2). For example, expression of glucose-responsive ChREBP-driven anabolic genes and biosynthesis of cholesterol and fatty acyl co-enzyme A (CoA) occurred in the early postprandial period (12:00 a.m.), while the late postprandial period (4:00 a.m.) was characterized by enrichment of catabolic networks, e.g., β-oxidation, tricarboxylic acid (TCA) cycle, and electron transport chain (ETC) (Figure 1C). These analyses suggest that repetitive cycles of feeding and fasting rewire metabolic

Figure 1. TAN feeding entrains insulin and leptin oscillations and rewires metabolic programs in sWAT

(A) Scheme depicting the twice-a-night (TAN) feeding intervention. Bulk RNA-seq analysis was done at the indicated time points in sWAT of C57BL/6J male mice fed *ad libitum* (ad-lib) or TAN for 6 months (n = 5). Period-wide pathway enrichment (BIOCARTA) for the top 100 upregulated genes and a chord diagram for significantly enriched pathways as a function of time are shown. Fold enrichment is represented as a heatmap, and *FDR (false discovery rate) < 0.05.

(B) UpSet plot of samples in (A) depicts intersection sets for significantly enriched genes across the six time points. Each connection represents a distinct combination between six (Zeitgeber) ZT time points and the number of overlapping gene sets.

(C) Enrichment network using the Reactome database for significantly upregulated genes in TAN-fed mice at early postprandial (12:00 a.m.) and late postprandial (4:00 a.m.) time points. Node size represents the enrichment score. Node color represents the significance. Salmon, FDR < 0.05; lemon, $p < 0.05$. The network edge represents the betweenness for genes associated with representative pathways.

(D–F) Serum insulin (D) and leptin (E) at six time points from ad-lib or TAN-fed mice housed at ambient temperature in a 12 h/12 h light/dark cycle (D and E) and in a 24 h dark cycle (F), i.e., total darkness (TD). Feeding windows are indicated by salmon-colored boxes; n = 4–10. Every plot across 24 h shows the mean value (dots) per time point ± SEM. See also Figure S1.

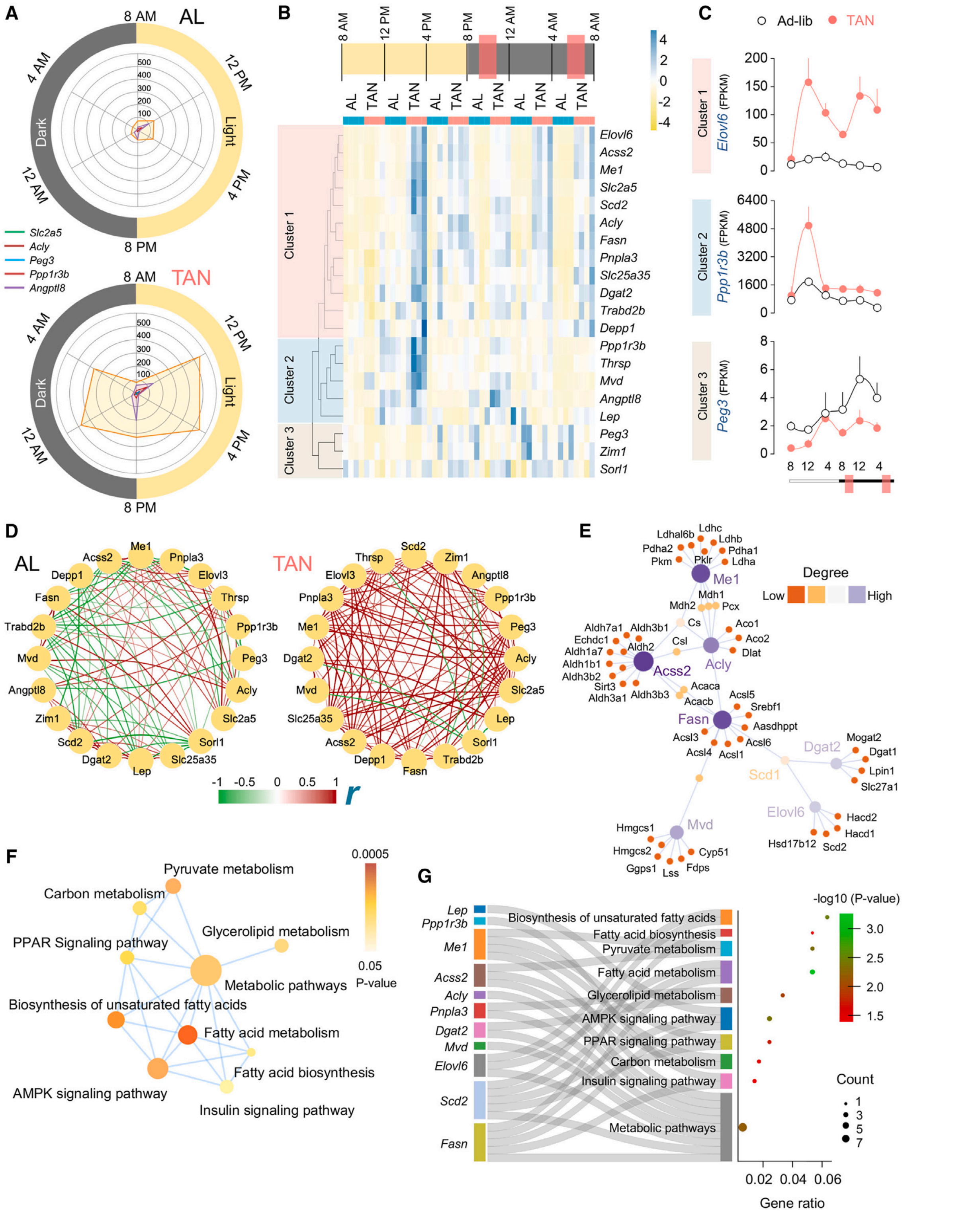

Figure 2. TAN feeding associates with improved metabolic flexibility in sWAT

(A) Bulk RNA-seq of sWAT at six ZT time points from ad-lib and TAN-fed mice indicated in Figure 1A ($n = 4$). Radar charts for period-wide oscillations in the expression of the top 5 metabolic elasticity genes in sWAT from ad-lib and TAN-fed mice are shown. Circle diameter denotes FPKM (fragments per kilobase of transcript per million mapped reads) values.

(legend continued on next page)

circuits such that anabolic and catabolic networks are reorganized within feeding and fasting windows, respectively.

TAN feeding entrains insulin and leptin oscillations

Since TAN feeding segregates anabolic and catabolic networks in sWAT, we asked if and how TAN feeding shapes the circadian oscillations of the hormones insulin and leptin, which are typically perturbed by nutrient availability. Period-wide analyses showed oscillations of circulating insulin and leptin in response to TAN feeding—with well-defined peaks coinciding with 9:00–11:00 p.m. (first feeding) and 5:00–7:00 a.m. (second feeding) windows (Figures 1D and 1E). Feeding-driven insulin surges were also noted in cohorts of mice subjected to 40% CR (Figure S2F) or TAD feeding,[3] which consume their meals within restricted feeding windows. At least in TAN mice, insulin and leptin oscillations occurred independent of changes in circadian corticosterone levels (Figure S2G). Consistently, TAN feeding-induced insulin and leptin oscillations correlated with similar oscillations in insulin- and leptin-responsive genes (Figures S2H and S2I; Table S3). For example, cluster 1 genes (insulin signaling-related *Pik3cg* and *Grb2*) exhibited period-wide correlations with insulin surges, while cluster 2 (*Csnk2a2* and *Sos1*) and cluster 4 (*Slc2a4* and *Mapk3*) genes oscillated in response to either the nocturnal or the diurnal insulin surge, respectively (Figure S2H). Similar correlations between oscillations of leptin-responsive gene clusters (Figure S2I) point to a broad and adaptive reprogramming of metabolic pathways in sWAT in response to TAN feeding.

Insulin and leptin oscillations are not affected by housing in darkness

Interestingly, insulin and leptin oscillations appear to be primarily feeding driven (reflecting their entrainment by food), since subjecting TAN-fed mice to an established intervention that disrupts the central clock, i.e., housing in a 24 h dark cycle (total darkness [TD]) failed to affect the amplitude or phase of insulin and leptin oscillations in response to TAN feeding (Figures 1D and 1E). Since TAN-fed mice retain their endocrine oscillations (Figure 1F) and resist eWAT inflammation despite being housed in TD (Figure S2J), we hypothesized that food-entrained insulin and leptin oscillations contribute, at least in part, to the benefits of TAN feeding.

TAN feeding perturbs metabolic flexibility genes in sWAT

Given the marked remodeling of the sWAT transcriptome with TAN feeding, we asked if TAN feeding leads to metabolic flexibility within sWAT, i.e., the ability to utilize carbohydrates or lipids in response to physiological cues.[8] Accordingly, we employed the gene elasticity score (GElaS) algorithm/scoring system to determine how TAN feeding impacts the period-wide oscillations in sWAT of 20 defined metabolic "elasticity genes."[9] Radar chart representation depicted significant increases in expression and oscillations (including induction in the dark cycle) of the top 5 "elastic" genes in sWAT of TAN mice (Figure 2A), while Euclidean clustering of the 20 elastic genes resulted in three distinct clusters in TAN mice based on their oscillation patterns (Figure 2B). Cluster 1 genes (12 genes, e.g., *Elovl6*) are uniquely upregulated by TAN feeding with a biphasic oscillation pattern, i.e., two feeding-related peaks (12:00 p.m. and 12:00 a.m.) across period (Figures 2B and 2C). Cluster 2 genes (5 genes, e.g., *Ppp1r3b*) show a single diurnal 12:00 p.m. surge after the second feeding (Figures 2B and 2C). By contrast, cluster 3 (3 genes, e.g., *Peg3*) is not responsive to feeding (Figures 2B and 2C; Table S4). Notably, the majority of cluster 1 and 2 elastic genes shows diurnal oscillations in the prolonged fasting phase (Figure 2B), although the significance of the diurnal oscillations remains unclear.

Metabolic elasticity entails coordinated gene oscillations.[9] Consistently, TAN feeding led to a remarkable enrichment of positive correlations between the elastic genes at 12:00 p.m. compared to ad-lib (Figure 2D), indicating concerted molecular events likely representing metabolic flexibility. Consistently, protein-protein interaction network analysis identified hub genes *Fasn*, *Scd1*, *Mvd*, *Me1*, *Acly*, *Acss2*, *Dgat2*, and *Elovl6* (Figure 2E) and their functional submodules and molecular interactors within the top 20 elastic genes, emphasizing coordinated gene networks in sWAT of TAN-fed mice. Furthermore, GSEAs identified functionally enriched pathways and their associations between specific elastic genes and enriched annotations (Figures 2F and 2G)—indicating that "fatty acid metabolism," "insulin signaling," and "pyruvate metabolism" pathways are perturbed in sWAT by TAN feeding. Hence, TAN feeding compartmentalizes anabolic and catabolic pathways, likely reflecting improved metabolic flexibility in sWAT.

TAN feeding remodels sWAT

Given the marked reprogramming of the sWAT transcriptome, we asked if and how TAN feeding remodels sWAT and its function. Consistent with decreased sWAT weight in HFD-fed TAN mice (Figure 3A), 3D analyses of adipocyte volume showed reduced mean sWAT adipocyte size with increased frequency of smaller adipocytes (blue) in TAN mice compared to greater

(B) Period-wide cluster map of the top 20 metabolic elasticity genes in sWAT of ad-lib or TAN-fed mice (n = 4). Z-score-normalized values are plotted and implemented for hierarchical clustering. Sky blue denotes upregulation, and lemon yellow denotes downregulation.

(C) Period-wide oscillations in gene expression determined by qPCR for representative genes in each cluster. The x axis denotes time. The y axis is FPKM value.

(D) Correlation coefficient network diagrams of 20 metabolic elasticity genes from sWAT of ad-lib or TAN-fed mice at 12:00 p.m. Nodes represent individual genes. The edge denotes the number of significant positive and negative correlations. Edge thickness signifies Pearson's correlation coefficient values. Red and green indicate positive or inverse correlations in gene expression.

(E) Protein-protein interaction networks (bipartite) generated for metabolic elasticity gene sets using STRING. The subnetwork is selected on confidence score (>900) threshold. Degree and betweenness are denoted by node size and color.

(F) Gene set enrichment analysis for metabolic elasticity genes at 12:00 p.m. using the KEGG database. Node size represents the enrichment score, while node color signifies the p value (<0.05) of the functional enrichment network.

(G) Sankey bubble chart representation of metabolic elasticity genes and their associated enriched pathways. A corresponding bubble chart for pathway enrichment depicting gene ratio (x axis), $-\log_{10}(p)$ (bubble color), and gene count (bubble size) is shown. Feeding windows are indicated by salmon-colored boxes.

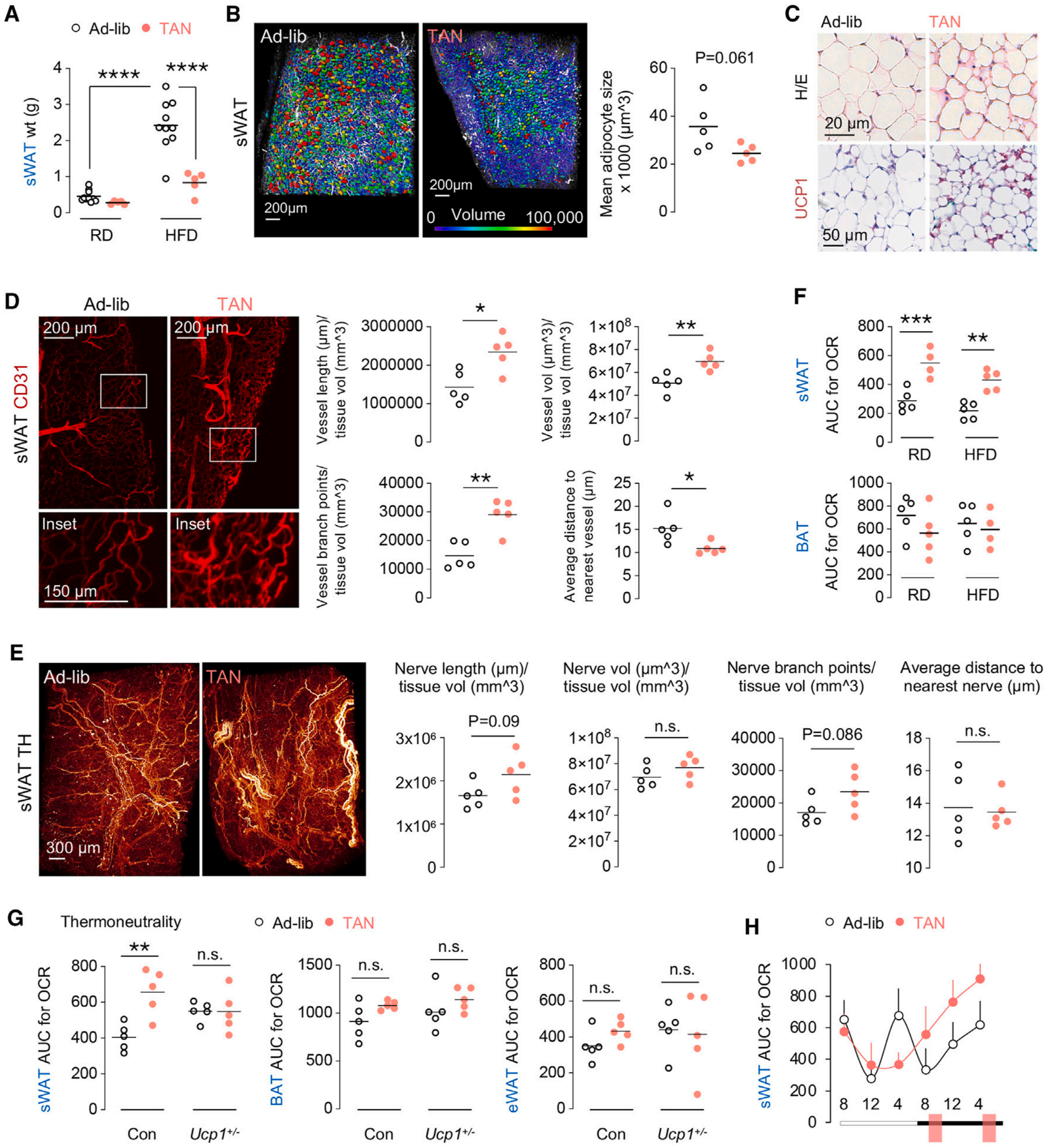

Figure 3. TAN feeding leads to cellular and functional remodeling of sWAT

(A) sWAT weight (wt) of regular chow diet (RD)- or high-fat diet (HFD)-fed C57BL/6J male mice subjected to ad-lib (*n* = 10) or TAN feeding (*n* = 5) for 6 months.

(B) Representative images of adipocytes generated via light-sheet microscopy and 3D reconstruction of whole sWAT (inguinal-dorsal) from C57BL/6J male mice subjected to ad-lib or TAN feeding on RD for 5 months (*n* = 5). Adipocyte volumes are represented in color scale (μm^3) with larger adipocytes in red and smaller adipocytes in purple. Graph shows the mean adipocyte size. See Videos S1 (ad-lib) and S2 (TAN) for 3D constructions of sWAT from ad-lib and TAN-fed mice.

(C) Representative H&E and immunostaining of sWAT from C57BL/6J male mice subjected to ad-lib or TAN feeding for 6 months showing adipocyte size (top) and UCP1 positivity (bottom).

(D) Representative images and quantification of CD31-positive blood vessels generated via light-sheet microscopy of whole sWAT (inguinal-dorsal) from C57BL/6J male mice subjected to ad-lib or TAN feeding on RD for 5 months. White rectangles are magnified in insets below. See Videos S3 (ad-lib) and S4 (TAN) for 3D constructions of sWAT from ad-lib and TAN-fed mice (*n* = 5).

(E) Representative images and quantification of tyrosine hydroxylase (TH)-positive nerves generated via light-sheet microscopy of whole sWAT (inguinal-dorsal) from C57BL/6J male mice fed ad-lib or TAN on RD for 6 months (*n* = 5).

(F) Area under the curve (AUC) for oxygen consumption rate (OCR) in sWAT and BAT of C57BL/6J male mice subjected to ad-lib (RD *n* = 5, HFD *n* = 5) or TAN (RD *n* = 4, HFD *n* = 5) feeding on RD or HFD for 6 months (n = 4–5).

(legend continued on next page)

frequency of mid-sized (green) and larger (red) adipocytes in ad-lib Cons (Figure 3B). Reduction in adipocyte size was associated with increased multilocularity, UCP1 positivity (Figure 3C), and greater vascular density (CD31 positivity) reflected by increases in vessel length, volume, and branchpoint, with reduced average distance to nearest vessel (Figure 3D). In addition, tyrosine hydroxylase (TH) staining, marking tissue innervation, revealed a trend toward increased sWAT innervation in TAN mice (Figure 3E). Remodeling of sWAT correlated with increases in oxygen consumption rate (OCR) (Figure 3F) and levels of the mitochondrial markers VDAC, TOMM20, and cytochrome *c* (CYT *c*) and ETC components ATP5A, UQCRC2, SDHB, and NDUFB8 in sWAT (Figure S3A) without affecting respiration in BAT (Figure 3F). Furthermore, TAN-fed mice at thermoneutrality for the entire study duration showed elevated sWAT OCR (Figure 3G), and these increases in sWAT OCR relied on UCP1, since $Ucp1^{-/+}$ sWAT failed to increase its OCR to the levels in TAN-fed $Ucp1^{+/+}$ mice (Figure 3G). By contrast, TAN feeding did not impact OCR in BAT or eWAT at thermoneutrality (Figure 3G). Interestingly, circadian respirometry revealed sustained increases in sWAT OCR in TAN-fed mice in the dark cycle (Figure 3H), with only minor excursions observed in BAT (Figure S3B). Taken together, TAN feeding leads to marked transcriptional, cellular, and functional remodeling of sWAT.

Innervation-dependent sWAT remodeling, metabolic flexibility, and inflammation resolution in TAN mice

Although we observed a trend toward increased innervation upon TAN feeding, it is well established that sympathetic tone supports adipose browning,[10] and therefore it is possible that TAN feeding increases functional sympathetic tone to support sWAT remodeling. Accordingly, we used depot-specific denervation of sWAT (DNV^{sWAT}) or BAT (DNV^{BAT}) (plans in Figures 4A and 4G) to answer whether TAN-induced sWAT remodeling/energy expenditure requires innervation and whether sWAT remodeling is sufficient to dampen eWAT inflammation. BAT denervation (DNV^{BAT}) was confirmed by reduced expression of *Ucp1* (Figure S4A), adrenergic receptor *Adrb3* (Figure S4B), and BAT marker *Eva1a* (Figure S4C) and the failure to mobilize lipid in response to TAN feeding compared to sham-operated innervated Con (Figure S4D). Bilateral sWAT denervation (DNV^{sWAT}) did not affect *Ucp1*, *Adrb3*, or *Eva1a* expression in BAT (Figures S4A–S4C) but reduced sWAT TH levels (Figure S4E) and blocked adipocyte size reduction in response to TAN feeding (Figure S4F). Consistent with the idea that innervation supports adipocyte metabolic and thermogenic function,[10] TAN-fed DNV^{sWAT} mice failed to lower their sWAT and eWAT weights (Figures 4B and 4C) or induce sWAT OCR compared to TAN-fed Con mice (Figure 4D). Strikingly, BAT OCRs remained unaffected in DNV^{sWAT} mice and their Con group fed ad-lib or TAN (Figure 4E), indicating limited impact of sWAT denervation on BAT energy expenditure. Furthermore, TAN feeding led to innervation-dependent increases in expression of the metabolic elasticity genes *Slc2a5*, *Acly*, *Fasn*, *Elovl6*, and *Ppp1R3b* and decreased expression of *leptin* (Figure 4F)—reflecting improved metabolic flexibility in innervated sWAT of TAN mice.

In contrast, surprisingly, DNV^{BAT} mice and their innervated Cons each displayed equivalent sWAT and eWAT weight loss with similar increases in sWAT OCR in response to TAN feeding (Figures 4H–4J) without notable changes in BAT OCR (Figure 4K). Consistently, sWAT showed equivalent reduction in adipocyte size in TAN-fed Con and DNV^{BAT} mice (Figure S4G), which, in conjunction with similar increases in sWAT OCR in both TAN-fed Con and DNV^{BAT} mice (Figure 4J), indicates maintained higher energy expenditure rates in sWAT of DNV^{BAT} TAN-fed mice. Strikingly, eWAT of Con and DNV^{BAT} mice each showed markedly reduced F4/80 (Figure 4L) and β-galactosidase positivity (Figure S4H) in response to TAN feeding, while eWAT of DNV^{sWAT} mice remained largely inflamed with numerous F4/80-positive crown-like structures despite TAN feeding (Figure 4M). Hence, sWAT remodeling and resolution of eWAT inflammation in TAN-fed mice requires sWAT innervation.

scRNA-seq analyses reveal ILC2 cell recruitment in sWAT of TAN mice

To determine how TAN feeding remodels the sWAT microenvironment, we isolated sWAT stromal vascular fractions (SVF) of 7-month-old male mice fed ad-lib or TAN for 5 months. We performed single-cell RNA sequencing (scRNA-seq) on single-cell suspensions of 5,553 ± 137 cells using the 10× Genomics platform and used Cell Ranger from 10× Genomics for data processing and R package Seurat[11] to generate cell clusters and resolve their identities.[12] Since SVF isolation, scRNA-seq library preparation, and sequencing procedures are each known to introduce variabilities in samples, we performed cluster analysis on integrated and individual libraries from ad-lib and TAN mice and represented these data by t-distributed stochastic neighbor embedding (t-SNE) plots. As seen in Figure S5A, ad-lib and TAN libraries had comparable cell clustering patterns and transcriptomic states. Further subclustering based on known cell-type marker genes identified clusters of group 2 innate lymphoid cells (ILC2), T cells, natural killer T (NKT) cells, B cells, committed preadipocytes (CP) in initial states of differentiation, adipocyte precursor cell (APC) subtypes (APC2, APC1, APC), migratory dendritic cells (migDC), Schwann cells, monocytes/macrophages, endothelial cells, neutrophils, pericytes, and mast cells (Figure 5A). To gain insight into remodeling of stromal cells under ad-lib and TAN conditions, we segregated the cumulative t-SNE-plot into ad-lib and TAN by animal replicate (Figure S5B). The t-SNE plots revealed global changes in relative proportions of SVF clusters between ad-lib and TAN (Figure 5A). Among all clusters, B cells, T cells, and ILC2s appeared to have marked differences in their global transcriptomic profiles in t-SNE 2D projection where cells from ad-lib and TAN were segregated (Figures 5A and S5A), which was

(G) AUC for OCR in sWAT, BAT, and eWAT of Con or $Ucp1^{+/-}$ male mice fed ad-lib or TAN and maintained in thermoneutrality (30°C) for 6 months (n = 5).
(H) AUC for OCR in sWAT collected from ad-lib and TAN-fed RD mice at the six indicated time points (n = 5). Feeding windows are salmon-colored boxes. Values are mean ± SEM. Dot plots show individual values (dots) and mean (line). n.s., non-significant; *$p < 0.05$, **$p < 0.01$, ***$p < 0.001$, ****$p < 0.0001$. Two-way ANOVA and Tukey-corrected (A, F, G) and two-tailed unpaired Student’s t test (B, D, E). Magnifications bars are shown. See also Figure S2.

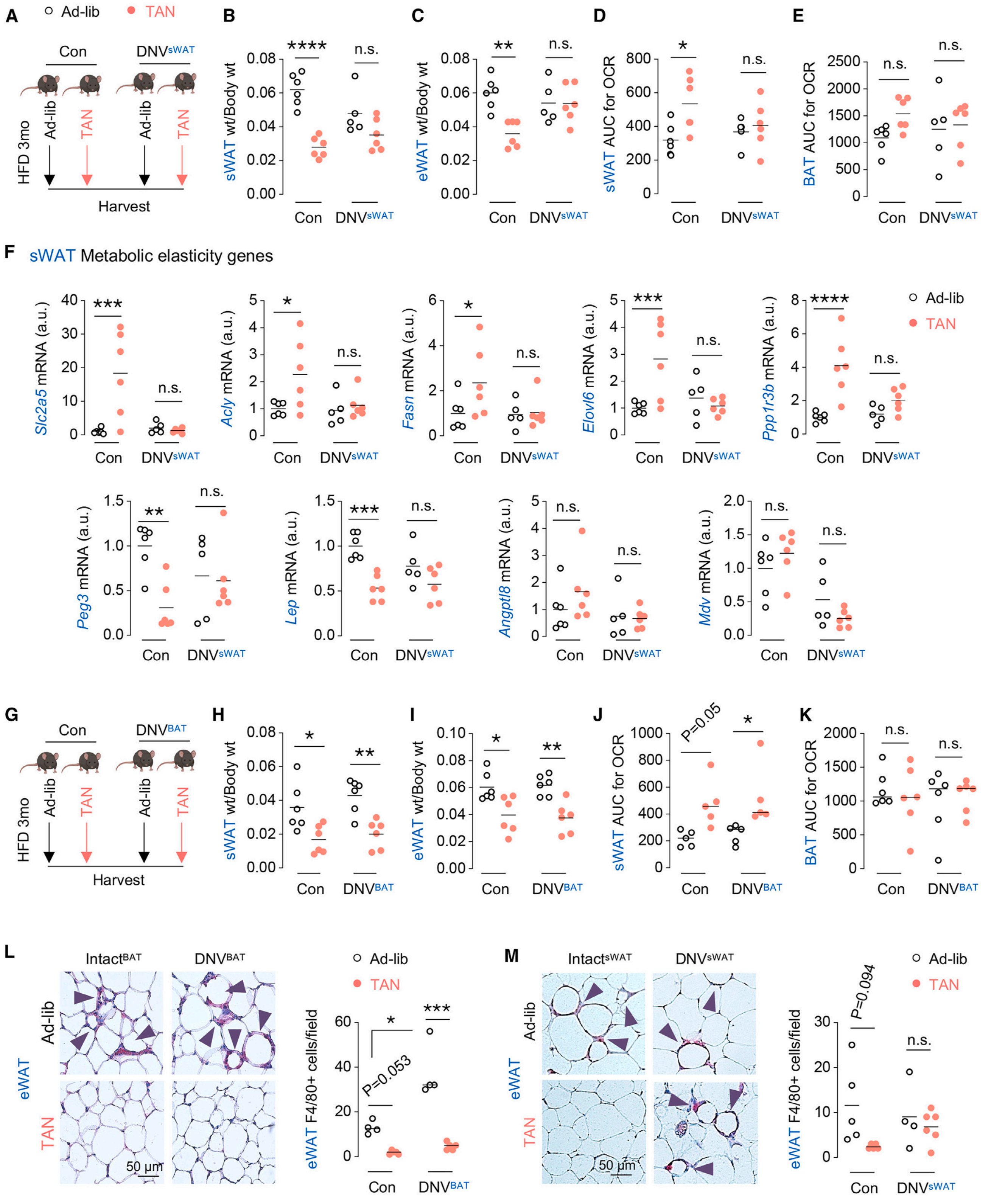

(legend on next page)

validated by further subclustering of B cells, T cells, and ILC2s by percentage (ad-lib, B cells 5.1%, T cells 1.4%, ILC2 2%; TAN, B cells 28.9%, T cells 7.5%, ILC2 3.5%) (Figures 5B and S5C).

Since adipose-resident ILC2s drive white-fat browning,[13,14] we focused on TAN feeding-induced changes in ILC2 clusters enriched with the canonical ILC2 markers *Ilrl1* and *Gata3*[13] (Figures S5D and S5E). To quantitatively determine the transcriptional impact of TAN feeding on ILC2s, we characterized DEGs as a function of cluster type and found a high degree of transcriptional variation in ILC2s in response to TAN feeding (Figure 5C). Interestingly, ILC2 clusters showed remarkable transcriptional differences in cell clusters between ad-lib and TAN, implicating a potential remodeling of ILC2s upon TAN feeding (Figure 5C). Indeed, DEG analysis of ad-lib and TAN ILC2 subclusters showed upregulation of several genes, including *Tnfrs4* and *Ccr7*, which act as licensing signals for ILC2 tissue-specific adaptive immunity and migration[15,16] (Figures 5C and S5F), and gene ontology (GO) analysis showing enrichment of processes related to "cell proliferation," "protein translation," and "transcription" (Figure S5G).

Flow cytometry confirms ILC2 cell recruitment in innervated sWAT of TAN mice

Consistent with scRNA-seq analyses, fluorescence-activated cell sorting (FACS) in sWAT SVFs showed increased ILC2 percentages in TAN-fed mice (Figures 5D and 5E). However, percentages of T and B cells by FACS were comparable between ad-lib and TAN mice (Figures S5H–S5J), indicating potential discrepancies in transcript abundance and protein cell-surface expression. Interestingly, TAN-driven increases in ILC2s were dampened in DNV^{sWAT} (Figure 5E), correlating with reduced OCR and metabolic flexibility gene expression in TAN-fed DNV^{sWAT} mice (Figures 4D and 4F). Since the alarmin cytokine interleukin-33 (IL-33) drives ILC2 proliferation and function,[17] and because sWAT APCs/stromal stem cells are major sources of IL-33,[17,18] we tested if TAN feeding perturbs APC IL-33. Indeed, subclustering APCs (APC, APC1, and APC2) revealed significantly higher percentages of APCs producing IL-33 upon TAN feeding (Figures 5F, 5G, S5K, and S5L), which was confirmed by increased IL-33 protein levels in sWAT of TAN-fed mice (Figure 5H). Interestingly, DEG analysis on APCs showed an increase in the fatty acid metabolic genes *Fabp4* and *Cd36* (Figures 5I–5K), recently shown to be essential for beige APC proliferation and differentiation.[19] Despite these findings, FACS analyses did not show an overall difference in APC population from ad-lib and TAN-fed mice in innervated Con or DNV^{sWAT} mice (Figures 5L and 5M). We also observed a highly CP (Figure 5A) cluster that expressed some markers of adipocyte genes. We base this possibility on the high expression of mitochondrial, lipid metabolism, and adipokine markers (Figure 5A) such as *Adipoq*, *Apoe*, *Fasn*, *Acly*, *Elovol6*, *Agpat2*, *mt-Atp6*, *mt-Cytb*, and *mt-Co2*.[20] Analyses of DEGs in this adipocyte pool revealed an upregulation of metabolic flexibility genes (Figures 2B and S5M), e.g., *Fasn*, *Me1*, and *Elovl6*, as observed in sWAT of TAN-fed mice (Figure 2B), indicating metabolic reprogramming across different adipocyte clusters by TAN feeding.

To investigate whether ILC2 enrichment correlates with sWAT metabolism, we performed bulk RNA-seq on whole sWAT from ad-lib and TAN mice (Figure 5N), which revealed distinct molecular signatures of lipogenesis, glucose metabolism, fatty acid metabolism, mitochondrial function, and thermogenesis in sWAT of TAN mice (Figure 5O). Taken together, expansion and activation of sWAT resident ILC2s support an anti-inflammatory and pro-thermogenic milieu in sWAT in TAN-fed mice, as also reported with CR.[1]

Insulin and leptin oscillations and ILC2 cells are each required for sWAT remodeling

Since leptin-driven pathways drive white-fat browning,[21] we asked if insulin and leptin surges contribute to sWAT remodeling and whether remodeling occurs through an interplay with sWAT-recruited ILC2 cells (Figure 6A). Accordingly, we used four readouts as markers for sWAT remodeling and systemic benefit in TAN mice, i.e., sWAT size, sWAT OCR, sWAT ILC2 cell recruitment, and resolution of eWAT inflammation. Specifically, we tested if dampening leptin or insulin surges or depleting IL-33

Figure 4. Innervation-dependent sWAT remodeling and metabolic flexibility in TAN mice

(A) Scheme showing innervated control (Con) or sWAT denervated (DNV^{sWAT}) C57BL/6J male mice fed ad-lib or TAN on HFD for 3 months.

(B and C) Weights of sWAT (g/g body wt) (B) and eWAT (g/g body wt) (C) from Con or DNV^{sWAT} C57BL/6J male mice fed ad-lib ($n = 6$ Con, $n = 5$ DNV^{sWAT}) or TAN ($n = 6$ Con, $n = 6$ DNV^{sWAT}) on HFD for 3 months.

(D and E) AUC for OCR in sWAT (D) and BAT (E) from Con or DNV^{sWAT} C57BL/6J male mice fed ad-lib ($n = 6$ Con, $n = 4$ DNV^{sWAT}) or TAN ($n = 5$ Con, $n = 6$ DNV^{sWAT}) on HFD for 3 months.

(F) qPCR for metabolic elasticity genes in sWAT from Con or DNV^{sWAT} C57BL/6J male mice fed ad-lib ($n = 6$ Con, $n = 5$ DNV^{sWAT}) or TAN ($n = 6$ Con, $n = 6$ DNV^{sWAT}) on HFD for 3 months.

(G) Scheme showing innervated control (Con) or BAT denervated (DNV^{BAT}) C57BL/6J male mice fed ad-lib ($n = 6$ Con, $n = 7$ DNV^{BAT}) or TAN ($n = 6$ Con, $n = 7$ DNV^{BAT}) on HFD for 3 months.

(H and I) Weights of sWAT (g/g body wt) (H) and eWAT (g/g body wt) (I) from Con or DNV^{BAT} C57BL/6J male mice fed ad-lib or TAN on HFD for 3 months ($n = 6$).

(J and K) AUC for OCR in sWAT (J) and BAT (K) from Con or DNV^{BAT} C57BL/6J male mice fed ad-lib (n = 5–6 Con, n = 5–6 DNV^{BAT}) or TAN (n = 5–6 Con, n = 5–6 DNV^{BAT}) on HFD for 3 months.

(L) Representative F4/80 (red) staining in eWAT from control (Intact^{BAT}) and DNV^{BAT} C57BL/6J male mice fed ad-lib ($n = 9$ Con, $n = 4$ DNV^{BAT}) or TAN ($n = 9$ Con, $n = 6$ DNV^{BAT}) on HFD for 3 months. Quantification for number of F4/80$^+$ cells/field (one section/mouse observed with 5× original magnification) is shown. Arrowheads indicate F4/80$^+$ crown-like structures.

(M) Representative F4/80 (red) staining in eWAT from control (Intact^{eWAT}) and DNV^{sWAT} C57BL/6J male mice fed ad-lib ($n = 5$ Con, $n = 4$ DNV^{sWAT}) or TAN ($n = 6$ Con, $n = 6$ DNV^{sWAT}) on HFD for 3 months (n = 4–9). Quantification for number of F4/80$^+$ cells/field (one section/mouse observed with 5× original magnification) is shown. Dot plots show individual values (dots) and mean (line); n.s., non-significant; *$p < 0.05$, **$p < 0.01$, ***$p < 0.001$, ****$p < 0.0001$. Two-way ANOVA and Tukey-corrected. See also Figure S3.

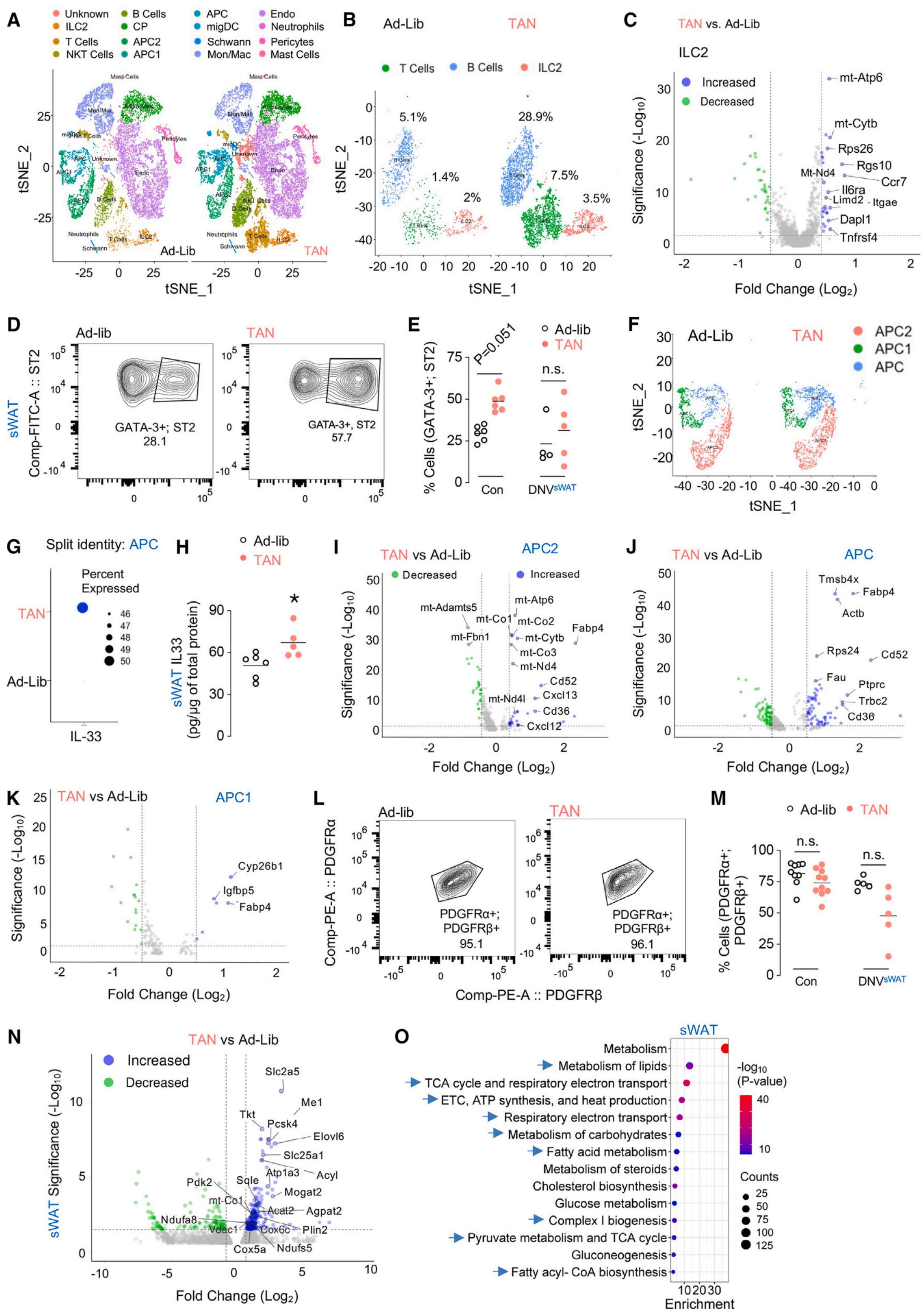

(legend on next page)

to block ILC2 cell recruitment (using *Il33*$^{-/-}$ mice)[13] prevents sWAT remodeling in TAN-fed mice. To test for sufficiency of leptin surges in TAN feeding-induced sWAT remodeling, we used a *leptin*^{KO} (*Ob/Ob*) mouse (Figure S6A). Given the hyperphagia and obesity in *Ob/Ob* mice, we matched body weights of Con and Ob/Ob mice by maintaining *Ob/Ob* mice on RD and the Con mice on HFD (Figure 6B) as conducted previously.[22–24] As noted in Figures 1B and 1C, Con TAN-fed mice (compared to Con ad-lib mice) displayed reduced fat mass (with corresponding increases in fat-free/lean mass) (Figure 6C), decreased sWAT weight (Figure 6D), increased sWAT OCR (Figure 6E), and increased ILC2 cells in sWAT (Figure 6F) without affecting other sWAT immune cells, including FOXP3-positive T regulatory (Treg) cells (Figures S6B–S6H). Consistently, TAN-fed Con mice showed reduced sWAT adipocyte size (Figure 6G) and decreased eWAT inflammation (fewer F4/80-positive crown-like structures) (Figure 6H). By contrast, despite reduced food consumed by TAN-fed *Ob/Ob* compared to TAN-fed Con mice (Figure S6I), TAN-fed *Ob/Ob* mice failed to reduce their fat mass and sWAT weight (Figures 6C and 6D) or stimulate OCR (Figure 6E) or recruit ILC2 cells in sWAT (Figure 6F). Furthermore, *Ob/Ob* sWAT adipocytes remained hypertrophic (Figure 6G) with persistent eWAT inflammation/F4/80 positivity (Figure 6H) despite TAN feeding, suggesting that leptin availability, possibly through its surges, facilitates sWAT remodeling in TAN-fed mice.

Since leptin and insulin oscillate at identical times in response to feeding (Figures 1D and 1E), and encouraged by our findings in *Ob/Ob* mice, we asked if TAN-feeding-induced insulin surges contribute to sWAT remodeling. We employed two distinct models of insulin deficiency (Figure 6I), i.e., conditional whole-body loss of the insulin receptor (*InsR*^{KO}) (by injecting AAV-CMV-Cre into *InsR*$^{Flox/Flox}$ mice) (Figure S6J) and injecting low-dose streptozotocin (STZ) intraperitoneally (i.p.) to dampen insulin signaling (Figure S6K), as we did recently.[25] Since circulating hormonal oscillations likely mediate an effect by acting on multiple cells/tissues, e.g., adipocytes, immune cells, or CNS, we chose to use whole-body gene knockout (KO) models to determine if insulin (or leptin) participates in TAN-feeding-induced sWAT remodeling. As in *Ob/Ob* mice, TAN-feeding-driven increases in sWAT OCR were blunted in both *InsR*^{KO} and low-dose STZ-treated mice (Figures 6J and 6K) without affecting BAT OCR (Figures 6L and 6M). As also observed upon leptin depletion (Figure 6F), ablation of insulin signaling (*InsR*^{KO} mice) blocked the recruitment of ILC2 cells in sWAT (Figure 6N) as did lack of *Il33* (Figure 6N), a factor required for ILC2 recruitment and sWAT beiging.[13] Loss of *Il33* (indicated by loss of GFP) (Figure S6L) did not affect sWAT enrichment of additional cell types, e.g., CD4^{+} T cells or PDGFRα^{+};PDGFRβ^{+} progenitors (Figures S6M and S6N). Supporting the idea that insulin- and leptin-driven, and IL-33-licensed, ILC2 cell recruitment drives sWAT remodeling in TAN-fed mice, loss of leptin or insulin signaling or loss of *Il33* each reduced ILC2 recruitment (Figures 6F and 6N) and blocked TAN-feeding-induced increases in sWAT OCR (Figures 6E, 6J, 6K, and 6O). Interestingly, despite the requirement of IL-33 for recruitment of ILC2 cells in sWAT, increases in IL-33 levels are not regulated by insulin or leptin, since loss of *InsR* (Figure 6P) or leptin (data not shown) did not block TAN-induced increases in sWAT IL-33 levels. Thus, we conclude that leptin, insulin, and ILC33/ILC2 signaling cooperate in the functional remodeling of sWAT in response to TAN feeding (Figure 6A).

Reconstituting insulin and leptin oscillations recapitulate sWAT remodeling

To determine if insulin and leptin oscillations are sufficient to remodel sWAT, we pharmacologically modeled insulin and leptin surges in ad-lib male mice (Figure 7A) in a manner similar to what we observed in TAN-fed mice after their first meal (Figures 1D and 1E). Distinct cohorts of C57BL/6J male mice were injected i.p. daily at 9:30 p.m. for 1.5 months with (1) saline (Con) or (2) insulin (0.16 IU/day) or (3) leptin (5 mg/kg/day) or (4) insulin and leptin both (Co-T$_{x}$ at these doses) (Figure 7A). Because sWAT remodeling and TAN benefits are lost in DNV^{sWAT} mice (Figures 4A–4F), we incorporated an additional group (5) wherein bilateral DNV^{sWAT} mice were subjected to insulin and leptin Co-T$_{x}$ (Figure 7A). To confirm that once-a-day i.p. injections led to a single acute surge in circulating insulin and leptin each day, we measured pre- and postinjected serum levels of insulin or leptin at 1, 2, and 6 h after the injections (Figure 7B). Time-course analyses revealed consistent and sharp increases in circulating insulin and leptin levels 1 h after i.p. administration

Figure 5. scRNA-seq analyses reveal ILC2 cell recruitment in innervated sWAT of TAN mice

(A) t-distributed stochastic neighbor embedding (t-SNE) plots showing cell clustering of sWAT stromal vascular fractions (SVF) from C57BL/6J male mice fed ad-lib or TAN on RD for 5 months (n = 4 each group).

(B) t-SNE subclustering of immune cells from sWAT SVF from C57BL/6J male mice fed ad-lib or TAN on RD for 5 months (n = 4).

(C) Volcano plot showing up- and downregulated genes in sWAT ILC2 cells in TAN-fed mice compared to ad-lib mice (n = 4).

(D and E) Representative contour plots for abundance (D) and quantification for ILC2 cells (E) in sWAT SVF from C57BL/6J male Con or DNV^{sWAT} mice fed ad-lib (n = 6 Con, n = 4 DNV^{sWAT}) or TAN (n = 6 Con, n = 5 DNV^{sWAT}) on HFD for 3 months.

(F) t-SNE subclustering of adipocyte progenitor cells (APCs) from sWAT SVF of C57BL/6J male mice fed ad-lib or TAN on RD for 5 months (n = 4).

(G) Identity analysis for IL-33 levels in APCs in sWAT SVF from C57BL/6J male mice fed ad-lib or TAN on RD for 5 months (n = 4 each group).

(H) IL-33 protein levels in sWAT from C57BL/6J male mice fed ad-lib (n = 6) or TAN (n = 5) on RD for 5 months.

(I–K) Volcano plots of downregulated (green) and upregulated (blue) genes from APC2 (I), APC (J), and APC1 cells (K) in sWAT SVF of TAN-fed mice compared to ad-lib mice (n = 4 each group).

(L and M) Representative contour plots of APCs in sWAT SVF from ad-lib and TAN-fed mice (L) and quantification for percentage APC population in sWAT SVF from ad-lib (n = 9 Con, n = 5 DNV^{sWAT}) and TAN-fed (n = 10 Con, n = 5 DNV^{sWAT}) or DNV^{sWAT} mice (n = 4–6).

(N) Volcano plot of down- (green) and upregulated (blue) genes from bulk RNA-seq of sWAT of TAN-fed mice compared to ad-lib mice (n = 4 each group).

(O) Bubble plot of Reactome showing the top 14 upregulated pathways. Bubble size represents number of genes per pathway, the y axis represents the percentage of enrichment, and bubble color represents -$\log_{10} p$. Arrows highlight metabolism-related pathways (n = 4 each group). Dot plots show individual values (dots) and mean (line); n.s., not significant; *$p < 0.05$. Two-way ANOVA and Tukey-corrected (E and M) and two-tailed unpaired Student's t test (H). See also Figure S4.

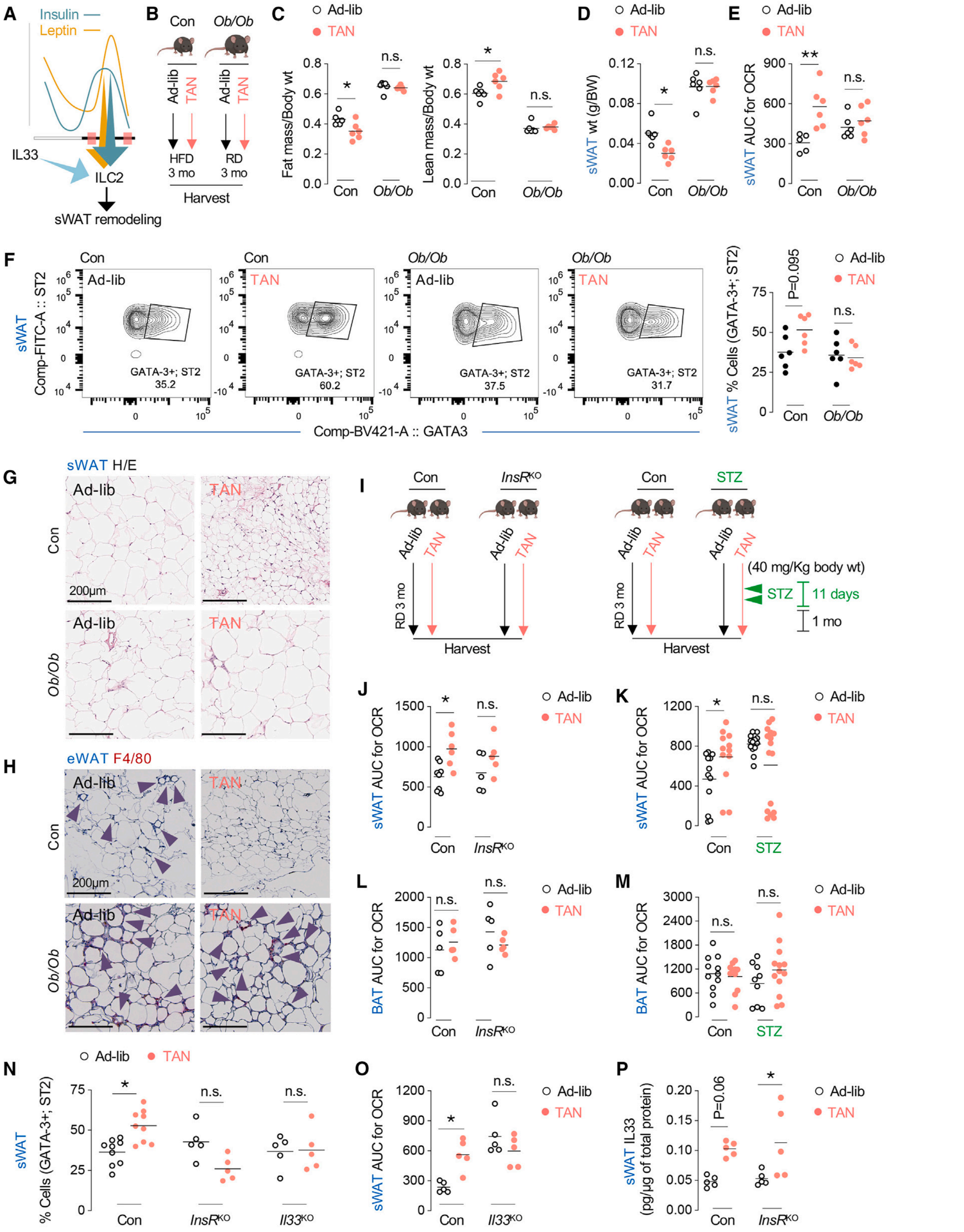

(legend on next page)

CellPress
OPEN ACCESS

(time 0) (Figure 7B), which returned to baseline by 2 h (for insulin) or 6 h (for leptin) (Figure 7B).

Ad-lib injected mice in each group consumed the same amount of food per day when analyzed after 1.5 months (Figure 7C). Individual daily injections of insulin or leptin alone did not affect body composition (Figure 7D), although insulin per se led to expansion of sWAT (trend) and eWAT wt (*$p < 0.05$) (Figures 7E and 7F), likely due to insulin's ability to support adipocyte growth and differentiation.[26] Strikingly, and in clear contrast, Co-T_x of insulin and leptin led to reduced fat mass with corresponding increases in relative lean mass (Figure 7D), reduced sWAT and eWAT weights (Figures 7E and 7F), and increased sWAT OCR without affecting BAT OCR (Figures 7G and 7H). Interestingly, Co-T_x of insulin and leptin led to increased sWAT IL-33 levels without affecting the percentage of ILC2 cells in the sWAT (Figures 7I and 7J), although leptin alone increases the percentage of ILC2 cells in sWAT (Figure 7J) without affecting sWAT IL-33 levels (Figure 7I). These results suggest that, in principle, a single surge of insulin and leptin each day over 1.5 months is sufficient to recapitulate the benefits of TAN feeding.

Consistent with increases in ILC2 recruitment and sWAT OCR, and in accordance with the hypothesis that insulin and leptin oscillations support sWAT remodeling, leptin alone reduced sWAT adipocyte size compared to Con or insulin-injected mice (Figure 7K). However, it is the Co-T_x of insulin and leptin that markedly remodels sWAT as indicated by decreased adipocyte size and recruitment of multiloculated UCP1-positive adipocytes in sWAT (Figure 7K). Furthermore, Co-T_x of insulin and leptin increased the numbers of mitochondrial (CYT *c* and TOMM20) and oxidation/phosphorylation (OXPHOS) proteins (NDUFB8 [complex I], SDHB [II], UQCRC2 [III], MTCO1 [IV]) (Figures 7L and 7M) and increased the expression of recently identified markers[9] for metabolic flexibility, *Slc2a5*, *Fas*, *Elovl6*, *Angptl8*, *Mvd*, and *Ppp1e3b* (Figure S7A), as also noted in TAN-fed mice (Figure 2B). Interestingly, insulin alone contributed to increases in levels of TOMM20 and ATP5A (Figures 7L and 7M), as shown in human skeletal muscle.[27] Consistently, TAN-feeding-induced increases in mitochondrial and OXPHOS markers (Figure S3A) appear to be insulin mediated, since this effect is dampened in TAN-fed *InsR*^{KO} mice compared to TAN-fed Con (Figures S7B–S7D).

Consistent with the observation that benefits of TAN feeding are diminished in DNV^{sWAT} mice (Figures 4A–4F), Co-T_x-induced increases in sWAT OCR, sWAT IL-33 levels, and sWAT remodeling represented by multilocularity/UCP1-positivity and increases in mitochondrial/OXPHOS markers, as well as metabolic flexibility gene expression, were all markedly reduced by sWAT denervation (Figures 7G, 7I, 7K, 7L, and S7A). Taken together, these results show that oscillations of insulin and leptin engage with a CNS-immune axis to cooperatively drive sWAT remodeling.

DISCUSSION

CR[1] and intermittent fasting[3] each leads to sWAT browning, and at least in the case of CR, a role of ILC2 cells is implicated[1]; however, the integrative mechanism for sWAT browning in models of intermittent feeding remains unclear. Here, we show that TAN feeding, separated by periods of fasting, leads to meal-driven surges in circulating insulin and leptin. We suspect that these endocrine surges represent their entrainment by feeding and are unrelated to the central clock given the maintenance of feeding-driven insulin and leptin oscillations in mice housed under a 24 h dark cycle. Hormones that respond to changes in nutrient availability, e.g., ghrelin and glucagon, or FGF21, which oscillates with TAD feeding,[3] may potentially contribute to sWAT remodeling. However, since this study focused on how timed feeding remodels sWAT; we did not examine how fasting-responsive hormones, e.g., ghrelin, glucagon, or FGF21, are altered in TAN-fed mice. Our results present a framework to

Figure 6. Insulin and leptin oscillations and ILC2 cells drive sWAT remodeling in TAN mice
(A) Model for feeding-induced insulin and leptin oscillations driving ILC2 recruitment to stimulate WAT remodeling.
(B and C) Scheme (B) and body composition (C) (fat and lean mass [relative to body wt]) for Con or *leptin*^{KO} (*Ob/Ob*) male mice fed ad-lib (*n* = 6 Con, *n* = 6 *Ob/Ob*) or TAN (*n* = 6 Con, *n* = 6 *Ob/Ob*) on HFD (Con) or RD (*Ob/Ob*) for 3 months.
(D) sWAT weight (g/body wt) from Con or *Ob/Ob* male mice fed ad-lib (*n* = 6 Con, *n* = 6 *Ob/Ob*) or TAN (*n* = 6 Con, *n* = 6 *Ob/Ob*) on HFD (Con) or RD (*Ob/Ob*) for 3 months.
(E) AUC for OCR in sWAT from Con or *Ob/Ob* male mice fed ad-lib (*n* = 5 Con, *n* = 6 *Ob/Ob*) or TAN (*n* = 6 Con, *n* = 6 *Ob/Ob*) on HFD (Con) or RD (*Ob/Ob*) for 3 months (*n* = 5–6).
(F) Representative contour plots/quantification for percentage GATA-3$^+$;ST2$^+$ ILC2 cells in sWAT from Con or *Ob/Ob* male mice fed ad-lib (*n* = 6 Con, *n* = 6 *Ob/Ob*) or TAN (*n* = 6 Con, *n* = 6 *Ob/Ob*) on HFD (Con) or RD (*Ob/Ob*) for 3 months.
(G and H) Representative H&E (G) and F4/80-positive (H) cells in eWAT from Con or *Ob/Ob* male mice fed ad-lib or TAN on HFD (Con) or RD (*Ob/Ob*) for 3 months. Arrows (H) highlight crown-like structures.
(I) Scheme showing *InsR*^{KO} mice and low-dose streptozotocin (STZ)-injected insulin-deficient C57BL/6J male mice (generated as depicted) and their corresponding sex- and age-matched controls.
(J) AUC for OCR in sWAT from mice in (I). Con or *InsR*^{KO} mice were fed ad-lib (*n* = 8 Con, *n* = 5 *InsR*^{KO}) or TAN (*n* = 6 Con, *n* = 5 *InsR*^{KO}) on RD for 5 months.
(K) AUC for OCR in sWAT from mice in (I). Con or STZ-injected mice were fed ad-lib (*n* = 12 Con, *n* = 12 STZ) or TAN (*n* = 12 Con, *n* = 13 STZ) on RD for 5 months.
(L) AUC for OCR in BAT from mice in (I). Con or *InsR*^{KO} mice were fed ad-lib (*n* = 5 Con, *n* = 5 *InsR*^{KO}) or TAN (*n* = 5 Con, *n* = 5 *InsR*^{KO}) on RD for 5 months.
(M) AUC for OCR in BAT from mice described in (I). Con or STZ-injected mice were fed ad-lib (*n* = 11 Con, *n* = 9 STZ) or TAN (*n* = 10 Con, *n* = 13 STZ) on RD for 5 months.
(N) Quantification for percentage GATA-3$^+$;ST2$^+$ ILC2 cells in sWAT SVF from Con, *InsR*^{KO}, and *Il33*^{KO} mice fed ad-lib or TAN for 5 months (*n* = 9 in Con and *n* = 5 each in *InsR*^{KO} and *Il33*^{KO} groups).
(O) AUC for OCR in sWAT from Con and *Il33*^{KO} mice fed ad-lib or TAN on RD for 5 months (*n* = 5 each group).
(P) ELISA for IL-33 protein levels in sWAT (pg/μg of total protein) from Con and *InsR*^{KO} male mice fed ad-lib or TAN for 3 months (*n* = 5 each group). Dot plots show individual values (dots) and mean (line); n.s., not significant; *$p < 0.05$, **$p < 0.01$. Two-way ANOVA and Tukey-corrected. See also Figure S5.

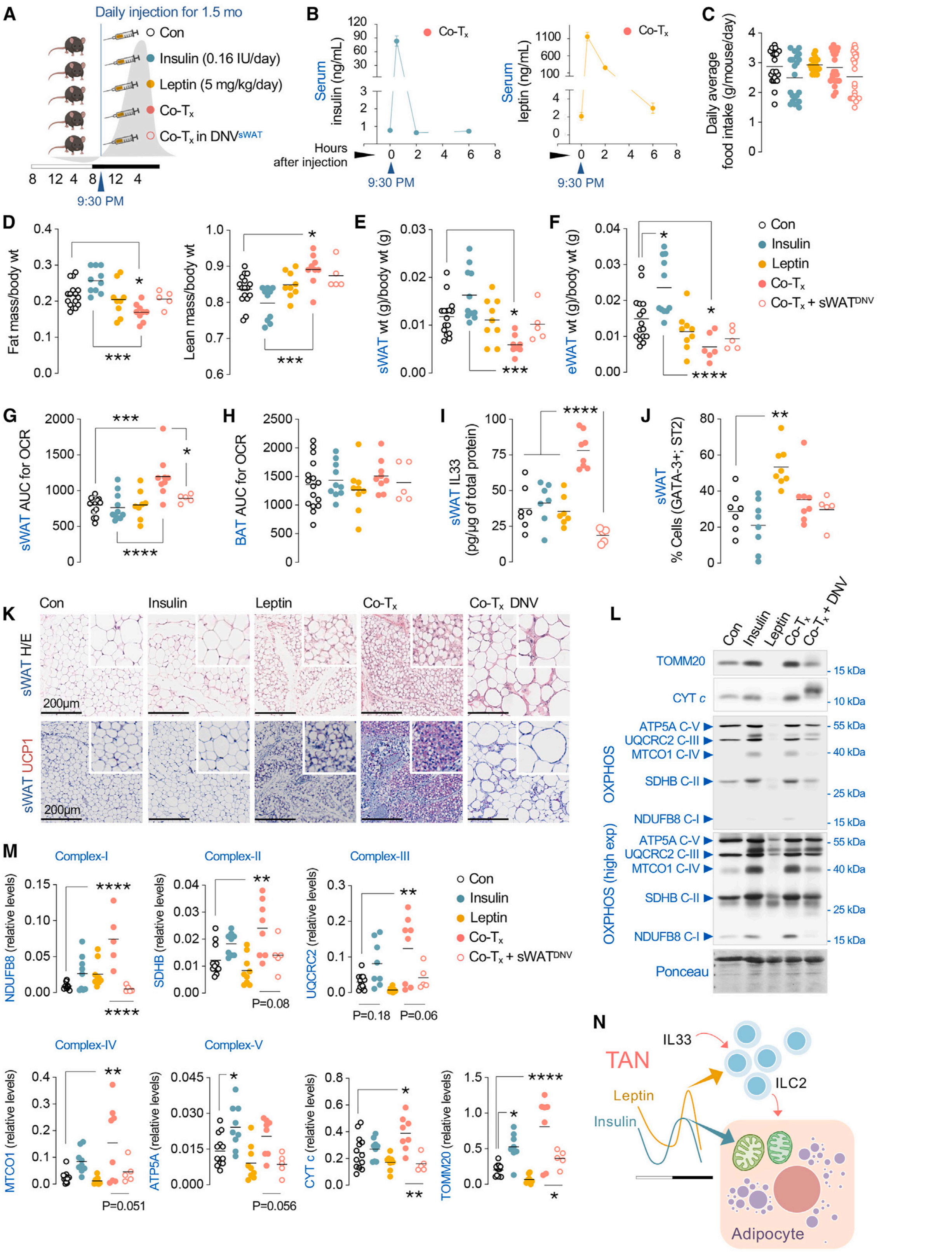

(legend on next page)

consider that feeding-driven surges in insulin and leptin facilitate sWAT remodeling and metabolic improvement and that the observed sWAT plasticity is not solely an effect of fasting. In fact, feeding-driven insulin and leptin oscillations correlate with an active and period-wide rewiring of metabolic programs in sWAT, in that nutrient-driven endocrine surges correlate with enrichment of anabolic pathways that are segregated from catabolic pathways, which reorganize in the fasting windows. Given the increases in oscillations of the recently elucidated metabolic flexibility genes[9] by TAN feeding, we suspect that enrichment of anabolic genes in feeding windows and the synchronization of catabolic pathways with fasting are a representation of metabolic flexibility. Importantly, to our point, TAN-feeding-induced insulin and leptin oscillations are mechanistically linked to sWAT and metabolic remodeling—since increases in sWAT OCR and reduction in sWAT depot/adipocyte size are each blunted when leptin and insulin levels are individually depleted in TAN-fed mice. We argue that insulin and leptin surges per se drive sWAT browning, since pharmacological boluses to recreate hormonal oscillations via once-a-day injections in ad-lib mice are sufficient to remodel sWAT. Intriguingly, significant cellular, functional, and metabolic remodeling of sWAT occurs only in mice co-injected with insulin and leptin, and not in those injected with one or the other, indicating that insulin and leptin cooperatively mediate browning of sWAT. Although continuous i.p. infusions of insulin and leptin have been shown to drive adipose browning on day 6 after the initiation of infusions,[28] it is unclear if sustained infusions will eventually lead to resistance against the action of these hormones, and as a result, the observed browning will likely be lost upon sustained continuous infusions. By contrast, the use of pulsatile once-a-day injections over a prolonged period of time, as conducted in this study, indicated that the timed reconstitution of insulin and leptin oscillations is sufficient per se to sustain adipose browning for prolonged periods of time.

How insulin and leptin surges drive sWAT browning became evident after scRNA-seq analyses of sWAT, which revealed marked increases in the enrichment of ILC2 cells in TAN-fed mice. Although ILC2 cells drive sWAT remodeling/browning,[13] the mechanism of their recruitment to sWAT in models of dietary intervention-induced browning[1] has remained unclear. Our data suggest that intermittent-feeding-induced ILC2 cell recruitment in sWAT is mediated by the surges in levels of insulin and leptin as well as increases in sWAT IL-33 levels. Supporting this idea, depletion of leptin or blocking of insulin receptor signaling each blocked TAN-feeding-induced ILC2 recruitment in sWAT, as did loss of IL-33, a known signal for ILC2 recruitment and sWAT beiging.[13] Although additional immune cells, e.g., eosinophils, have been shown to regulate adipose remodeling, we did not evaluate changes in their levels, and thus, we cannot exclude their potential contribution to TAN-feeding-induced sWAT remodeling.[29] The individual contributions of insulin and leptin toward adipose browning are difficult to parse out given the intricacies and the overlap in their downstream signaling events.[30] However, our data suggest compartmentalization of their roles, in that insulin supports the increases in mitochondrial and OXPHOS proteins, which are important characteristics of adipose browning. Indeed, TAN feeding and insulin injections per se increased mitochondria/OXPHOS proteins, while leptin administration alone led to recruitment of ILC2 cells in sWAT. Thus it would appear that cooperativity between insulin and leptin is required for the cellular, functional, and metabolic remodeling of sWAT in response to TAN feeding.

Supporting the long-held concept that CNS insulin and leptin action[21,28] and depot innervation[10] are required for adipose browning, denervation of sWAT completely blocked each attribute of remodeled sWAT in response to TAN feeding or injections of insulin/leptin. Indeed, DNV^{sWAT} mice failed to deplete their adipose depot weights and drive energy expenditure in response to TAN feeding and injections of insulin/leptin. Tissue innervation also appears to be important for metabolic rewiring and flexibility in response to TAN feeding, since DNV^{sWAT} is unable to drive expression of the metabolic flexibility marker genes *Slc2a5*, *Acly*, *Fasn*, *Elovl6*, and *Ppp1r3b* in TAN-fed and insulin/leptin-co-injected mice. Given these observations, it is tempting to speculate that the insulin/leptin-responsive hypothalamic neurons[31] are likely crucial for mediating the effects of TAN feeding on adipose remodeling. It must also be noted that insulin and leptin impact additional peripheral organs, including liver, and muscle and that the overall systemic benefits of TAN feeding likely involve metabolic reprogramming across multiple tissues.

Our studies show that remodeling and energy expenditure in sWAT mediates the anti-inflammatory effect of TAN feeding on

Figure 7. Reconstituting insulin and leptin oscillations recapitulates sWAT remodeling in mice

(A) Plan for re-creating circulating insulin and leptin surges in C57BL/6J male mice via once-a-day injections of insulin (0.16 IU/day) (n = 10) or leptin (5 mg/kg/day) (n = 9) or both ($Co\text{-}T_x$) (n = 9) at 9:30 p.m. for 1.5 months. Con mice received vehicle (n = 16). $Co\text{-}T_x$ was administered in Con or DNV^{sWAT} (n = 5) mice. All groups were pair-fed to $Co\text{-}T_x$ Con mice.

(B) Serum insulin and leptin levels from mice described in (A) at the indicated time points in response to an acute injection of insulin (0.16 IU/day) or leptin (5 mg/kg/day).

(C) Daily average food intake in mice described in (A) across 47 days of experiment (n = 5–15).

(D) Fat and lean mass (normalized to body weight) for groups defined in (A) (n = 5–16).

(E and F) sWAT and eWAT weight (g/body weight) for groups in (A) (n = 5–16).

(G and H) AUC for OCR in sWAT (G) and BAT (H) for groups in (A) (n = 5–16).

(I) IL-33 protein levels (pg/μg total protein) in sWAT of Con (n = 7), insulin (n = 7), leptin (n = 7), $Co\text{-}T_x$ (n = 8), and DNV^{sWAT} $Co\text{-}T_x$ (n = 5) mice.

(J) Percentage of GATA-3^+;ST2 ILC2 cells in sWAT SVF of Con (n = 7), insulin (n = 8), leptin (n = 8), $Co\text{-}T_x$ (n = 8), and DNV^{sWAT} $Co\text{-}T_x$ (n = 5) mice.

(K) Representative H&E (top) and UCP1 (bottom) staining in sWAT of each group as in (A).

(L and M) Representative immunoblot for indicated proteins in sWAT (L) of each group as in (A) and their corresponding quantifications (M) (n = 5–13). Ponceau is the loading control.

(N) Model for feeding-induced insulin and leptin oscillations, wherein insulin oscillations increase mitochondrial mass and OXPHOS, while leptin oscillations recruit ILC2 cells to cooperatively promote sWAT browning and metabolic flexibility. Dot plots show individual values (dots) and mean (line). *$p < 0.05$, **$p < 0.01$, ***$p < 0.001$, ****$p < 0.0001$. One-way ANOVA and Tukey-corrected (C, D, E, F, G, H, I, J, and M). See also Figure S6.

eWAT. Indeed, denervation of sWAT but not BAT completely blocked the ability of TAN feeding to deplete F4/80$^+$ cells in eWAT (Figure 4L). These data suggest that, perhaps, remodeled sWAT takes center stage when feeding paradigms are perturbed, while the role of BAT is restricted to cold-induced metabolic remodeling and thermogenesis. Taken together, food cues and reorganized endocrine oscillations appear to support the cellular, functional, and metabolic remodeling of sWAT and benefits of timed feeding.

Limitations of the study

One limitation of this study is that it was conducted only in male mice. This is because metabolic disease modeling is best noted in male C57BL/6J mice. However, in appreciation of the importance of understanding sex-specific effects of diets on adipose remodeling, future studies will examine the impact of TAN feeding in female mice. A second weakness is the failure to determine the CNS-specific mechanisms, including the specific cell types in key nutrient-sensitive regions of the brain, that license sWAT browning in response to timed feeding. Finally, this study focused on the role of ILC2s in sWAT browning, and future studies will be required to determine the role of the other sWAT-resident immune cells in sWAT browning in response to TAN feeding.

STAR★METHODS

Detailed methods are provided in the online version of this paper and include the following:

- KEY RESOURCES TABLE
- RESOURCE AVAILABILITY
  - Lead contact
  - Materials availability
  - Data and code availability
- EXPERIMENTAL MODEL AND STUDY PARTICIPANT DETAILS
  - Animals
  - Housing
  - TAN feeding and caloric restriction (CR)
- METHOD DETAILS
  - Tissue collection and injections
  - Creation of knock-out models
  - Subcutaneous-inguinal white and brown adipose tissue denervation
  - Biochemical analyses
  - Western blotting
  - RNA isolation and qPCR analyses
  - RNAseq analysis
  - Tissue respirometry
  - Glucose tolerance test (GTT)
  - Histological analyses and immunohistochemistry
  - Depot-wide imaging and quantifications for adipocyte, vasculature and nerve mass
  - Single cell isolation from sWAT SVF
  - Flow cytometry
  - Gating strategy
  - SVF scRNAseq analysis
- QUANTIFICATION AND STATISTICAL ANALYSIS
- ADDITIONAL RESOURCES

SUPPLEMENTAL INFORMATION

Supplemental information can be found online at https://doi.org/10.1016/j.celrep.2024.114390.

ACKNOWLEDGMENTS

This work was supported by NIH, United States, grants RF1AG043517, R01DK123327, R01AG065985, R01AG082761, and P01AG031782 to R. Singh, and seed support to R. Singh from the Department of Medicine and Vatche and Tamar Manoukian Division of Digestive Diseases at UCLA. The Einstein Flow Cytometry Core is supported by the NIH Shared Instrument Grant (SIG): 1S10OD026833-01.

AUTHOR CONTRIBUTIONS

Conceptualization, R. Singh; methodology, P.M., A.R., G. Schwartz, and R. Singh; feeding, P.M., A.R., C.L., M.K., and R. Singh; investigation, P.M., A.R., N.M.-L., D.D., G. Skovbjerg, J.L.S., U.R., M.K., M.S., R. Subbarayan, and E.P.; writing – original draft, R. Singh; data analysis, P.M., A.R., D.D., G. Skovbjerg, J.L.S., U.R., N.M.-L., M.K., M.S., E.P., J.Z., and P.R.; funding acquisition, R. Singh; resources, J.Z., C.G., X.Z., G. Schwartz, P.R., and R. Singh; supervision, R. Singh.

DECLARATION OF INTERESTS

G. Skovbjerg, J.L.S., and U.R. are employees of Gubra ApS, Hørsholm, Denmark. However, their participation in the study did not impact the outcomes of the study or benefit Gubra in any manner.

Received: December 20, 2023
Revised: April 23, 2024
Accepted: June 6, 2024
Published: June 19, 2024

REFERENCES

1. Fabbiano, S., Suárez-Zamorano, N., Rigo, D., Veyrat-Durebex, C., Stevanovic Dokic, A., Colin, D.J., and Trajkovski, M. (2016). Caloric Restriction Leads to Browning of White Adipose Tissue through Type 2 Immune Signaling. Cell Metabol. *24*, 434–446. https://doi.org/10.1016/j.cmet.2016.07.023.
2. Chaix, A., Lin, T., Le, H.D., Chang, M.W., and Panda, S. (2019). Time-Restricted Feeding Prevents Obesity and Metabolic Syndrome in Mice Lacking a Circadian Clock. Cell Metabol. *29*, 303–319.e4. https://doi.org/10.1016/j.cmet.2018.08.004.
3. Martinez-Lopez, N., Tarabra, E., Toledo, M., Garcia-Macia, M., Sahu, S., Coletto, L., Batista-Gonzalez, A., Barzilai, N., Pessin, J.E., Schwartz, G.J., et al. (2017). System-wide Benefits of Intermeal Fasting by Autophagy. Cell Metabol. *26*, 856–871.e5. https://doi.org/10.1016/j.cmet.2017.09.020.
4. Wu, J., Cohen, P., and Spiegelman, B.M. (2013). Adaptive thermogenesis in adipocytes: is beige the new brown? Genes Dev. *27*, 234–250. https://doi.org/10.1101/gad.211649.112.
5. Becher, T., Palanisamy, S., Kramer, D.J., Eljalby, M., Marx, S.J., Wibmer, A.G., Butler, S.D., Jiang, C.S., Vaughan, R., Schöder, H., et al. (2021). Brown adipose tissue is associated with cardiometabolic health. Nat. Med. *27*, 58–65. https://doi.org/10.1038/s41591-020-1126-7.
6. Cypess, A.M., Lehman, S., Williams, G., Tal, I., Rodman, D., Goldfine, A.B., Kuo, F.C., Palmer, E.L., Tseng, Y.H., Doria, A., et al. (2009). Identification and importance of brown adipose tissue in adult humans. N. Engl. J. Med. *360*, 1509–1517. https://doi.org/10.1056/NEJMoa0810780.
7. Bridges, D., and Saltiel, A.R. (2015). Phosphoinositides: Key modulators of energy metabolism. Biochim. Biophys. Acta *1851*, 857–866. https://doi.org/10.1016/j.bbalip.2014.11.008.
8. Sakers, A., De Siqueira, M.K., Seale, P., and Villanueva, C.J. (2022). Adipose-tissue plasticity in health and disease. Cell *185*, 419–446. https://doi.org/10.1016/j.cell.2021.12.016.

9. Zhou, Q., Yu, L., Cook, J.R., Qiang, L., and Sun, L. (2023). Deciphering the decline of metabolic elasticity in aging and obesity. Cell Metabol. *35*, 1661–1671.e6. https://doi.org/10.1016/j.cmet.2023.08.001.

10. Nguyen, N.L.T., Barr, C.L., Ryu, V., Cao, Q., Xue, B., and Bartness, T.J. (2017). Separate and shared sympathetic outflow to white and brown fat coordinately regulates thermoregulation and beige adipocyte recruitment. Am. J. Physiol. Regul. Integr. Comp. Physiol. *312*, R132–R145. https://doi.org/10.1152/ajpregu.00344.2016.

11. Butler, A., Hoffman, P., Smibert, P., Papalexi, E., and Satija, R. (2018). Integrating single-cell transcriptomic data across different conditions, technologies, and species. Nat. Biotechnol. *36*, 411–420. https://doi.org/10.1038/nbt.4096.

12. Arneson, D., Zhang, G., Ying, Z., Zhuang, Y., Byun, H.R., Ahn, I.S., Gomez-Pinilla, F., and Yang, X. (2018). Single cell molecular alterations reveal target cells and pathways of concussive brain injury. Nat. Commun. *9*, 3894. https://doi.org/10.1038/s41467-018-06222-0.

13. Brestoff, J.R., Kim, B.S., Saenz, S.A., Stine, R.R., Monticelli, L.A., Sonnenberg, G.F., Thome, J.J., Farber, D.L., Lutfy, K., Seale, P., and Artis, D. (2015). Group 2 innate lymphoid cells promote beiging of white adipose tissue and limit obesity. Nature *519*, 242–246. https://doi.org/10.1038/nature14115.

14. Lee, M.W., Odegaard, J.I., Mukundan, L., Qiu, Y., Molofsky, A.B., Nussbaum, J.C., Yun, K., Locksley, R.M., and Chawla, A. (2015). Activated type 2 innate lymphoid cells regulate beige fat biogenesis. Cell *160*, 74–87. https://doi.org/10.1016/j.cell.2014.12.011.

15. Halim, T.Y.F., Rana, B.M.J., Walker, J.A., Kerscher, B., Knolle, M.D., Jolin, H.E., Serrao, E.M., Haim-Vilmovsky, L., Teichmann, S.A., Rodewald, H.R., et al. (2018). Tissue-Restricted Adaptive Type 2 Immunity Is Orchestrated by Expression of the Costimulatory Molecule OX40L on Group 2 Innate Lymphoid Cells. Immunity *48*, 1195–1207.e6. https://doi.org/10.1016/j.immuni.2018.05.003.

16. Mackley, E.C., Houston, S., Marriott, C.L., Halford, E.E., Lucas, B., Cerovic, V., Filbey, K.J., Maizels, R.M., Hepworth, M.R., Sonnenberg, G.F., et al. (2015). CCR7-dependent trafficking of RORγ^{+} ILCs creates a unique microenvironment within mucosal draining lymph nodes. Nat. Commun. *6*, 5862. https://doi.org/10.1038/ncomms6862.

17. Spallanzani, R.G., Zemmour, D., Xiao, T., Jayewickreme, T., Li, C., Bryce, P.J., Benoist, C., and Mathis, D. (2019). Distinct immunocyte-promoting and adipocyte-generating stromal components coordinate adipose tissue immune and metabolic tenors. Sci. Immunol. *4*, eaaw3658. https://doi.org/10.1126/sciimmunol.aaw3658.

18. Mahlakoiv, T., Flamar, A.L., Johnston, L.K., Moriyama, S., Putzel, G.G., Bryce, P.J., and Artis, D. (2019). Stromal cells maintain immune cell homeostasis in adipose tissue via production of interleukin-33. Sci Immunol *4*, eaax0416. https://doi.org/10.1126/sciimmunol.aax0416.

19. Abe, I., Oguri, Y., Verkerke, A.R.P., Monteiro, L.B., Knuth, C.M., Auger, C., Qiu, Y., Westcott, G.P., Cinti, S., Shinoda, K., et al. (2022). Lipolysis-derived linoleic acid drives beige fat progenitor cell proliferation. Dev. Cell *57*, 2623–2637.e8. https://doi.org/10.1016/j.devcel.2022.11.007.

20. Emont, M.P., Jacobs, C., Essene, A.L., Pant, D., Tenen, D., Colleluori, G., Di Vincenzo, A., Jørgensen, A.M., Dashti, H., Stefek, A., et al. (2022). A single-cell atlas of human and mouse white adipose tissue. Nature *603*, 926–933. https://doi.org/10.1038/s41586-022-04518-2.

21. Plum, L., Rother, E., Münzberg, H., Wunderlich, F.T., Morgan, D.A., Hampel, B., Shanabrough, M., Janoschek, R., Könner, A.C., Alber, J., et al. (2007). Enhanced leptin-stimulated Pi3k activation in the CNS promotes white adipose tissue transdifferentiation. Cell Metabol. *6*, 431–445. https://doi.org/10.1016/j.cmet.2007.10.012.

22. Yang, L., Calay, E.S., Fan, J., Arduini, A., Kunz, R.C., Gygi, S.P., Yalcin, A., Fu, S., and Hotamisligil, G.S. (2015). METABOLISM. S-Nitrosylation links obesity-associated inflammation to endoplasmic reticulum dysfunction. Science *349*, 500–506. https://doi.org/10.1126/science.aaa0079.

23. Jimenez, V., Jambrina, C., Casana, E., Sacristan, V., Muñoz, S., Darriba, S., Rodó, J., Mallol, C., Garcia, M., León, X., et al. (2018). FGF21 gene therapy as treatment for obesity and insulin resistance. EMBO Mol. Med. *10*, e8791. https://doi.org/10.15252/emmm.201708791.

24. Skop, V., Guo, J., Liu, N., Xiao, C., Hall, K.D., Gavrilova, O., and Reitman, M.L. (2020). Mouse Thermoregulation: Introducing the Concept of the Thermoneutral Point. Cell Rep. *31*, 107501. https://doi.org/10.1016/j.celrep.2020.03.065.

25. Martinez-Lopez, N., Mattar, P., Toledo, M., Bains, H., Kalyani, M., Aoun, M.L., Sharma, M., McIntire, L.B.J., Gunther-Cummins, L., Macaluso, F.P., et al. (2023). mTORC2-NDRG1-CDC42 axis couples fasting to mitochondrial fission. Nat. Cell Biol. *25*, 989–1003. https://doi.org/10.1038/s41556-023-01163-3.

26. Klemm, D.J., Leitner, J.W., Watson, P., Nesterova, A., Reusch, J.E., Goalstone, M.L., and Draznin, B. (2001). Insulin-induced adipocyte differentiation. Activation of CREB rescues adipogenesis from the arrest caused by inhibition of prenylation. J. Biol. Chem. *276*, 28430–28435. https://doi.org/10.1074/jbc.M103382200.

27. Stump, C.S., Short, K.R., Bigelow, M.L., Schimke, J.M., and Nair, K.S. (2003). Effect of insulin on human skeletal muscle mitochondrial ATP production, protein synthesis, and mRNA transcripts. Proc. Natl. Acad. Sci. USA *100*, 7996–8001. https://doi.org/10.1073/pnas.1332551100.

28. Dodd, G.T., Decherf, S., Loh, K., Simonds, S.E., Wiede, F., Balland, E., Merry, T.L., Münzberg, H., Zhang, Z.Y., Kahn, B.B., et al. (2015). Leptin and insulin act on POMC neurons to promote the browning of white fat. Cell *160*, 88–104. https://doi.org/10.1016/j.cell.2014.12.022.

29. Huang, Z., Zhong, L., Lee, J.T.H., Zhang, J., Wu, D., Geng, L., Wang, Y., Wong, C.M., and Xu, A. (2017). The FGF21-CCL11 Axis Mediates Beiging of White Adipose Tissues by Coupling Sympathetic Nervous System to Type 2 Immunity. Cell Metabol. *26*, 493–508.e4. https://doi.org/10.1016/j.cmet.2017.08.003.

30. Saladin, R., De Vos, P., Guerre-Millo, M., Leturque, A., Girard, J., Staels, B., and Auwerx, J. (1995). Transient increase in obese gene expression after food intake or insulin administration. Nature *377*, 527–529. https://doi.org/10.1038/377527a0.

31. Porniece Kumar, M., Cremer, A.L., Klemm, P., Steuernagel, L., Sundaram, S., Jais, A., Hausen, A.C., Tao, J., Secher, A., Pedersen, T.Å., et al. (2021). Insulin signalling in tanycytes gates hypothalamic insulin uptake and regulation of AgRP neuron activity. Nat. Metab. *3*, 1662–1679. https://doi.org/10.1038/s42255-021-00499-0.

32. Furman, B.L. (2015). Streptozotocin-Induced Diabetic Models in Mice and Rats. Curr. Protoc. Pharmacol. *70*, 5.47.1–5.47.20. https://doi.org/10.1002/0471141755.ph0547s70.

33. Pei, H., Qu, Y., Lu, X., Yu, Q., Lian, K., Liu, P., Yan, W., Liu, J., Ma, Y., Liu, Y., et al. (2013). Cardiac-derived adiponectin induced by long-term insulin treatment ameliorates myocardial ischemia/reperfusion injury in type 1 diabetic mice via AMPK signaling. Basic Res. Cardiol. *108*, 322. https://doi.org/10.1007/s00395-012-0322-0.

34. Billes, S.K., Simonds, S.E., and Cowley, M.A. (2012). Leptin reduces food intake via a dopamine D2 receptor-dependent mechanism. Mol. Metabol. *1*, 86–93. https://doi.org/10.1016/j.molmet.2012.07.003.

35. Martinez-Lopez, N., Garcia-Macia, M., Sahu, S., Athonvarangkul, D., Liebling, E., Merlo, P., Cecconi, F., Schwartz, G.J., and Singh, R. (2016). Autophagy in the CNS and Periphery Coordinate Lipophagy and Lipolysis in the Brown Adipose Tissue and Liver. Cell Metabol. *23*, 113–127. https://doi.org/10.1016/j.cmet.2015.10.008.

36. Kirst, C., Skriabine, S., Vieites-Prado, A., Topilko, T., Bertin, P., Gerschenfeld, G., Verny, F., Topilko, P., Michalski, N., Tessier-Lavigne, M., and Renier, N. (2020). Mapping the Fine-Scale Organization and Plasticity of the Brain Vasculature. Cell *180*, 780–795.e25. https://doi.org/10.1016/j.cell.2020.01.028.

37. Hafemeister, C., and Satija, R. (2019). Normalization and variance stabilization of single-cell RNA-seq data using regularized negative binomial regression. Genome Biol. *20*, 296. https://doi.org/10.1186/s13059-019-1874-1.

38. Stuart, T., Butler, A., Hoffman, P., Hafemeister, C., Papalexi, E., Mauck, W.M., 3rd, Hao, Y., Stoeckius, M., Smibert, P., and Satija, R. (2019). Comprehensive Integration of Single-Cell Data. Cell *177*, 1888–1902.e21. https://doi.org/10.1016/j.cell.2019.05.031.

39. Burl, R.B., Ramseyer, V.D., Rondini, E.A., Pique-Regi, R., Lee, Y.H., and Granneman, J.G. (2018). Deconstructing Adipogenesis Induced by beta3-Adrenergic Receptor Activation with Single-Cell Expression Profiling. Cell Metabol. *28*, 300–309.e304. https://doi.org/10.1016/j.cmet.2018.05.025.

40. Chen, J., Cheung, F., Shi, R., Zhou, H., Lu, W., CHI Consortium; Kotliarov, Y., Stagliano, K.R., Tsang, J.S., and Consortium, C.H.I. (2018). PBMC fixation and processing for Chromium single-cell RNA sequencing. J. Transl. Med. *16*, 198. https://doi.org/10.1186/s12967-018-1578-4.

41. Han, X., Wang, R., Zhou, Y., Fei, L., Sun, H., Lai, S., Saadatpour, A., Zhou, Z., Chen, H., Ye, F., et al. (2018). Mapping the Mouse Cell Atlas by Microwell-Seq. Cell *172*, 1091–1107.e17. https://doi.org/10.1016/j.cell.2018.02.001.

42. Hepler, C., Shan, B., Zhang, Q., Henry, G.H., Shao, M., Vishvanath, L., Ghaben, A.L., Mobley, A.B., Strand, D., Hon, G.C., and Gupta, R.K. (2018). Identification of functionally distinct fibro-inflammatory and adipogenic stromal subpopulations in visceral adipose tissue of adult mice. Elife *7*, e39636. https://doi.org/10.7554/eLife.39636.

43. Rajbhandari, P., Arneson, D., Hart, S.K., Ahn, I.S., Diamante, G., Santos, L.C., Zaghari, N., Feng, A.C., Thomas, B.J., Vergnes, L., et al. (2019). Single cell analysis reveals immune cell-adipocyte crosstalk regulating the transcription of thermogenic adipocytes. Elife *8*, e49501. https://doi.org/10.7554/eLife.49501.

44. Stoeckius, M., Hafemeister, C., Stephenson, W., Houck-Loomis, B., Chattopadhyay, P.K., Swerdlow, H., Satija, R., and Smibert, P. (2017). Simultaneous epitope and transcriptome measurement in single cells. Nat. Methods *14*, 865–868. https://doi.org/10.1038/nmeth.4380.

45. Zhang, X., Lan, Y., Xu, J., Quan, F., Zhao, E., Deng, C., Luo, T., Xu, L., Liao, G., Yan, M., et al. (2019). CellMarker: a manually curated resource of cell markers in human and mouse. Nucleic Acids Res. *47*, D721–D728. https://doi.org/10.1093/nar/gky900.

46. Tabula Muris Consortium; Overall coordination; Logistical coordination; Organ collection and processing; Library preparation and sequencing; Computational data analysis; Cell type annotation; Writing group; Supplemental text writing group; Principal investigators (2018). Single-cell transcriptomics of 20 mouse organs creates a Tabula Muris. Nature *562*, 367–372. https://doi.org/10.1038/s41586-018-0590-4.

47. Li, C.M.C., Shapiro, H., Tsiobikas, C., Selfors, L.M., Chen, H., Rosenbluth, J., Moore, K., Gupta, K.P., Gray, G.K., Oren, Y., et al. (2020). Aging-Associated Alterations in Mammary Epithelia and Stroma Revealed by Single-Cell RNA Sequencing. Cell Rep. *33*, 108566. https://doi.org/10.1016/j.celrep.2020.108566.

48. Jassal, B., Matthews, L., Viteri, G., Gong, C., Lorente, P., Fabregat, A., Sidiropoulos, K., Cook, J., Gillespie, M., Haw, R., et al. (2020). The reactome pathway knowledgebase. Nucleic Acids Res. *48*, D498–D503. https://doi.org/10.1093/nar/gkz1031.

STAR★METHODS

KEY RESOURCES TABLE

REAGENT or RESOURCE	SOURCE	IDENTIFIER
Antibodies		
Alexa Fluor 700 anti-mouse Ly-6A/E (Sca-1)	Biolegend	Cat: #108142; RRID: AB_2565958
Alexa Fluor 488, CD34 Rat anti-Mouse	Thermo Fisher Scientific	Cat: #53034180; RRID: AB_2866439
APC anti-mouse CD140a (PDGFR-α)	Biolegend	Cat: #135907; RRID: AB_2043970
APC anti-mouse Ki-67	Biolegend	Cat: #652406; AB_2561930
eFluor^{TM} 660FOXP3 Monoclonal Antibody (FJK-16s)	Thermo Fisher Scientific	Cat: #50577382; RRID: AB_11218868
Beta Galactosidase Polyclonal	Thermo Fisher	Cat: #PA5-102503; RRID: PA5-102503
BV421 Mouse Anti-Ki-67	BD Biosciences	Cat: #562899; RRID: AB_2686897
BV421 Mouse Anti-GATA3	BD Biosciences	Cat: #563349; RRID: AB_2738152
Brilliant Violet 510^{TM} anti-mouse/human CD11b	Biolegend	Cat: #101245; RRID: AB_2561390
Brilliant Violet 605^{TM} anti-mouse CD127 (IL-7Rα)	Biolegend	Cat: #135041; RRID: AB_2572047
Brilliant Violet 650^{TM} anti-mouse CD206 (MMR)	Biolegend	Cat: #141723; RRID: AB_2562445
Brilliant Violet 711^{TM} anti-mouse CD11c	Biolegend	Cat: #117349; RRID:AB_2563905
Brilliant Violet 785^{TM} anti-mouse CD31	Biolegend	Cat: #102435; RRID: AB_2810334
BUV395 Rat Anti-Mouse CD4	BD Biosciences	Cat: #563790; RRID: AB_2738426
BUV496 Rat Anti-Mouse CD45R/B220	BD Biosciences	Cat: #612950; RRID: AB_2870227
BUV737 Rat Anti-Mouse CD24	BD Biosciences	Cat: #612832; RRID: AB_2870154
BUV737 Rat Anti-Mouse CD8a	BD Biosciences	Cat: #612759; RRID: AB_2870090
BUV805 Rat Anti-Mouse CD3 molecular complex	BD Biosciences	Cat: #741982; RRID: AB_2871285
T1/ST2 (IL33 R) Mouse monoclonal	MD Biosciences	Cat: #101001F; RRID: AB_947549
PE anti-mouse CD140b (PDGFR-β)	Biolegend	Cat: #136005; RRID: AB_1953270
PE/Dazzle^{TM} 594 anti-mouse NK-1.1	Biolegend	Cat: #108748; RRID: AB_2564218
PE/Cyanine5 anti-mouse F4/80	Biolegend	Cat: #123112; RRID: AB_893494
PE/Cyanine7 anti-mouse/rat CD29	Biolegend	Cat: #102221; RRID: AB_528789
PE/Cyanine7 anti-mouse CD25	Biolegend	Cat: #102016; RRID: AB_312865
PerCP anti-mouse CD19	Biolegend	Cat: #115532; RRID: AB_2072926
PerCP/Cyanine5.5 anti-mouse CD45	Biolegend	Cat: #103131; RRID: AB_893344
F4/80 antibody [SP115]	Abcam	Cat# ab111101; RRID:AB_10859466
Recombinant Anti-UCP1	Abcam	Cat# ab234430; RRID:AB_2905638
Total OXPHOS Rodent WB Antibody Cocktail	Abcam	Cat# ab110413; RRID:AB_2629281
Voltage-dependent anion channel (VDAC)	Abcam	Cat# ab15895; RRID:AB_2214787
Cytochrome C (CYT c)	Cell Signaling Technology	Cat# 11940; RRID:AB_2637071
TOMM20	Cell Signaling Technology	Cat# 42406; RRID:AB_2687663
GFP	Novus	Cat# NB100-1770; RRID:AB_10128178
Insulin Receptor β	Cell Signaling Technology	Cat# 3025; RRID:AB_2280448
ImmPACT Vector Red Substrate Kit	Vector Laboratories	Cat# SK-5105; RRID:AB_2336524
ImmPRESS-AP Anti-Rabbit Ig Reagent antibody	Vector Laboratories	Cat# MP-5401; RRID:AB_2336536
Secondary HRP Antibody Rabbit anti-Mouse IgG	Thermo Fisher Scientific	Cat# 61-6520; RRID:AB_2533933
KPL Peroxidase-Labeled Antibody To Rabbit IgG (H+L)	KPL	Cat# 074-1506; RRID:AB_2721169
Bacterial and virus strains		
AAV9-CMV-Null	Vector Biolabs	#7030 (Lot: 220530#57)
AAV9-CMV-iCre	Vector Biolabs	#7098 (Lot: 220530#58)

(*Continued on next page*)

Continued		
REAGENT or RESOURCE	SOURCE	IDENTIFIER
Chemicals, peptides, and recombinant proteins		
ATP	MilliporeSigma	A2383
GHOST DYE RED 780	Tonbo Biosciences	130865-T1CC
Brilliant Buffer	BD Biosciences	563794
Carnitine	MilliporeSigma	C0283
Collagenase Type II	Gibco	17101015
Co-enzyme A	MilliporeSigma	C3144
Digitonin	MilliporeSigma	D5628
D-Glucose	Fisher Scientific	D16-500
Eosin	StatLab	SL98-1
Ethylenediaminetetraacetic acid (EDTA)	American Bioanalytical	AB00500
FcR Blocking Reagent	Miltenyi Biotec	130-092-575
Fixation/Permeabilization Concentrate	Invitrogen	00-5123-43
Fixation/Permeabilization Diluent	Invitrogen	00-5223-56
HBSS 1x	Gibco	14175095
Hematoxylin	Poly-scientific	S212
High Fat Diet (HFD- 60% of calories in fat)	Research Diet	D12492
Recombinant Insulin	MilliporeSigma	11882
Recombinant Leptin	MilliporeSigma	L3772
NAD	MilliporeSigma	N0632
PicoLab Rodent Diet (Regular diet)	Lab Diet	5058
Power SYBR Green PCR Master Mix	Invitrogen	4368708
RBC Lysis Buffer	Biolegend	420301
Rneasy Plus Mini kit	Qiagen	74136
Sodium Chloride	American Bioanalytical	AB01915
Sodium Pyruvate	MilliporeSigma	P2256
Sodium Phosphate, dibasic, anhydrous	American Bioanalytical	AB02050
Super Signal West Femto Maximum Sensitivity Substrate, ECL	Pierce	34096
Western Lightning Plus, Chemiluminescent Substrate	Perkin Elmer	NEL104001EA
Superscript II Reverse Transcriptase	Invitrogen	18064014
Trizol Reagent	Invitrogen	15596018
Triton X-100	Sigma-Aldrich	X100-500ml
30% Acrylamide/ Bis Solution 37-5-1	BioRad	161-0158
SDS	Fisher Scientific	BP8200100
Sodium Deoxycholate	Fisher Scientific	AAJ6228830
Tris HCL	Fisher Scientific	BP152-5
Sigma water	MilliporeSigma	W4502
Complete, EDTA-free	MilliporeSigma	11873580001
Phosphatase Inhibitor Cocktail 3	MilliporeSigma	P0044
Phosphatase Inhibitor Cocktail 2	MilliporeSigma	P5726
Streptozotocin	MilliporeSigma	S0130
Critical commercial assays		
ELISA Corticosterone	Immuno-Biological Laboratories Inc	IB79175
ELISA Insulin Kit	ALPCO	80INSMRCH01

(*Continued on next page*)

Continued

REAGENT or RESOURCE	SOURCE	IDENTIFIER
ELISA IL33 Kit	Bio-Techne Corporation	#DY362605
ELISA Leptin Kit	SPI Bio	A05176
Ascensia Contour Glucometer	Bayer	7151H
Ascensia Contour strips	Bayer	7080G
Deposited data		
Bulk RNA-seq	This Study	GSE264173
scRNA-seq	This Study	GSE263899
Raw Western blotting data	This Study	Mendeley Data: https://doi.org/10.17632/8cftjnncn3.1
Experimental models: Organisms/strains		
Mouse: C57BL6/J	The Jackson Laboratory	Strain #:000664 RRID:IMSR_JAX:000664
Mouse: *Il33*$^{flox/flox}$-eGFP	The Jackson Laboratory	Strain #:030619 RRID:IMSR_JAX:030619
Mouse: *InsR*$^{flox/flox}$	The Jackson Laboratory	Strain #:006955 RRID:IMSR_JAX:006955
Mouse: *Lep*$^{ob/ob}$	The Jackson Laboratory	Strain #:000632 RRID:IMSR_JAX:000632
Mouse: *Ucp1*^{KO}	The Jackson Laboratory	Strain #:003124 RRID:IMSR_JAX:003124
Oligonucleotides		
RT-PCR primers. Please see Table S5		
Software and algorithms		
FlowJo v10.8 software	BD Bioscience	https://www.flowjo.com/; RRID:SCR_008520
ImageJ	NIH	https://ImageJ.nih.gov/ij/index.html; RRID: SCR_003070
Prism	Graph Pad	https://www.graphpad.com/scientificsoftware/prism/; RRID: SCR_002798
R 4.0.4	See quantification and statistical analysis section for R packages used	http://www.r-project.org/R Project for Statistical Computing RRID:SCR_001905
Other		
Zeiss Axiolab 5 microscope/Axiocam 305 color camera with ×10, ×20 and ×40 objectives.	Carl Zeiss Microscopy	N/A
Seahorse Bioscience XF24-3 Extracellular	Seahorse Bioscience (Agilent technologies)	N/A
StepOne Plus Real-Time PCR System	Thermo Fisher Scientific	4376600
Aurora Flow cytometer	Cytek	NA
KwikQuant Digital Western Blot Detection System	Kindle Biosciences, LLC	D0001
ECHO magnetic resonance spectroscopy	Echo Medical Systems	N/A

RESOURCE AVAILABILITY

Lead contact

Further information and requests for resources and reagents should be directed to and will be fulfilled by the lead contact, Rajat Singh (RajatSingh@mednet.ucla.edu).

Materials availability

This study did not generate new reagents.

Data and code availability

- Bulk and Single-cell RNA-seq data have been deposited at GEO and are publicly available on the date of publication. Accession numbers are listed in the key resources table. Original western blot images will be deposited at Mendeley and will be publicly available on the date of publication.
- This paper does not report original code
- Any additional information required to reanalyze the data reported in this paper is available from the lead contact upon request.

EXPERIMENTAL MODEL AND STUDY PARTICIPANT DETAILS

Animals

All procedures were performed under a protocol approved by the Institutional Animal Care and Use Committee (IACUC) of the Albert Einstein College of Medicine. All experiments were performed in 3-4-mo-old C57BL6/J males unless otherwise specified in the corresponding figure legend. $InsR^{flox/flox}$ (006955) and $Il33^{flox/flox}$-eGFP (30619) mice were injected with AAV9-CMV-iCre to generate whole-body knock-out mice, respectively. Age-matched controls were injected with AAV9-CMV-Null (Con). *Leptin* KO (*Ob/Ob*) (000632) mice were obtained from Jackson labs. Mice were fed HFD (60% kcal in fat; D12492; Research Diets, New Brunswick, NJ, USA) or regular chow diet (5058; Lab Diet, St Louis, MO) for 12-16 weeks. An ECHO (Echo Medical Systems) magnetic resonance spectroscopy instrument was used to determine body composition. A limitation of this study is that it was only conducted in male mice since obesity is better modeled in male mice of the C57BL6/J background.

Housing

Mice were maintained at 22-23°C or 30°C on 12 h light/dark cycles (8 AM-8 PM) in the institutional barrier facility along with sentinel cages and were specific pathogen-free. A subset of mice were maintained in complete darkness (24 h dark cycle) for the duration of the study. Mice in sentinel cages are routinely tested by the Institute for Animal Studies (Einstein) for specific pathogens, and health reports were evaluated at regular intervals.

TAN feeding and caloric restriction (CR)

TAN mice were fed only in the two 2 h windows each day (9–11 PM and 5–7 PM) during the nocturnal (dark) mouse cycle. Despite our attempts to pair feed, the net amount of food consumed in the two 2 h windows by TAN mice was ~7% less than the food consumed by the Ad-lib group in the preceding 24 hr. Both cohorts were group housed. The TAN group and its Ad-lib control (Con) included the same number of age and weight-matched male littermate per cage when experiments started. Residual food pellets in the TAN group were carefully collected and weighed at the end of the 2 h feeding period. For caloric restriction experiments we restricted 40% of total day calories compared to the age and weight-matched Ad-lib control group and all the food was given at 9 PM.

METHOD DETAILS

Tissue collection and injections

Mice were humanely euthanized 3 h after the first window of feeding (12 AM, dark phase). Adipose depot samples (sWAT, eWAT and BAT) were frozen at -80°C for bulk RNAseq, qPCR and Western blotting. For some experiments the rest of the tissue was used for SVF isolation and further analysis. Additional samples of adipose depots were harvested for immunohistochemistry and *Seahorse* tissue respirometry. For insulin deficient model, we injected streptozotocin (STZ) in a low dose (40mg/kg)[32] intraperitoneally once-a-day for 11 consecutive days, one month before sacrifice. For insulin (0.16 UI/mouse)[33], leptin (5mg/kg)[34] and co-treatment experiment the injection were performed daily at 9 PM mouse time (dark cycle).

Creation of knock-out models

Whole body knockout for $IL33^{flox/flox}$-eGFP and Insulin $receptor^{flox/flox}$ (*InsR*) was accomplished by retro-orbital injections of $5*10^{11}$ gc/mouse of AAV9-iCre recombinase in 100uL volume (Vector Biolabs, Malvern, PA, USA) when mice were 2-3 mo-old. The feeding intervention was started a week after a single dose of AAV9-iCre. At the end of each experiment, the knock-out of *Il33* was determined by decreased GFP protein levels, since a GFP was inserted into the 3 prime UTR of IL33 sequence, while loss of $InsR^{KO}$ was determined by decreased insulin receptor β protein levels.

Subcutaneous-inguinal white and brown adipose tissue denervation

Mice were anesthetized with isoflurane and maintained at a surgical plane of anesthesia for 20-30 min of the bilateral sWAT or BAT nerve transection procedure. The mouse was shaved and secured on a warm surgical table. A 2 cm skin incision was made along the caudal aspect of the ventrum, and the edges of the skin were retracted laterally and held in place with sterile retractors, exposing the sWAT or BAT depots and adjacent connective tissue. The connective tissues retaining left and right sWAT or BAT depots were bluntly dissected from the skin an underlying peritoneal tissue and skeletal muscle. The caudal and rostral nerve bundles innervating the sWAT depots on each side were exposed, isolated, and transected with micro scissors. For BAT all five branches of intercostal

sympathetic nerves connecting to the right and left BAT fat pads were identified, carefully isolated, and sectioned. After the procedure the skin incision was closed with VetBond adhesive.

Biochemical analyses

Serum insulin (ALPCO, NH, USA), leptin (R&D systems, MN, USA), IL33 (R&D systems, MN, USA), and corticosterone (Immuno-Biological Laboratories Inc.) were assessed using commercial kits according to manufacturer's instructions.

Western blotting

Adipose tissues samples were homogenized in RIPA buffer containing 50 mM Tris, 150 mM NaCl, 1% NP-40, 0.5% sodium deoxycholate, 0.1% SDS, 0.1 mM EDTA, 0.1 mM EGTA, and protease/phosphatase inhibitors. Lysates were centrifuged, and supernatants were subjected to immunoblotting by denaturing 20 μg of protein at 100°C or 50°C, depending on the antibody, for 5 min in Laemmli sample buffer containing 62.5 mM Tris, 2% SDS, 25% glycerol, 0.01% bromophenol blue, and 5% beta-mercaptoethanol. Samples were resolved on SDS-PAGE and transferred to nitrocellulose membranes (GE Healthcare, USA) in transfer buffer containing 25 mM Tris, 192 mM glycine, 0.01% SDS, and 15% methanol using a Bio-Rad semidry transfer cell at 150 mA for 30 min. Ponceau red was imaged for later normalization. Membranes were blocked in 5% non-fat dry milk, 20 mM Tris, 500 mM sodium chloride, and 0.5% Tween-20 for 1 h and probed with primary antibodies overnight at 4°C. Immune complexes were detected using peroxidase-conjugate secondary antibodies and the enzyme substrate ECL. Protein bands were obtained using KwikQuant Digital Western Blot Detection System and analyzed with ImageJ software (NIH, USA).

RNA isolation and qPCR analyses

Total RNA was isolated using the Trizol Reagent (Invitrogen). The aqueous phase containing the RNA was loaded onto a gDNA Eliminator Spin Column (Qiagen, USA) for elimination of genomic DNA, and RNA was isolated using the RNeasy Plus kit (Qiagen) according to manufacturer's instructions. Total RNA (500 to 1000 ug) was reverse transcribed into cDNA using the M-MLV reverse transcriptase (Invitrogen), and quantitative RT-PCR analyses was performed using the Power SYBR Green PCR Master Mix (Applied Biosystems, UK) on a StepOne Plus Real-Time PCR System (Applied Biosystems, UK). For each gene, values were normalized to the expression of the housekeeping gene TATA-binding protein (*Tbp*). The mRNA expression in control samples was considered as 1 and mRNA expression in experimental samples was represented as fold-change compared to expression in Con. All reactions were in duplicate or triplicate. Values were expressed in arbitrary units (a.u).

RNAseq analysis

Library construction, quality control and sequencing

Messenger RNA was purified from total RNA using poly-T oligo-attached magnetic beads. After fragmentation, the first strand cDNA was synthesized using random hexamer primers, followed by the second strand cDNA synthesis using either dUTP for directional library or dTTP for non-directional library. For the non-directional library, it was ready after end repair, A-tailing, adapter ligation, size selection, amplification, and purification. For the directional library, it was ready after end repair, A-tailing, adapter ligation, size selection, USER enzyme digestion, amplification, and purification.

The library was checked with Qubit and real-time PCR for quantification and bioanalyzer for size distribution detection. Quantified libraries will be pooled and sequenced on Illumina platforms, according to effective library concentration and data amount.

Clustering and sequencing

The clustering of the index-coded samples was performed according to the manufacturer's instructions. After cluster generation, the library preparations were sequenced on an Illumina platform and paired-end reads were generated.

Differential expression analysis

Differential expression analysis of two conditions/groups (two biological replicates per condition) was performed using the DESeq2 R package (1.20.0). DESeq2 provide statistical routines for determining differential expression in digital gene expression data using a model based on the negative binomial distribution. The resulting P-values were adjusted using the Benjamini and Hochberg's approach for controlling the false discovery rate. Genes with an adjusted P-value <=0.05 found by DESeq2 were assigned as differentially expressed.

Enrichment analysis of differentially expressed genes

The Reactome database brings together the various reactions and biological pathways of human model species. Reactome pathways with corrected Pvalue less than 0.05 were considered significantly enriched by differential expressed genes. We used clusterProfiler software to test the statistical enrichment of differentially expressed genes in the Reactome pathway.

Circadian analysis and clustering

The differential gene expression analyses were conducted with R packages Limma and EdgeR. Count data from the RNA sequencing pipeline were used. Gene expressed less than 10 counts were excluded. This resulted in a total of 5,733 genes from differential analyses. Differential analyses were based on limma linear regression by contrasting treatment (TAN) and control (ad-lib) groups at the same time point, i.e., TAN 8 AM vs AL 8 AM, and for each comparison, differentially expressed genes as defined by adjusted test P-values. For circadian analyses, dryR package (https://github.com/naef-lab/dryR) was used. Pathway enrichment and gene expression analysis were performed using Express Analyst (https://github.com/xia-lab/ExpressAnalystR.git) and shinyGO (https://github.com/gexijin/shinygo.git) R packages. Protein-protein functional enrichment and networks are generated using Network

enrichment (https://github.com/xia-lab/NetworkAnalystR.git) package using STRING database; Confidence cutoff > 900. Gene count, correlation matrix, and enrichment table source data are documented as Tables S1–S4.

Tissue respirometry

Tissue bioenergetics was determined using a *Seahorse* respirometer [35]. Briefly, adipose depots were collected rapidly after sacrifice, and rinsed with Krebs-Henseleit buffer (KHB) (111 mM NaCl, 4.7 mM KCl, 2 mM $MgSO_4$, 1.2 mM Na_2HPO_4, 0.5 mM carnitine, 2.5 mM glucose and 10 mM sodium pyruvate). Tissue was cut into one small piece (8-13 mg) and quickly transferred to individual wells of a XF24 plate and keep it on buffer. Every tissue piece was stabilized from excessive movement by islet capture screens (Seahorse Bioscience), and 675 μl KHB (containing ATP 2 mM, CoA 0.5 mM, NAD 0.1 mM and sodium pyruvate 2 mM) was added to each well. Digitonin was added to enhance plasma membrane permeability. Basal oxygen consumption rates (OCR) were determined at 37°C according to the following plan: Basal readings recorded every 2 min for 5 readings, followed by exposure to digitonin. Subsequent readings (10) were recorded after 2 min mixing and 2 min rest. Basal OCR values were normalized to individual tissue weights.

Glucose tolerance test (GTT)

Overnight fasted mice were administered 2 g/kg D-glucose by intraperitoneal (i.p.) injection and blood glucose levels were measured before the injection and at indicated time-points post-injection using an Ascensia Contour glucometer (Bayer).

Histological analyses and immunohistochemistry

The histological analyses as previously described.[35] Paraffin-embedded sections (5 mm thick) of formalin-fixed adipose tissues were subjected to Hematoxylin and Eosin (H&E) staining. Sections were analyzed under a Zeiss light microscope at the indicated magnification. Adipocyte area in H&E-stained sections were measured using Image J software (NIH, USA). Adipose tissue paraffin sections were dewaxed and rehydrated. Endogenous peroxidase was treated using 3% hydrogen peroxidase, followed by an antigen retrieval citrate buffer (Vector biolabs). Nonspecific binding sites were blocked using Bioxall (Vector biolabs), and later 2.5% BSA. For immunodetection, sections were incubated for overnight at 4°C, and the specific staining was detected using ImmPRESS-AP Anti-Rabbit Ig Reagent antibody and ImmPACT Vector Red Substrate Kit (Vector biolabs). Sections were counterstained, and in the case of UCP1 rehydrated before mounting. Slides were visualized and images acquired with Zeiss Axiolab 5 microscope.

Depot-wide imaging and quantifications for adipocyte, vasculature and nerve mass

Imaging was carried out using Miltenyi LaVision Ultramicroscope II, running Inspector Pro 7.1.15 software (Miltenyi Biotec, Bergisch Gladbach, Germany) and equipped with Zyla 4.2P-CL10 sCMOS camera (Andor Technology, Belfast, UK), SuperK EXTREME supercontinuum white-light laser EXR-15 (NKT Photonics, Birkerød, Denmark) and MV PLAPO 2XC (Olympus, Tokyo, Japan) objective. Adipose tissue morphology and tyrosine hydroxylase staining was imaged with single-sided illumination, using 9 horizontal focusing steps and at 5 μm interval, resulting in voxel size of 1.5 × 1.5 × 1.5 μm. Data was acquired in two channels, tyrosine hydroxylase staining at 560 ± 20 nm (excitation) and 610 ± 30 nm (emission) wavelength and autofluorescence at 630 ± 15 nm (excitation) and 680 ± 15 nm (emission) wavelength. Images for CD31 staining were acquired at 3 μm interval, resulting in 0.9 × 0.9 × 3 μm voxel size, using 560 ± 20 nm (excitation) and 610 ± 30 nm (emission) wavelength for autofluorescence and adipocyte morphology and 785 ± 12.5 nm (excitation) and 845 ± 22.5 nm (emission) wavelength for CD31. Images were acquired as single tiles.

To identify individual adipocytes, we manually labelled the centers of adipocytes in a small subset of 2D slices randomly sampled from the entire data set. Hereafter, a 2D U-Net (https://doi.org/10.48550/arXiv.1505.04597) was trained on the labelled data to detect adipocyte centers. The trained model was the applied on each 3D volume in a slice-by-slice fashion which segmented out the 3D adipocyte centers. Secondly, A coarse segmentation of the adipocyte membranes, was obtained by calculating a 3D Gaussian gradient magnitude image followed by a set threshold. Finally, to obtain the adipocyte segmentation, the detected adipocyte centers and the coarse membrane segmentation is passed on to seeded 3D watershed segmentation. From the resulting segmentation of individual adipocytes could be counted and their sizes could be estimated.

For the blood vessel and TH nerve segmentation, we use parts of the TubeMap analysis pipeline[36]. Specifically, we applied the small vessel detection part, which relies on 3D tube filters and thresholds for first obtaining a binary vessel segmentation. Hereafter, a skeletonization of the segmentation is obtained via morphological operations and the resulting binary skeleton was converted into a graph. From the vessel segmentation, global features, such as volume fraction, could be estimated directly, and from the graph representation local features such as branch points and individual vessel segment lengths and diameters could be estimated. Finally, the tissue volume of each scanned sample was estimated by simple thresholding of the autofluorescence channel.

Single cell isolation from sWAT SVF

The subcutaneous-inguinal fat pads (sWAT) were collected, and the lymph nodes excluded. The fat was gently minced into fine pieces using a blade and placed in 50 mL tubes containing 1X HBSS (without Mg^{2+} or Ca^{2+}) supplemented with 0.5% BSA. After the sacrifice was done, a Collagenase type II solution (4 mg/ml; Worthington Biochemical, Lakewood, NJ, USA) was added to the tubes and incubated in a rotating shaker (200 rpm) at 37 °C for 30-40 minutes. The digestion was stopped, and the mixture was passed through 100 μm cell strainer and centrifuged at 500 x g for 10 min at 4°C. Red blood Cell lysis was performed with

1x Red Blood cells lysis buffer diluted in deionized water (Tonbo, Bioscience). The cell suspension was filtered through 40μm cell strainer to discard debris, and centrifuged at 500 x g for 10 min at 4°C. The pellet was resuspended collected as stromal vascular fraction (SVF) by centrifugation and re-suspended in FACS buffer for Flow Cytometry analysis or DMEM + 10% FBS for RNA-sequencing.

Flow cytometry

The suspended SVF from sWAT was pipetted in a 96-well plate for staining. Cells were first incubated with Live/Dead dyes and Fc-block for 15 min at 4°C. Then, samples were washed and incubated with extracellular antibodies for 30 min at 4°C. Followed this step, the cells were washed and resuspended in an intracellular/permeabilization buffer (Invitrogen) for 30 min at 4°C, followed by the intracellular staining. All antibodies and their respective channels are shown on Table S1. The pellets were resuspended and acquired by using Cytek Aurora Flow Cytometer and analyzed by BD FlowJo v10.8 software. Flow cytometry gating strategies are shown on Figure S5.

Gating strategy

We highlighted that antibodies conjugated in the same channel were used in different experiments for different assessment and conclusions. Lin- cells were gated for assessing adipocyte progenitors using CD24, Sca-1, PDGFRa and PDGFRB as shown. For some experiments, we substituted PDGFRa on APC for Ki67, and for those, we presented progenitors as PDGFRB+; Sca-1-. Endothelial cells were gated as $CD31^+$. For lymphocyte analysis, we first excluded macrophage markers (CD206-, CD11c-, CD11b-, F4/80-). For macrophages we used CD11B and F480 double positive population. M1-like macrophages expresses CD11C, and M2-like macrophages expresses CD206. For B cells we used B220. For NK cells we used NK1.1 and CD3. From non-B cells we used CD3, CD4, CD8 and Treg for T cells. Non-B and Non-T cells were gated for ILC2 isolation. We considered ILC2 populations cells that were positive for CD25, GATA-3 and ST2. We also analyzed GATA-3 and ST2 single positive cells respectively after gating on CD25 for some experiments/ comparison as indicated, in each figure legend. Ki67 was gated in each population of interest when necessary and indicated in each figure legend.

SVF scRNAseq analysis

SVF single cell barcoding and library preparation

To yield an expected recovery of 4000-7000 single cells, an estimated 10,000 single cells per channel were loaded onto Single Cell 3' Chip (10X Genomics, CA). The Single Cell 3' Chip was placed on a 10X Genomics instrument to generate single cell gel beads in emulsion (GEMs). Chromium Single Cell 3' v3 Library and Cell Bead Kits were used according to the manufacturer's instructions to prepare single cell RNA-Seq libraries.

Illumina high-throughput sequencing libraries

Qubit Fluorometric Quantitation (ThermoFisher, Canoga Park, CA, USA) was used to quantify the 10X Genomics library molar concentration and a TapeStation (Aligent, Santa Clara, CA, USA) was used to estimated library fragment length. Libraires were pooled and sequenced on an Illumina HiSeq 4000 (Illumina, San Diego, CA, USA) with PE100 reads and an 8 bp index read for multiplexing. Read 1 contained the cell barcode and UMI and read 2 contained the single cell transcripts.

Single cell data pre-processing and quality control

To obtain digital gene expression matrices (DGEs) in sparse matrix representation, paired end reads from the Illumina HiSeq 4000 were processed and mapped to the mm10 mouse genome using 10X Genomics' Cell Ranger v3.0.2 software suite. Briefly, .bcl files were demultiplexed and converted to fastq format using the 'mkfastq' function from Cell Ranger. Next, the Cell Ranger 'counts' function mapped reads from fastq files to the mm10 reference genome and tagged mapped reads as either exonic, intronic, or intergenic. Only reads which aligned to exonic regions were used in the resulting DGEs. After combining all four sample DGEs into a single study DGE, we filtered out cells with (a) UMI counts < 700 or > 30,000, (b) gene counts < 200 or > 8,000, and (c) mitochondrial gene ratio > 10%. This filtering resulted in a dataset consisting of 42,052 genes across 12,222 cells, with approximately 2,300 – 4,650 cells from each sample. A median of 2,411 genes and 7,252 transcripts were detected per cell.

Identification of cell clusters

The Seurat R package version 3.0.0.9000 (https://github.com/satijalab/seurat) was used to project all sequenced cells onto two dimensions using t-SNE, and Louvain clustering was used to assign clusters. The optimal number of PCs used for t-SNE dimensionality reduction and Louvain clustering was determined using the Jackstraw permutation approach and a grid-search of the parameters. Similarly, the density used to assign clusters was identified using a parameter grid search. SVF data from this study were all independently normalized using sctransform [37] and integrated using Seurat v3.1.5 [11,38]. The single cell expression profiles were projected into two dimensions using tSNE and the Louvain method for community detection was used to assign clusters. This integrated data was only used to identify and define the cell types.

Cell type-specific gene expression signatures

We defined cell cluster specific marker genes from our Drop-seq dataset using the FindConservedMarkers function in Seurat across all the samples. Briefly, a Wilcoxon Rank Sum Test is run within each sample and a meta P-value across all samples is computed to assess the significance of each gene as a marker for a cluster. Within each sample, the cells are split into two groups: single cells from the cell type of interest and all other single cells. To be considered in the analysis, the gene had to be expressed in at least 10% of the

single cells form one of the groups and there had to be at least a 0.25 log fold change in gene expression between the groups. This process was conducted within each sample separately, and then a meta P-value was assessed from the P-values across all samples. Multiple testing was corrected using the Benjamini-Hochberg method on the meta P-values and genes with an FDR < 0.05 were defined as cell type specific marker genes.

Resolving cell identities of the cell clusters

To identify the cell type identity of each cluster, we used a curated set of canonical marker genes derived from previous studies [20,39–46] to find distinct expression patterns in the cell clusters. Clusters which expressed known marker genes were used as evidence to identify that cell type. Cell subtypes which did not express previously established markers were identified by general cell type markers and those obtained with Seurat's FindConservedMarkers function to define the cell subtype.

Differential gene expression analysis

Within each identified cell type, TAN and Ad-Lib single cells were compared for differential gene expression using Seurat's FindMarkers function (Wilcoxon rank sum test) in a manner similar to Li et al. [47]. Differentially expressed genes were identified using two criteria: (i) an expression difference of >= 1.5-fold and adjusted P-value<0.05 in a grouped analysis between Ad-Lib mice (n = 4) and TAN mice (n = 4); (ii) an expression difference of >= 1.25 fold and consistent fold change direction in all 4 possible pairwise combinations of TAN vs Ad-Lib mice.

Pathway enrichment analysis

Pathway enrichment analysis was conducted on the differentially expressed genes from each cell type using gene sets from Reactome [48]. Prior to enrichment, mouse gene names were converted to human orthologues. Enrichment of pathways was assessed with a Fisher's exact test, followed by multiple testing correction with the Benjamini-Hochberg method. Gene set enrichments with FDR < 0.05 were considered statistically significant.

QUANTIFICATION AND STATISTICAL ANALYSIS

Mean and standard error of mean (S.E.M.) were calculated for each studied variable. Statistical significance was determined using One-way or Two-way ANOVA followed by Bonferroni multiple comparison test or by two-tailed unpaired Student's t-test. *P<0.05, **P<0.01, ***P<0.001. All the statistical analysis was performed using Prism Graph Pad (La Jolla, CA). Statistical details for each experiment including n value are provided in the Figure legend.

ADDITIONAL RESOURCES

None related to this study.

Cell Reports, Volume *43*

Supplemental information

Insulin and leptin oscillations license

food-entrained browning and metabolic flexibility

Pamela Mattar, Andressa Reginato, Christian Lavados, Debajyoti Das, Manu Kalyani, Nuria Martinez-Lopez, Mridul Sharma, Grethe Skovbjerg, Jacob Lercke Skytte, Urmas Roostalu, Rajasekaran Subbarayan, Elodie Picarda, Xingxing Zang, Jinghang Zhang, Chandan Guha, Gary Schwartz, Prashant Rajbhandari, and Rajat Singh

Supplemental Data Title and Legends

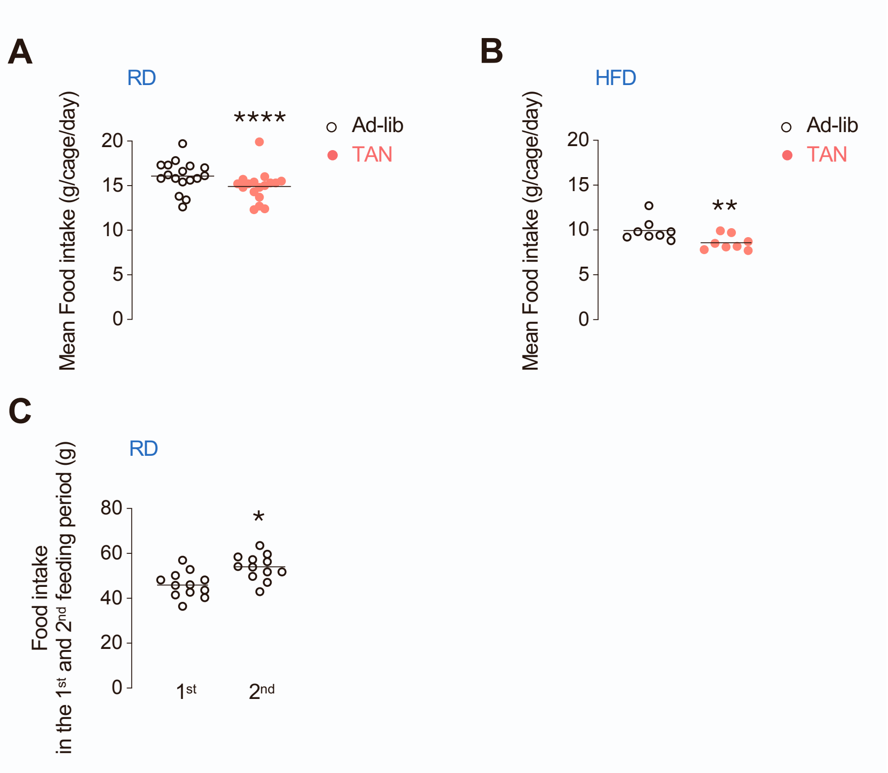

Fig S1 (relates to Fig 1). Food intake assessments in TAN-fed mice.

(**A-B**) Mean food intake (g/cage/day) across each experiment in C57BL6/J male mice fed ad-lib or TAN on RD (n=17) or HFD (n=8).

(**C**) Mean food intake (g) after acclimation in the 1st and 2nd feeding windows in RD-fed C57BL6/J male mice fed ad-lib or TAN (n=12) on RD.

Values are Mean ± SEM. *P<0.05, **P<0.001 ****P<0.0001. Wilcoxon matched-pairs signed rank test (A-C).

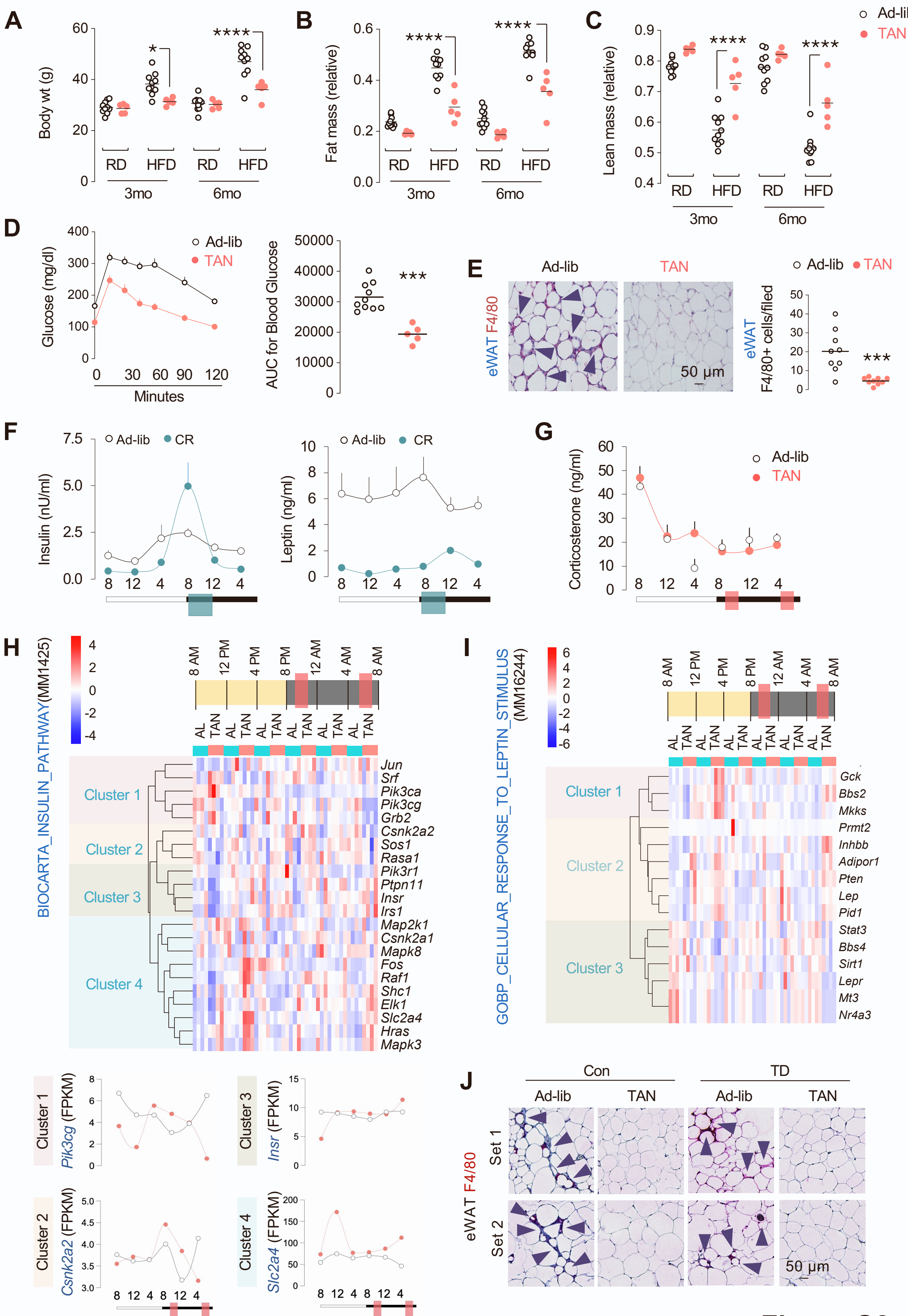

A
Body wt (g)
RD
HFD
3mo
6mo
B
Fat mass (relative)
C
Lean mass (relative)
Ad-lib
TAN
D
Glucose (mg/dl)
Minutes
AUC for Blood Glucose
E
eWAT F4/80
50 μm
eWAT
F4/80+ cells/filed
F
Insulin (nU/ml)
CR
Leptin (ng/ml)
G
Corticosterone (ng/ml)
H
BIOCARTA_INSULIN_PATHWAY(MM1425)
8 AM
12 PM
4 PM
8 PM
12 AM
AL
Cluster 1
Cluster 2
Cluster 3
Cluster 4
Jun
Srf
Pik3ca
Pik3cg
Grb2
Csnk2a2
Sos1
Rasa1
Pik3r1
Ptpn11
Insr
Irs1
Map2k1
Csnk2a1
Mapk8
Fos
Raf1
Shc1
Elk1
Slc2a4
Hras
Mapk3
I
GOBP_CELLULAR_RESPONSE_TO_LEPTIN_STIMULUS
(MM16244)
Gck
Bbs2
Mkks
Prmt2
Inhbb
Adipor1
Pten
Lep
Pid1
Stat3
Bbs4
Sirt1
Lepr
Mt3
Nr4a3
Pik3cg (FPKM)
Insr (FPKM)
Csnk2a2 (FPKM)
Slc2a4 (FPKM)
J
Con
TD
Set 1
Set 2
eWAT F4/80

Figure S2

Fig S2 (relates to Fig 1). Characterizing the protective effect of TAN feeding against obesity, glucose intolerance and inflammation.
(**A-C**) Body weight (g), fat and lean mass (relative to body weight) from C57BL6/J male mice fed ad-lib (n=10) or TAN (n=5) on RD or HFD for 3 or 6 mo.
(**D**) Glucose tolerance test (GTT) and AUC (area under curve) in C57BL6/J male mice fed ad-lib (n=10) or TAN (n=5) on HFD for 6 mo.
(**E**) Representative F4/80 (red) staining in eWAT from C57BL6/J male mice fed ad-lib or TAN on HFD for 6 mo (n=9 each group). Quantification for number of F4/80+ cells/field (1 section/mouse observed with 5X magnification).
(**F**) Serum insulin and leptin levels across 6 time-points each day (24 h) in C57BL6/J male mice in 12 h/12 h light/dark cycle fed ad-lib or caloric restricted (CR, 40% restriction) on RD for 5 mo. Feeding window is label in Y-axis (blue boxes) (n=5 each group).
(**G**) Serum corticosterone levels at 6 time-points in C57BL6/J male mice in 12 h/12 h light/dark cycle fed ad-lib or TAN for 6 mo (n=5 each group). Feeding windows are indicated by *salmon-colored* boxes.
(**H, I**) Bulk RNA-seq analysis of sWAT of ad-lib and TAN-fed mice at 6 Zeitgeber (ZT) time points. Period-wide cluster map of genes associated with insulin pathway (BIOCARTA) **(H)** or response to leptin (GO_BP) **(I)** for both ad-lib and TAN group (n=4 each group). Z-score normalized values were plotted and implemented for hierarchical clustering. Red denotes upregulation, blue denotes downregulation.
(J) Representative F4/80 (red) staining in eWAT of C57BL6/J male mice fed ad-lib or TAN on HFD for 4 mo and housed in 12-12 h light/dark cycle (Con) or 24 h total darkness (TD) (n=5 each group). Quantification for number of F4/80+ cells/field (1 section/mouse observed with 5X magnification).
Dot plots show individual values (dots) and mean (line). Every plot across 24 h shows the mean value (dots) per time-point ± SEM. *P<0.05, ***P<0.001 ****P<0.0001. Two-way ANOVA and Tukey-corrected (A, B, C), and two-tailed unpaired Student's t-test (D, E).

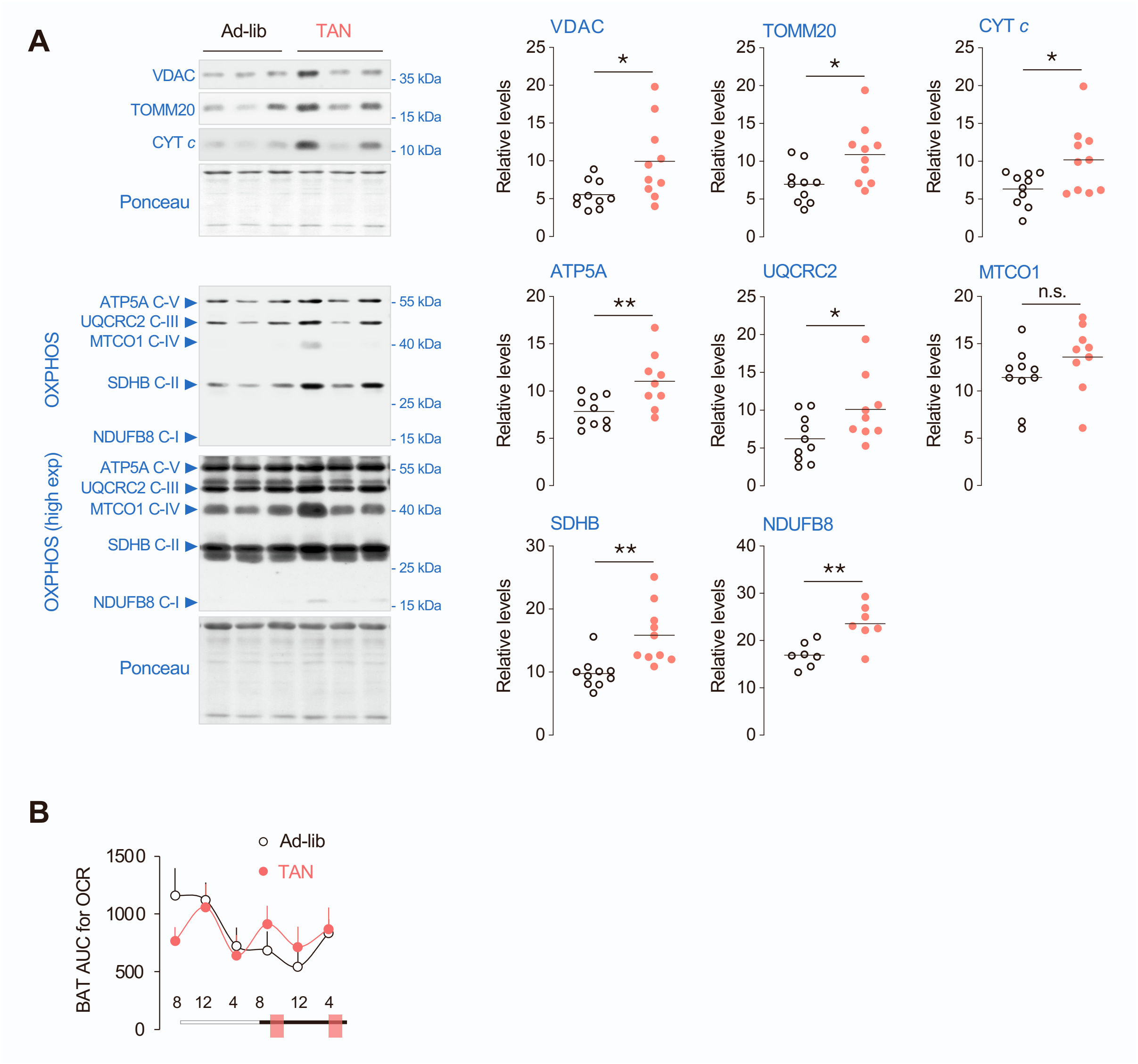

Fig S3 (relates to Fig 3). The impact of TAN feeding on mitochondrial mass and OXPHOS in sWAT and OCR in BAT.

(A) Immunoblots (IB) for indicated mitochondrial markers and OXPHOS components, and their corresponding quantifications in sWAT from C57BL6/J male mice fed ad-lib (n=10) or TAN (n=10) on RD for 5 mo. Ponceau is loading control. Dot plots show individual values (dots) and mean (line). *P < 0.05 and **P<0.01 for two-tailed unpaired Student's t-test.

(B) AUC for OCR in BAT at 6 time-points across 24 h from C57BL6/J male mice fed ad-lib (n=5) or TAN-fed (n=5) on RD for 6 mo. Feeding windows are indicated by *salmon-colored* boxes. The plot across 24h show the mean values (dots) per timepoint and ±SEM.

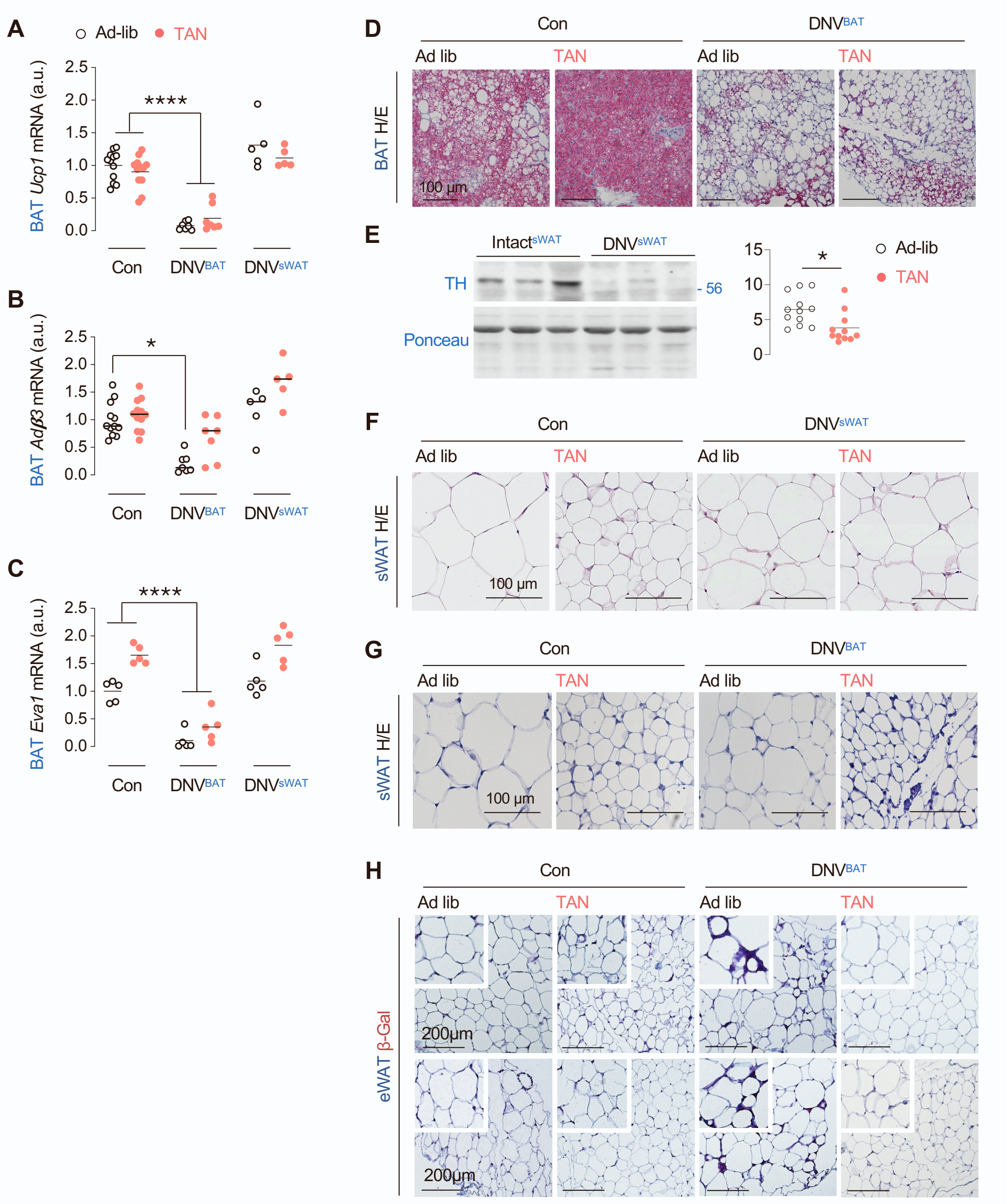

Fig S4 (relates to Fig 4). Confirmation of denervations of BAT and sWAT.
(A-C) qPCR for indicated genes in BAT and their quantifications are shown in innervated control (Con) or denervated sWAT (DNV^{sWAT}) or BAT (DNV^{BAT}) from C57BL6/J male mice fed ad-lib (n=5-12 Con, n=5-7 DNV^{BAT}, n=5 DNV^{sWAT}) or TAN (n=5-12 Con, n=5-7 DNV^{BAT}, n=5 DNV^{sWAT}) on HFD for 3 mo.
(D) Representative H&E staining of BAT from Con and DNV^{BAT} C57BL6/J male mice fed ad-lib or TAN on HFD for 3 mo.
(E) Immunoblots (IB) for Tyrosine hydroxylase (TH) (and corresponding quantifications) in sWAT from Con (n=11) or DNV^{sWAT} (n=11) C57BL6/J male mice fed ad-lib or TAN on HFD for 3 mo.
(F, G) Representative H&E staining of sWAT from Con and DNV^{sWAT} **(F)**, and Con and DNV^{BAT} **(G)** C57BL6/J male mice fed ad-lib or TAN on HFD for 3 mo.
(H) Representative b-galactosidase staining in eWAT from Con or DNV^{BAT} C57BL6/J male mice fed ad-lib or TAN on HFD for 3 mo.
Dot plots show individual values (dots) and mean (line). *P<0.05, ****P<0.0001. Two-way ANOVA and Tukey-corrected.

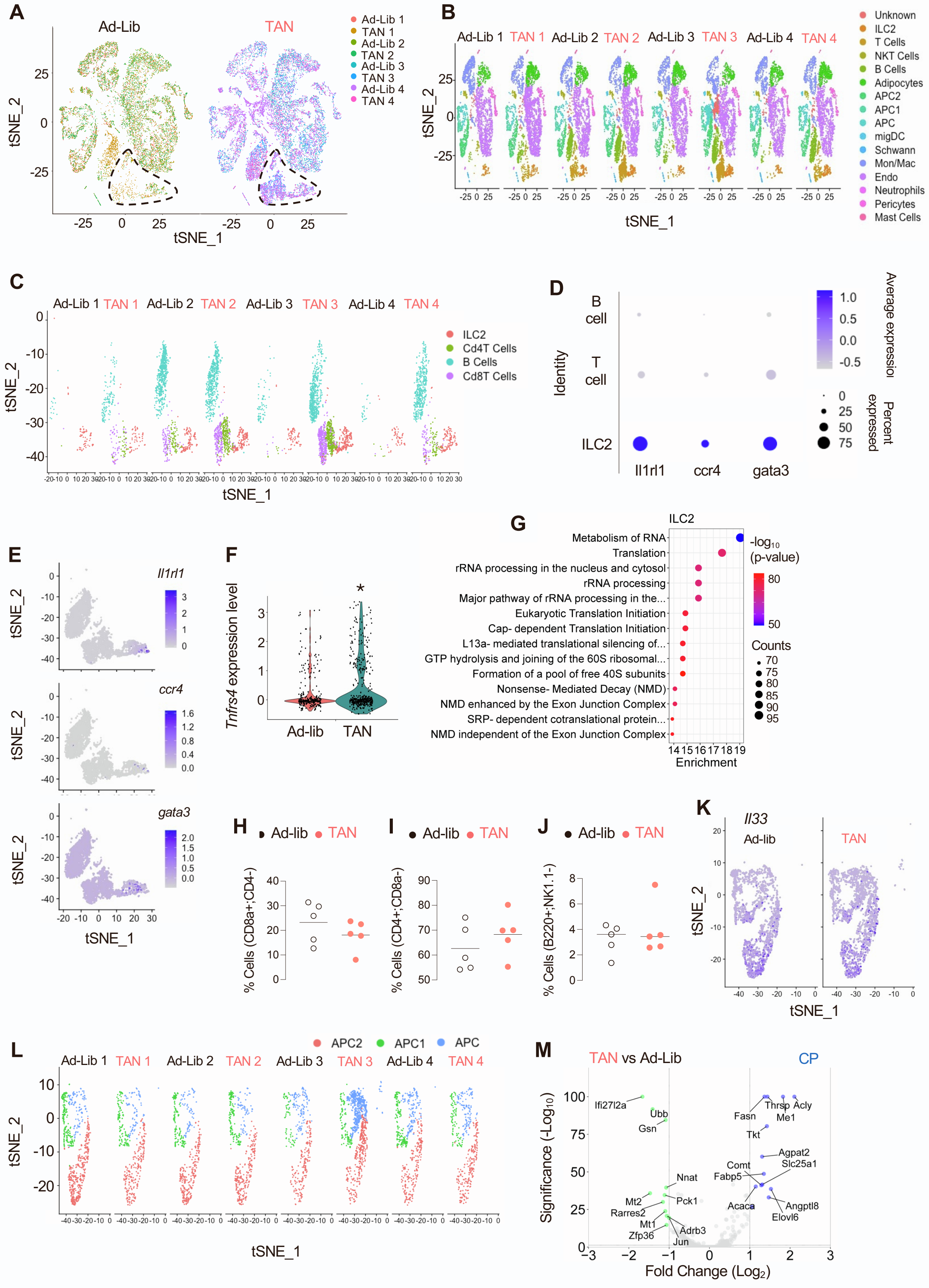
A
Ad-Lib
TAN
Ad-Lib 1
TAN 1
Ad-Lib 2
TAN 2
Ad-Lib 3
TAN 3
Ad-Lib 4
TAN 4
tSNE_2
tSNE_1
B
Unknown
ILC2
T Cells
NKT Cells
B Cells
Adipocytes
APC2
APC1
APC
migDC
Schwann
Mon/Mac
Endo
Neutrophils
Pericytes
Mast Cells
C
ILC2
Cd4T Cells
B Cells
Cd8T Cells
D
B cell
T cell
ILC2
Identity
Il1rl1
ccr4
gata3
Average expression
Percent expressed
E
Il1rl1
ccr4
gata3
F
Tnfrs4 expression level
Ad-lib
TAN
G
ILC2
Metabolism of RNA
Translation
rRNA processing in the nucleus and cytosol
rRNA processing
Major pathway of rRNA processing in the...
Eukaryotic Translation Initiation
Cap- dependent Translation Initiation
L13a- mediated translational silencing of...
GTP hydrolysis and joining of the 60S ribosomal...
Formation of a pool of free 40S subunits
Nonsense- Mediated Decay (NMD)
NMD enhanced by the Exon Junction Complex
SRP- dependent cotranslational protein...
NMD independent of the Exon Junction Complex
Enrichment
-log10 (p-value)
Counts
H
Ad-lib
TAN
% Cells (CD8a+;CD4-)
I
% Cells (CD4+;CD8a-)
J
% Cells (B220+;NK1.1-)
K
Il33
L
APC2
APC1
APC
M
TAN vs Ad-Lib
CP
Significance (-Log10)
Fold Change (Log2)
Ifi27l2a
Ubb
Gsn
Nnat
Pck1
Mt2
Rarres2
Mt1
Zfp36
Adrb3
Jun
Fasn
Thrsp
Acly
Me1
Tkt
Agpat2
Slc25a1
Comt
Fabp5
Acaca
Angptl8
Elovl6

Figure S5

Fig. S5 (relates to Fig 5). scRNAseq analyses of sWAT stromal vascular fractions from ad-lib and TAN-fed mice.

(A-B) t-SNE plots showing clustering of cells from each sWAT SVF replicate of C57BL6/J male mice fed ad-lib or TAN for 5 mo on RD, n=4 each group.

(C) t-SNE sub-clustering of immune cells from each sWAT SVF replicate of C57BL6/J male mice fed ad-lib or TAN for 5 mo on RD, n=4 each group.

(D-E) Identity analysis **(D)** and t-SNE graphs **(E)** for expression of indicated ILC2 cell markers. Interleukin 1 receptor-like 1 (*Il1ri1*), C-C Motif Chemokine Receptor 4 (*ccr*4) and Trans-acting T-cell-specific transcription factor GATA-3 (*gata3*). n=4 each group.

(F) Expression of TNF receptor superfamily member 4 (*Tnfrsf4*) in ILC2 population from C57BL6/J male mice fed ad-lib or TAN for 5 mo on RD, n=4 each group.

(G) Bubble plot based on Reactome biological pathways showing the top 14 up-regulated pathways in ILC2 population in Fig 5C. Bubble size represents number of genes/pathway, Y-axis represents the percentage of enrichment and bubble color represents the $-\log_{10}$ P-value (n=4 each group).

(H-J) Quantification for indicated immune cells in sWAT SVF from C57BL6/J male fed ad-lib or TAN on RD for 5 mo (n=5 each group).

(K) t-SNE graphs for APC sub-clustering showing expression of *Il33* in C57BL6/J male mice fed ad-lib or TAN on RD for 5 mo (n=4 each group).

(L) t-SNE graphs for APC sub-clustering for each replicate from C57BL6/J male mice fed ad-lib or TAN on RD for 5 mo (n=4 each group).

(M) Volcano plot showing up- and down-regulated genes in sWAT Committed Preadipocytes (CP) in TAN-fed mice compare to ad-lib mice (n=4).

Dot plots show individual values (dots) and mean (line). n.s.=not significant for two-tailed unpaired Student's *t*-test (H, I, J).

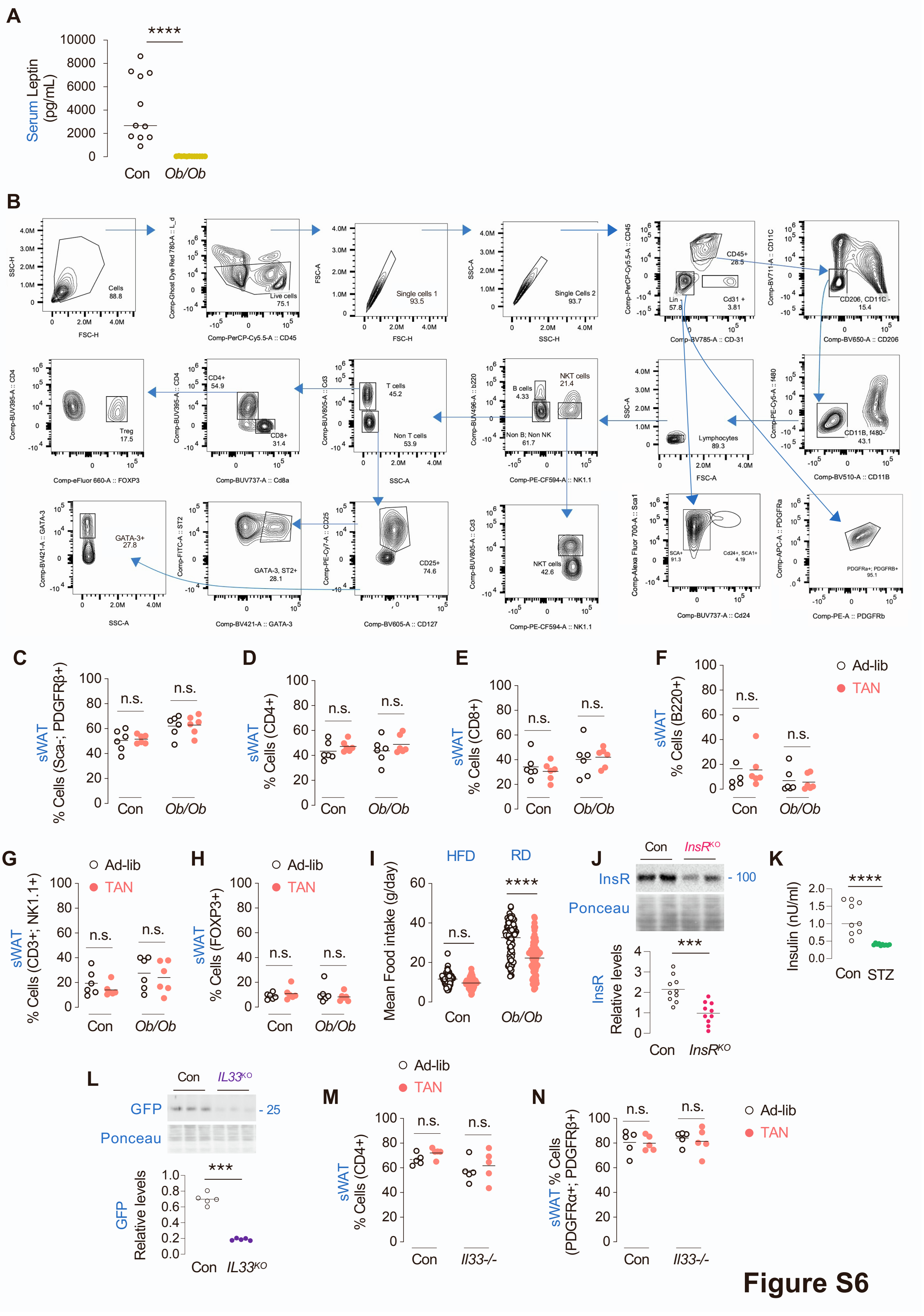

A
Serum Leptin (pg/mL)
****
Con
Ob/Ob
B
Cells 88.8
Live cells 75.1
Single cells 1 93.5
Single Cells 2 93.7
CD45+ 28.5
Lin - 57.8
Cd31 + 3.81
CD206, CD11C - 15.4
Lymphocytes 89.3
CD11B, f480- 43.1
NKT cells 21.4
B cells 4.33
Non B; Non NK 61.7
T cells 45.2
Non T cells 53.9
CD4+ 54.9
CD8+ 31.4
Treg 17.5
GATA-3+ 27.8
GATA-3, ST2+ 28.1
CD25+ 74.6
NKT cells 42.6
SCA+ 91.3
Cd24+, SCA1+ 4.19
PDGFRa+; PDGFRB+ 95.1
C
sWAT % Cells (Sca-; PDGFRβ+)
D
sWAT % Cells (CD4+)
E
sWAT % Cells (CD8+)
F
sWAT % Cells (B220+)
Ad-lib
TAN
n.s.
G
sWAT % Cells (CD3+; NK1.1+)
H
sWAT % Cells (FOXP3+)
I
Mean Food intake (g/day)
HFD
RD
J
Con
InsR^KO
InsR
Ponceau
- 100
InsR Relative levels
***
K
Insulin (nU/ml)
Con
STZ
L
Con
IL33^KO
GFP
Ponceau
- 25
GFP Relative levels
M
sWAT % Cells (CD4+)
Il33-/-
N
sWAT % Cells (PDGFRα+; PDGFRβ+)

Figure S6

Fig S6 (relates to Fig 6). Validations of KO mice models, and characterizing the effect of TAN feeding on immune cells from sWAT SVF of *Ob/Ob* mice.

(A) Serum leptin levels in Con (n=11) and *Ob/Ob* mice (n=12).

(B) Gating strategy to identify different immune cell populations in sWAT SVF.

(C-H) Quantification for progenitors (**C**), $CD4^+$ (**D**), $CD8^+$ (**E**), B220+ cells (**F**), NK1.1+ (**G**) and FOXP3+ Treg (**H**) cells in sWAT SVF from C57BL6/J control fed HFD or *Ob/Ob* fed on RD and subjected to ad-lib or TAN for 3 mo (n=6 each group).

(I) Mean food intake (g/cage/day) in Con (n=11) and *Ob/Ob* mice (n=12) for the duration of the study. Each dot represents food consumed in grams (g) per cage per day.

(J) IB and densitometric quantification for Insulin receptor (InsR) in liver from C57BL6/J male Con and whole-body $InsR^{KO}$ mice (n=10 each group).

(K) Serum insulin levels in C57BL6/J male Con (n=9) and STZ-injected (n=10) mice fed RD for 5 mo.

(L) IB for Green Fluorescence Protein (GFP) and densitometric quantification in liver from C57BL6/J male control or whole-body $Il33^{KO}$ mice fed HFD for 3 mo (n=5 each group).

(M-N) Quantification for CD4+ cells **(M)** and progenitor cells (**N**) in sWAT SVF from C57BL6/J male control or whole-body $Il33^{KO}$ mice fed ad-lib or TAN (n=5 each group).

Dot plots show individual values (dots) and mean (line). n.s.=not significant, ***$P<0.001$, ****$P<0.0001$. Two-way ANOVA and Tukey-corrected (C, D, E, F, G, H, I, M, N), and two-tailed unpaired Student's *t*-test (A, J, K, L).

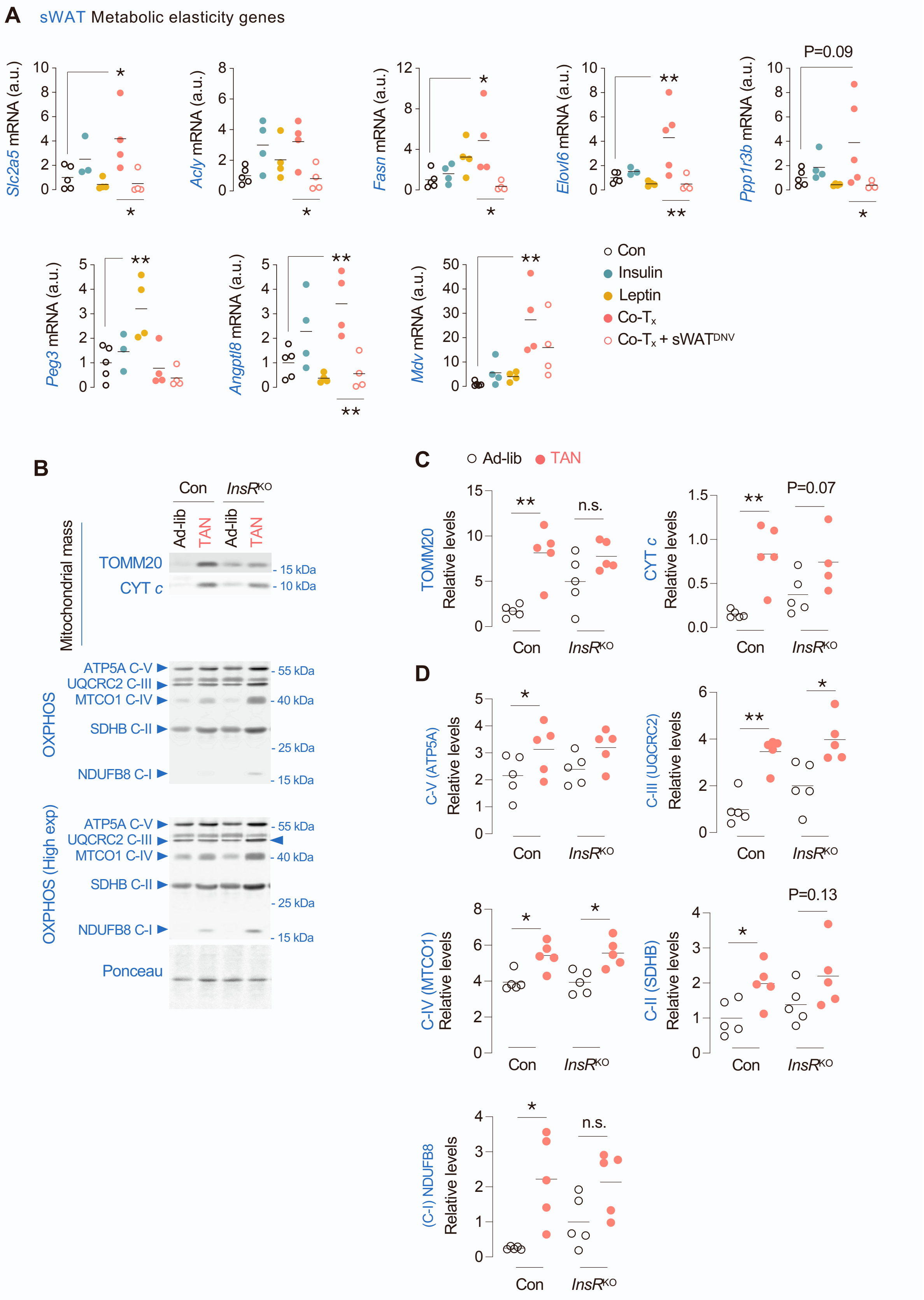
A sWAT Metabolic elasticity genes
Slc2a5 mRNA (a.u.)
Acly mRNA (a.u.)
Fasn mRNA (a.u.)
Elovl6 mRNA (a.u.)
Ppp1r3b mRNA (a.u.)
P=0.09
Peg3 mRNA (a.u.)
Angptl8 mRNA (a.u.)
Mdv mRNA (a.u.)
Con
Insulin
Leptin
Co-Tx
Co-Tx + sWATDNV
B
Con
InsRKO
Ad-lib
TAN
Mitochondrial mass
TOMM20
CYT c
- 15 kDa
- 10 kDa
OXPHOS
ATP5A C-V
UQCRC2 C-III
MTCO1 C-IV
SDHB C-II
NDUFB8 C-I
- 55 kDa
- 40 kDa
- 25 kDa
- 15 kDa
OXPHOS (High exp)
Ponceau
C
Ad-lib
TAN
TOMM20 Relative levels
CYT c Relative levels
n.s.
P=0.07
D
C-V (ATP5A) Relative levels
C-III (UQCRC2) Relative levels
C-IV (MTCO1) Relative levels
C-II (SDHB) Relative levels
P=0.13
(C-I) NDUFB8 Relative levels

Figure S7

Fig. S7 (relates to Fig 7). Impact of injections of insulin and leptin on metabolic flexibility genes in sWAT and the effect of loss of insulin receptor signaling on benefits of TAN feeding.
(A) qPCR for indicated metabolic elasticity genes in sWAT from C57BL6/J male mice fed ad-lib on RD and injected with the indicated hormones for 1.5 mo. Con (vehicle-injected, n=5), insulin (n=4), leptin (n=4), Co-T_x (insulin and leptin-injected, n=4-5) and DNV^{sWAT} co-T_x (n=4).
(B-D) Representative IB and quantifications for indicated mitochondrial markers and OXPHOS components in sWAT from Con or $InsR^{KO}$ male mice fed ad-lib (n=5 Con, n=5 $InsR^{KO}$) or TAN (n=5 Con, n=5 $InsR^{KO}$) for 5 mo. Ponceau is loading control.
Dot plots show individual values (dots) and mean (line). n.s.=not significant, *P<0.05, **P<0.01. Two-way ANOVA and Tukey-corrected.

Supplemental Video Title and Legends

Supplemental Video 1. 3D visualization of sWAT from a representative ad-lib-fed mouse. Adipocyte volume (0-100,000 μm3) is color-coded. Tissue autofluorescence is grey scale.
Supplemental Video 2. 3D visualization of sWAT from a representative, TAN-fed mouse. Adipocyte volume (0-100,000 μm3) is color-coded. Tissue autofluorescence is grey scale.
Supplemental Video 3. 3D visualization of CD31 staining (red) in sWAT from a representative ad-lib-fed.
Supplemental Video 4. 3D visualization of CD31 staining (red) in sWAT from a representative TAN-fed.

Supplemental Table Title and Legends

Supplemental Table 1: Pathway enrichment analysis using BIOCARTA for top-100 upregulated genes in sWAT of TAN versus ad-lib-fed mice on RD for 6 mo.
Supplemental Table 2: Limma DEG analysis for all 6 time points in sWAT of TAN versus ad-lib-fed mice on RD for 6 mo.
Supplemental Table 3: Gene_FPKM and Candidate_gene_FPKM for all 6 time points in sWAT of TAN versus ad-lib-fed mice on RD for 6 mo.
Supplemental Table 4: Metabolic Plasticity FPKM Correlation Enrichment network for all 6 time points in sWAT of TAN versus ad-lib-fed mice on RD for 6 mo.

Supplemental Table 5: Primers for real-time PCR analysis related to STAR Methods

Primers for real-time PCR analysis related to STAR Methods

GENE	PROTEIN	PRIMERS
Adrb3	Adrenergic receptor, beta 3	(f) 5'- ggcaacctgctggtaatcat-3'<br>(r) 5'- tccactgacgtccacagttc-3'
Eva1a	Eva-1 homolog A, regulator of programmed cell death	(f) 5'- ccacttctcctgagtttacagc-3'<br>(r) 5'- gcattttaaccgaacatctgtcc-3'
Tbp	TATA box binding protein	(f) 5'- gaagctgcggtacaattccag-3'<br>(r) 5'- ccccttgtacccttcaccaat-3'
Slc2a5	Solute carrier family 2 (facilitated glucose transporter), member 5	(f) 5'- cgaaaaacctacgaggggct-3'<br>(r) 5'- ctggccagccatcctcattt-3'
Acly	ATP citrate lyase	(f) 5'- cgtcgaggaaagcttgg-3'<br>(r) 5'- tgagaaagttcttgaggaagc-3'
Fasn	Fatty acid synthase	(f) 5'- ctgactcggctactgacacg-3'<br>(r) 5'- tgagctgggttagggtagga-3'
Elovl6	ELOVL fatty acid elongase 6	(f) 5'- cgtagcgactccgaagatcagcc-3'<br>(r) 5'- agcgtacagcgcagaaaacagga -3'
Ppp1r3b	Protein phosphatase 1, regulatory subunit 3B	(f) 5'- actgtgtgctgaaggataagg-3'<br>(r) 5'- cacatactgacaagggaaatct-3'
Peg3	Paternally Expressed 3	(f) 5'- agtccagcttgccgaagat-3'<br>(r) 5'- ctcaggcatgggtttgagac-3'
Lep	Leptin	(f) 5'- tctccgagacctcctccatct-3'<br>(r) 5'- ttccaggacgccatccag-3'
Angptl8	Angiopoietin-like 8	(f) 5'- ctcaatggcgtgtacagagc-3'<br>(r) 5'- tcgaaggtgtaaagcgtcct -3'
Mvd	Mevalonate (diphospho) decarboxylase	(f) 5'- agtccagcttgccgaagat-3'<br>(r) 5'- ctcaggcatgggtttgagac-3'
